# Spiralian gastrulation: germ layer formation, morphogenesis, and fate of the blastopore in the slipper snail *Crepidula fornicata*

**DOI:** 10.1186/s13227-015-0019-1

**Published:** 2015-06-24

**Authors:** Deirdre C. Lyons, Kimberly J. Perry, Jonathan Q. Henry

**Affiliations:** Biology Department, Duke University, 124 Science Drive, Durham, NC 27708 USA; Department of Cell and Developmental Biology, University of Illinois, 601 S. Goodwin Ave., Urbana, IL 61801 USA

**Keywords:** Spiralia, Lophotrochozoa, Blastopore, Ectomesoderm, Endomesoderm, Epiboly, Gastrulation, Amphistomy

## Abstract

**Background:**

Gastrulation is a critical step in bilaterian development, directly linked to the segregation of germ layers, establishment of axes, and emergence of the through-gut. Theories about the evolution of gastrulation often concern the fate of the blastopore (site of endomesoderm internalization), which varies widely in a major branch of bilaterians, the Spiralia. In this group, the blastopore has been said to become the mouth, the anus, both, or neither. Different developmental explanations for this variation exist, yet no modern lineage tracing study has ever correlated the position of cells surrounding the blastopore with their contribution to tissues of the mouth, foregut, and anus in a spiralian. This is the first study to do so, using the gastropod *Crepidula fornicata*.

**Results:**

*Crepidula* gastrulation occurs by epiboly: the first through third quartet micromeres form an epithelial animal cap that expands to cover vegetal endomesodermal precursors. Initially, descendants of the second and third quartet micromeres (2a–2d, 3a–3d) occupy a portion of the blastopore lip. As the blastopore narrows, the micromeres’ progeny exhibit lineage-specific behaviors that result in certain sublineages leaving the lip’s edge. Anteriorly, cells derived from 3a^2^ and 3b^2^ undergo a unique epithelial-to-mesenchymal transition involving proliferation and a collective movement of cells into the archenteron. These cells make a novel spiralian germ layer, the ectomesoderm. Posteriorly, cells derived from 3c^2^ and 3d^2^ undergo a form of convergence and extension that involves zippering of cells and their intercalation across the ventral midline. During this process, several of these cells, as well as the 2d clone, become displaced posteriorly, away from the blastopore. Progeny of 2a-2c and 3a-3d make the mouth and foregut, and the blastopore becomes the opening to the mouth. The anus forms days later, as a secondary opening within the 2d^2^ clone, and not from the classically described “anal cells”, which we identify as the 3c^221^ and 3d^221^ cells.

**Conclusions:**

Our analysis of *Crepidula* gastrulation constitutes the first description of blastopore lip morphogenesis and fates using lineage tracing and live imaging. These data have profound implications for hypotheses about the evolution of the bilaterian gut and help explain observed variation in blastopore morphogenesis among spiralians.

**Electronic supplementary material:**

The online version of this article (doi:10.1186/s13227-015-0019-1) contains supplementary material, which is available to authorized users.

## Background

Gastrulation accomplishes several tasks that are critical for metazoan development, most importantly the segregation of germ layers and re-arrangement of cells to form the basic body-plan [1]. Germ layer segregation occurs when ectodermal, endodermal, and mesodermal cells adopt distinct gene regulatory states; morphogenetic events internalize endodermal and mesodermal cell types. Because the basic body-plan is often established by the end of gastrulation, theories explaining body-plan evolution often suggest that body-plan divergence was driven in part by modifications to gastrulation events themselves (e.g*.*, [2–5] and references therein).

For example, several evolutionary theories concern the site of gastrulation, a transient embryonic location where endoderm and mesoderm are internalized, called the blastopore [2, 3, 5–7]. The fate of the blastopore was traditionally used as a character for building taxonomic trees, separating protostomes (in which the blastopore becomes the opening to the mouth) from deuterostomes (in which the mouth forms separately from the blastopore, and the latter often becomes the anus) (reviewed in [2, 6–8]). Although the terms deuterostome and protostome are still in our vernacular, they have lost much of their phylogenetic significance because cladistic analyses based on molecular characters (which are independent of morphology) show that protostomy and deuterostomy are not a reliable diagnostic character for major clades of bilaterians. Deuterostomy appears to be ancestral for the Ambulacaria and Chordata [1, 9], but among the groups traditionally thought to belong to a protostome branch—the Ecdysozoa and Spiralia (which includes the Lophotrochozoa)—the fate of the blastopore is more complex and variable [7, 10, 6, 11]. Depending on the species, the blastopore has been reported to become the mouth (protostomy), the anus (deuterostomy), both mouth and anus (amphistomy), or neither (i.e., the blastopore closes). Debate remains about how this diversity in blastopore fate arose, whether the differences have any influence on body-plan divergence, and even if the blastopore can be properly homologized between distantly related animals [2, 5, 7, 8, 10, 12, 13]. To resolve these debates, it is necessary to compare gastrulation in multiple species, in the context of a solid phylogenetic framework.

Spiralian lophotrochozoans offer a means to address many of these questions because although the fate of the blastopore varies between species, they share a stereotyped early cleavage pattern and fate map [14, 15]. These attributes make it possible to identify unambiguously homologous cell lineages, allowing direct comparison of cells around the blastopore. Yet, few modern studies have leveraged spiralians’ unique strengths for investigating gastrulation [16–19]. Here, we study gastrulation in a representative spiralian species, the slipper snail *Crepidula fornicata*.

The Spiralia is a large and morphologically diverse assemblage of roughly 14 phyla [20, 21, 15]. Many members of this group, including annelids, molluscs, platyhelminthes, and nemerteans, exhibit a highly conserved pattern of embryonic cell divisions termed “spiral cleavage” [15, 14]. The fate map, birth order, and geometry of cells are so conserved between these animals that it is possible to compare homologous lineages, with single-cell resolution, between spiralians with vastly different larval and adult morphologies. During gastrulation, spiralians transition from the highly conserved spiralian cleavage program to their species-specific body-plans. Thus, spiralians are an excellent group for testing if there is indeed a relationship between varying gastrulation modes (e.g., the fate of the blastopore) and body-plan evolution. Furthermore, because spiral cleavage is considered ancestral for the Spiralia as a whole [14], studying gastrulation in these species can inform us about how gastrulation, and body-plans, might have evolved in metazoans.

The blastopore is important to these arguments because, typically, it is ontogenetically linked to the openings of the digestive tract [2, 5, 7, 8, 10, 22, 23]. For example, in anthozoan cnidarians and ctenophores, the blastopore forms at the embryonic animal pole and matures into a dual-function, single orifice for feeding and excretion [24]. In deuterostomes, the blastopore forms at the vegetal pole and matures into the anus, while the mouth forms later, at a separate site within ventral anterior ectoderm [9]. Among the Ecdysozoa and Spiralia, the blastopore also forms at the vegetal pole, but its subsequent morphogenesis and fate is more complex and variable [6–8, 10, 12, 25–28], complicating discussions about the ancestral mode of gastrulation in these groups. Compounding this problem is the fact that most of the work on spiralian gastrulation is based on classical descriptions from over 100 years ago [5, 7, 8, 29], long before intracellular lineage tracing or time-lapse imaging were possible. The accuracy of these original descriptions comes into question, especially when concerning with the relationship between the blastopore and the formation of the mouth and anus; both can form days after the blastopore exists, making it very difficult to follow cell clones without modern intracellular lineage tracing. Only a few previous lineage tracing studies have examined gastrulation in spiralians, most notably in the leech *Helobdella robusta* [16], and in the snail *Ilyanassa obsoleta* [18]. However, these studies did not focus on the behavior or fate of the blastopore per se.

The slipper snail *C. fornicata* is an emerging model system for developmental and evolutionary studies in spiralians [30–34]. Previously, a fate map was generated for every cell present in the four-principle quartets of animal micromeres, and the vegetal macromeres, for their respective contributions to the tissues of the veliger larva [31]. Here, we used lineage tracing, and time-lapse imaging, to present the first detailed examination of germ layer formation and morphogenesis of cells surrounding the blastopore during gastrulation in *Crepidula*. Gastrulation occurs by epiboly, as animal cap micromeres expand to cover vegetal territories. Later, the vegetal endodermal cells re-arrange to form a cavity that becomes part of the embryonic digestive tract, the archenteron. To make it possible to directly homologize gastrulation events between species, we distinguish between the blastopore and blastopore lip. At early epiboly stages, we define the blastopore as the endoderm/mesoderm cells themselves, and later as the hole/lumen of the archenteron. We define the blastopore lip as those cells that give rise exclusively to ectodermal cells that are initially in direct contact with the endodermal/endomesodermal cells. Here, we document specific lineage contributions to the anterior and posterior ends of the digestive tract (including mouth, foregut, and anus), which are derived from cells of the second and third quartet micromeres that occupied the blastopore lip. In addition, we found that ectomesoderm arises from specific third quartet cells (3a^2^ and 3b^2^) that are situated at the anterior blastopore and become internalized by an epithelial to mesenchymal transition. During gastrulation, blastopore lip cells exhibit extensive cell re-arrangement. The posterior lip of the blastopore closes by a form of convergence and extension that involves zippering of cells derived from the third quartet (from 3c^2^ and 3d^2^). The cells that undergo convergence and extension give rise to a line of ciliated ectodermal cells along the ventral midline, which we argue includes cells akin to the neurotroch and telotroch described in other spiralians. The two posterior-most ciliated cells derived from these clones on the ventral midline were classically described by Conklin [34] as the “anal cells.” However, we found that these cells do not give rise to the anus, and so we renamed them the “terminal cells.” The anus arises from progeny of 2d^2^, and as a result of convergence and extension, these cells become excluded from the posterior blastopore lip. The anus thus forms from a secondary opening, and not from the blastopore, at 12 days of development. The blastopore becomes the opening to the mouth. We discuss the implications of these results for debates about the evolution of the blastopore in metazoans.

## Methods

### Animal care and handling

Stacks of adult *C. fornicata* were obtained from the Marine Resources Department at the Marine Biological Laboratory (Woods Hole, MA. USA). Adults were obtained from local waters by dredging during late winter months (January to March) and maintained in cold running seawater at approximately 12 °C to prevent egg laying. The gravid females are stimulated to lay eggs by transferring them to warm water sea tables at 18–22 °C, as needed throughout the summer. Embryos were obtained and reared, as previously described [30–34]. Briefly, the de-capsulated eggs and embryos were raised at room temperature (approx. 20 °C) in gelatin-coated Petri dishes containing 0.2-μm-filtered seawater with penicillin (100 U/ml, Sigma, St Louis, MO) and streptomycin sulfate (200μg/ml, Sigma, St Louis, MO).

### Lineage tracing

**Fig. 1:**
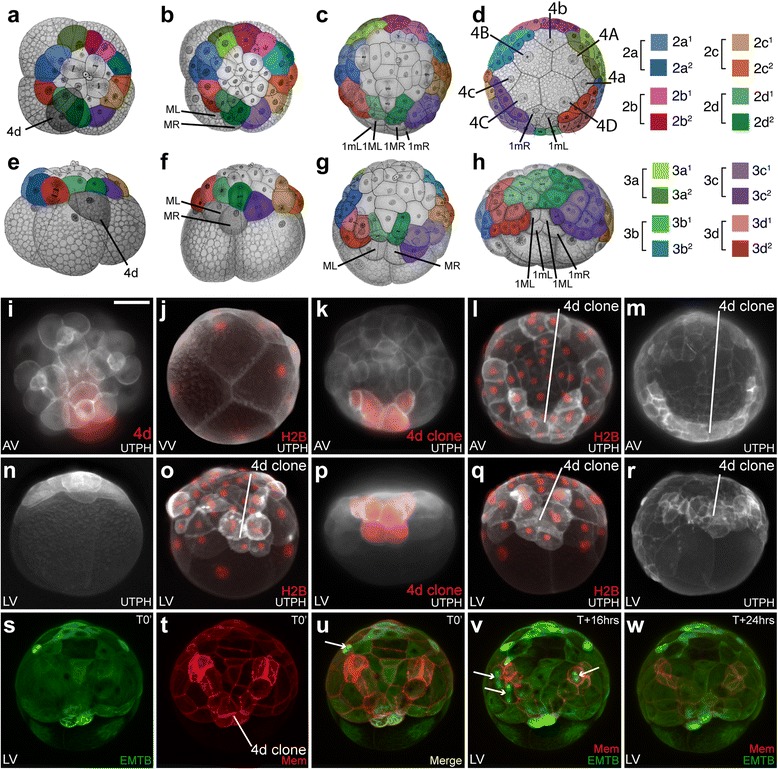
Early epiboly and position of clones at the blastopore lip. **a–h** Cartoons of early embryo with second and third quartet micromeres colored, as indicated in key to the right. **a–c** Animal pole views; **d** is a ventral/vegetal view; **e–h** are lateral views. Black and white cartoons are modified from Conklin’s drawings [42]. **i–r** Images of embryos during late cleavage and early epiboly stages labeled with the actin cytoskeleton marker UTPH-GFP (*white*) and histone H2B-RFP (red); in some panels, the 4d clone is labeled with diI (red). *AV* animal pole view, *VV* ventral/vegetal pole view, *LV* lateral view. The 4d clone can often be identified without direct labeling because the UTPH-GFP is preferentially expressed or stabilized in this clone (e.g. as in **l, m, q, r**). Scale bar equals 50 μm. **s–w** Time lapse light sheet confocal images of an embryo expressing the microtubule-cytoskeleton bio-sensor EMTB-GFP and an RFP-membrane biosensor (MEM-RFP). Lateral view. Many cells are seen to divide over the course of the time-lapse (*arrow* mitotic spindle), but the micromere cap does not make a significant advance towards the vegetal pole during this period. See also Additional file 1, which corresponds to panels **s–w**

**Fig. 2:**
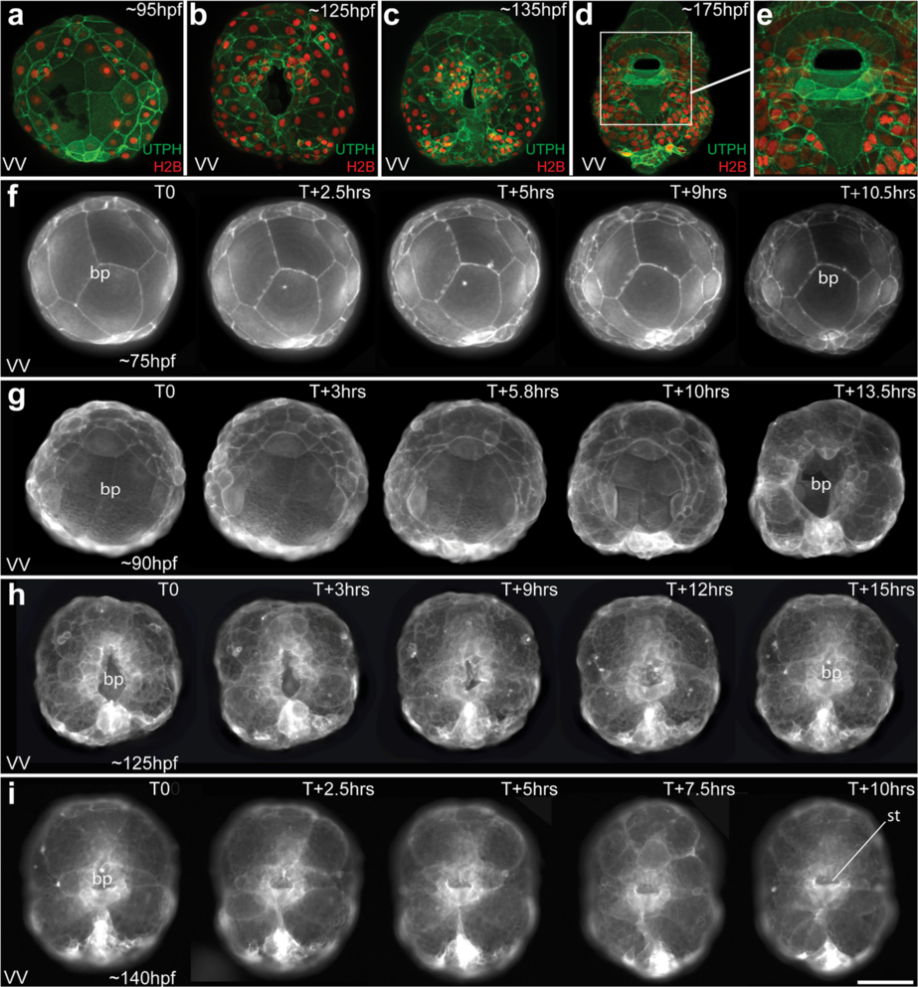
Narrowing of the blastopore during later epiboly, and formation of the mouth/stomodeum. **a–e** Confocal images of living embryos expressing UTPH-GFP to mark the actin cytoskeleton, and histone H2B-RFP to mark the nuclei. Ventral views are shown during mid epiboly (**a**), late epiboly (**b**), elongation (**c**), and mouth formation (**d–e**). **f–i** Time-lapse movies of embryos expressing UTPH-GFP during epiboly and mouth formation. *VV* ventral view. *Scale bar* equals 50 μm. See also Additional files 2, 3, 4, and 5, which correspond to panels **f, g, h,** and **i**

Specific cells were pressure microinjected with fluorescent lineage tracers, as previously detailed, to follow their contributions to specific germ layers, the blastopore, mouth, foregut, and anus (Rhodamine Green Dextran, cat # D-7153, or DiIC18 (3), cat # D-282, Life Technologies, Grand Island, NY; [31, 33–36]. In some cases, multiple cells were injected, and sub-lineages were followed, by sequential injection of two cells with these different tracers. All second and third quartet micromeres (Fig. 1a–h; Additional file 11: Figure S1) were individually microinjected to follow their behavior during the process of gastrulation (Figs. 2, Additional file 12: Figure S2; 3, Additional file 13: Figure S3; 4, Additional file 14: Figure S4; 5, Additional file 15: Figure S6; 6, Additional file 16: Figure S6; 7, Additional file 17: Figure S7; 8, Additional file 18: Figure S8; 9,Additional file 19: Figure S9; 10, Additional file 20: Figure S10; 11, Additional file 21: Figure S11; and 12, Additional file 22: Figure S12;) and their contributions to the formation of various germ tissues and the gut (Figs. 13, Additional file 23: Figure S13; 14, Additional file 24: Figure S14; and 15, Additional file 25: Figure S15). For each micromere, the two daughter cells (i.e., 2a^1^ and 2a^2^ or 3a^1^ and 3a^2^ cells) were also labeled independently (a total of 16 sub-lineages; Figs. 14 and 15). A minimum of five embryos were scored, in the live embryo, for each clone examined, and these were all found to be highly regular.

**Fig. 3:**
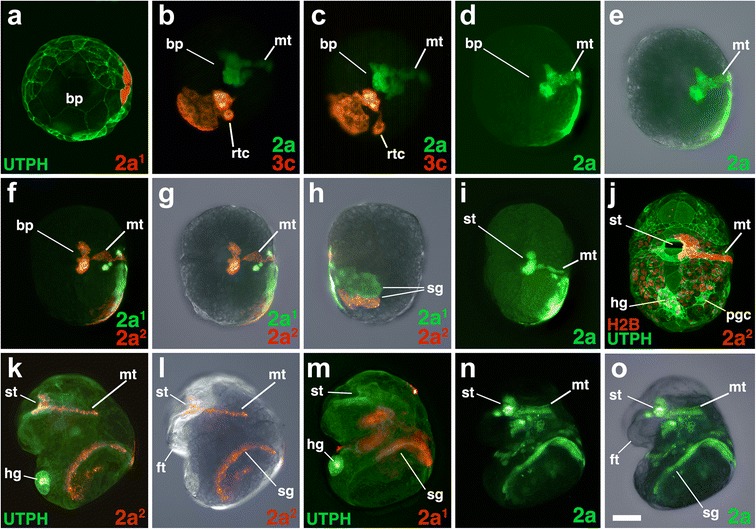
Fates of micromere 2a, and its subclones, during gastrulation and organogenesis. Images of live embryos with dextran- and diI-labeled 2a, or 2a subclones, as indicated. In some cases, the zygote was previously injected with mRNAs coding for fluorescent fusion proteins for histone H2B-RFP (H2B) and/or the actin-binding domain of utrophin-GFP (UTPH) to visualize nuclei or cell outlines, respectively, where indicated. Anterior is up in all cases. **a** Ventral view during early epiboly. Corresponding ventral-view images are shown in **b-c**, **d-e**, **f-g** during late epiboly with different combinations of fluorescence and/or DIC layers shown. **h** Shows a dorsal view of the same embryo shown in **f-g. i, j** Ventral views of older elongating embryos. **k, l** Corresponding right lateral views of an older embryo undergoing organogenesis. **m** Left lateral view of an older embryo undergoing organogenesis. **n, o** Corresponding left lateral view of an older embryo undergoing organogenesis. *bp* blastopore, *ft* foot, *hg* hindgut rudiment, *mt* metatroch, *pgc* primordial germ cell, *rtc* right terminal cell, *sg* shell gland, *st* stomodeum/mouth. *Scale bar* equals 50 μm

**Fig. 4:**
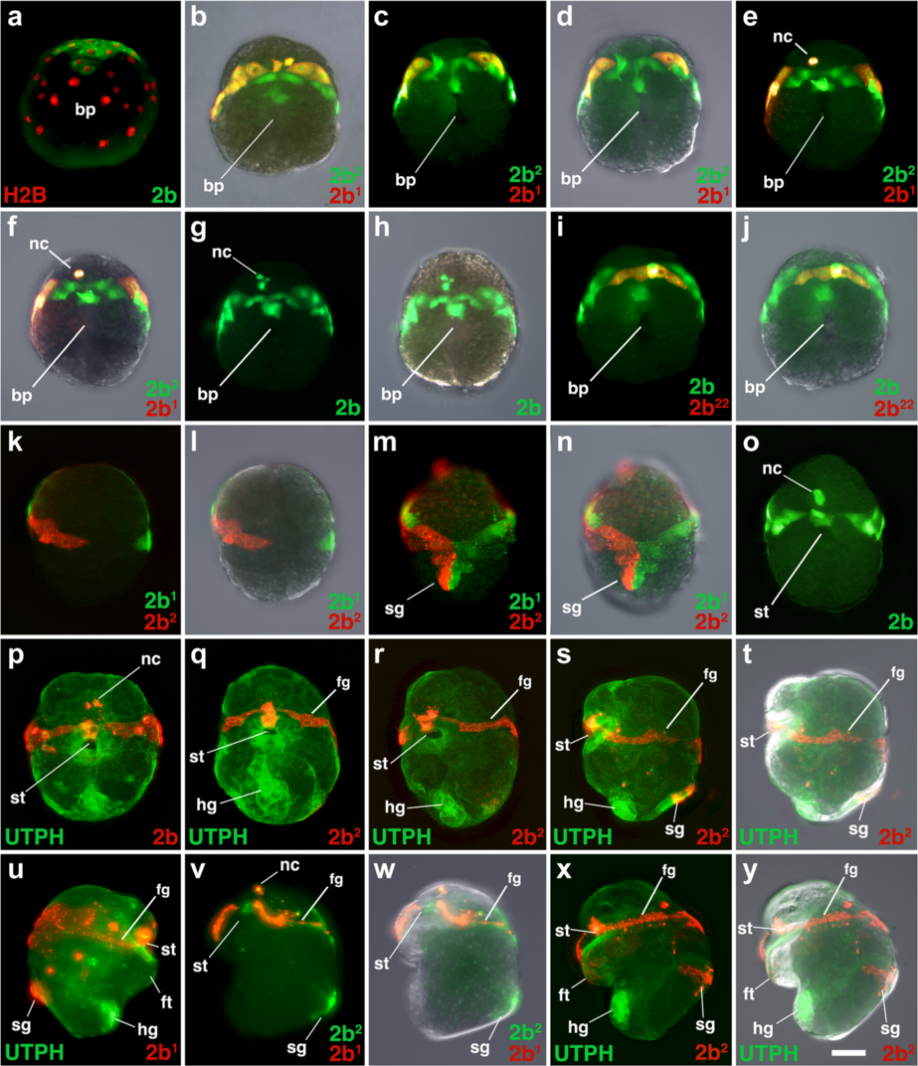
Fates of micromere 2b, and its subclones, during gastrulation and organogenesis. Images of live embryos, with dextran and diI-labeled 2b, or 2b subclones, as indicated. In some cases, the zygote was previously injected with mRNAs coding for fluorescent fusion proteins for histone H2B-RFP (H2B) and/or the actin-binding domain of utrophin-GFP (UTPH) to visualize nuclei or cell outlines, respectively, where indicated. Anterior is up in all cases. **a** Ventral view during an early stage of epiboly. **b** Ventral view during a later stage of epiboly. Corresponding ventral views are shown in **c-d**, **e-f**, **g-h**, **i-j** during epiboly with different combinations of fluorescence and/or DIC layers shown. Corresponding dorsal images are shown in **k-l**, **m-n** during epiboly. **o–q** Ventral views of older, elongated embryos. **r** Oblique left-lateral view of the ventral surface of an older embryo. Corresponding left-lateral images are depicted in **s-t** and **x-y** of older embryos undergoing organogenesis. **u** Right lateral view of an older embryo undergoing organogenesis. **v, w** Corresponding oblique left-lateral views of the ventral surface of an older embryo undergoing organogenesis. *fg* food groove, *nc* neural cells. Other labels are the same as those used in Fig. 3. *Scale bar* equals 50 μm

**Fig. 5:**
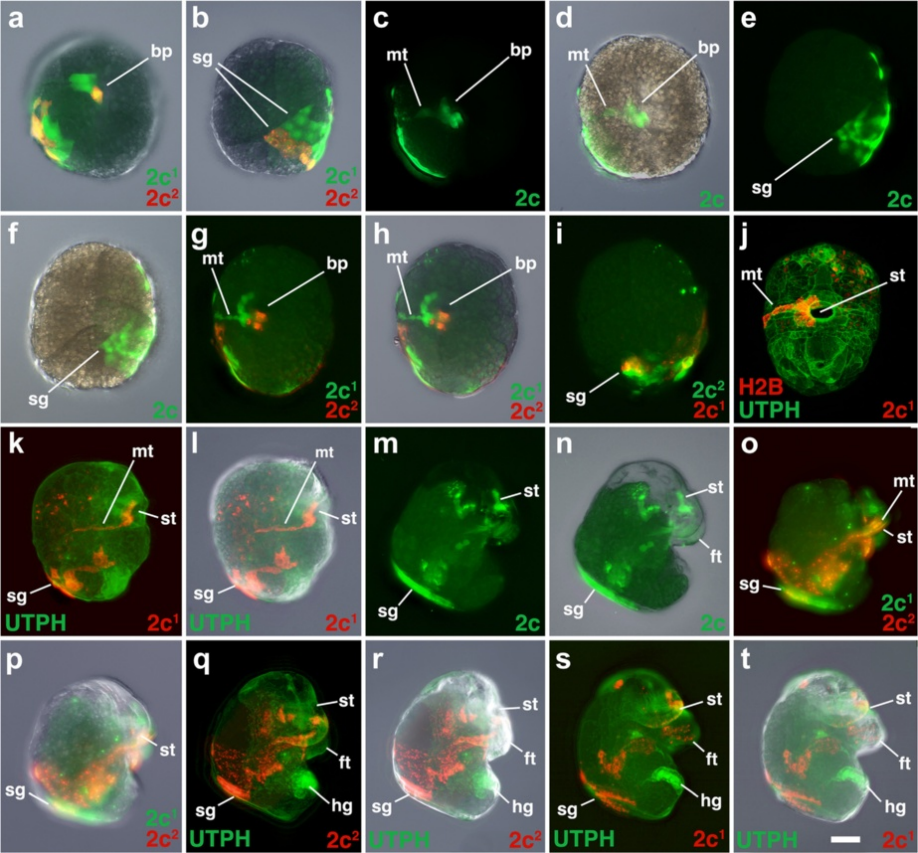
Fates of micromere 2c, and its subclones, during gastrulation and organogenesis. Images of live embryos with dextran and diI labeled 2c, or 2c subclones, as indicated. In some cases, the zygote was previously injected with mRNAs coding for fluorescent fusion proteins for histone H2B-RFP (H2B) and/or the actin-binding domain of utrophin-GFP (UTPH) to visualize nuclei or cell outlines, respectively, where indicated. Anterior is up in all cases. **a, b** Corresponding ventral and dorsal views of an embryo near the end of gastrulation with different combinations of fluorescence and/or DIC layers shown. **c, d** Corresponding ventral views of an embryo near the end of gastrulation. **e, f** Corresponding ventral views of a slightly older embryo at the end of gastrulation. **g, h** Corresponding ventral views of an older elongating embryo. **i** Dorsal view of an elongating embryo. **j** Ventral surface view of an embryo at the onset of organogenesis. **k, l** Corresponding right lateral views of an embryo at the onset of organogenesis. **m, n** Corresponding right lateral views of an older embryo during organogenesis. **o, p** Corresponding oblique dorsal view of an embryo during organogenesis. Corresponding right-lateral views of embryos undergoing organogenesis are shown in **q-r**, **s-t**. *tc* terminal cells. Other labels are the same as those used in Fig. 3. *Scale bar* equals 50 μm

**Fig. 6:**
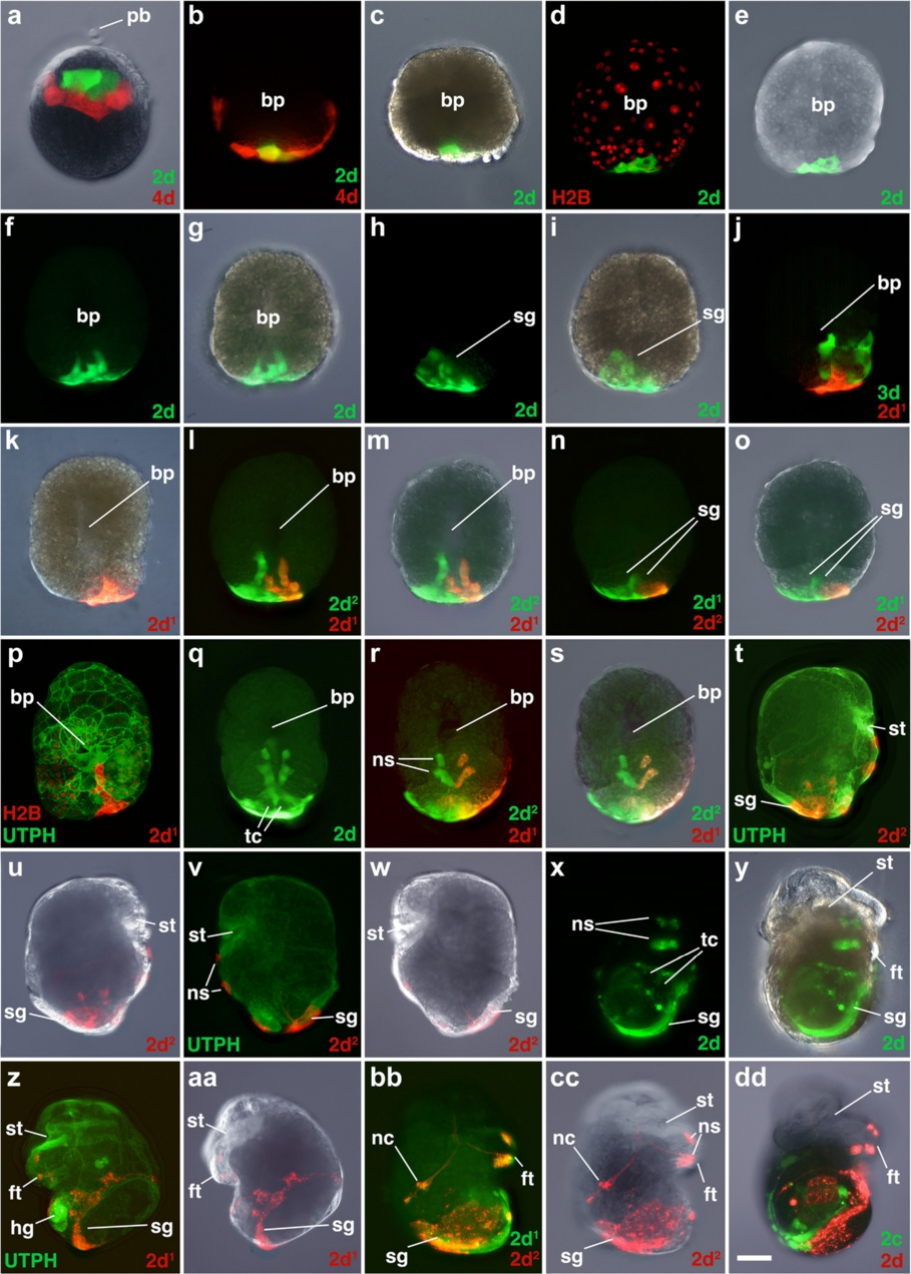
Fates of micromere 2d, and its subclones, during gastrulation and organogenesis. Images of live embryos, with dextran and diI-labeled 4d, 2d, or 2d subclones, as indicated. In some cases, the zygote was previously injected with mRNAs coding for fluorescent fusion proteins for histone H2B-RFP (H2B) and/or the actin-binding domain of utrophin-GFP (UTPH) to visualize nuclei or cell outlines, respectively, where indicated. Animal pole is up in **a**, anterior is up in **b–dd. a** Dorso-lateral view of an early epiboly-stage embryo. Corresponding ventral views of embryos during epiboly are shown in **b-c**, **d-e**, **f-g** with different combinations of fluorescence and/or DIC layers shown. **h, i** Corresponding dorsal views of same embryo shown in **f, g**. Corresponding ventral view images of successively older embryos undergoing epiboly are shown in **j-k**, **l-m. n, o** Shows corresponding dorsal views of an embryo at the same stage as that shown in **l, m. p** Ventral surface view of an elongated embryo. **q** Ventral view of embryo undergoing elongation. Note unlabeled voids where the two terminal cells (tc) from 3c^221^ and 3d^221^ reside. **r, s** Corresponding ventral views of embryo undergoing elongation. **t, u** Right-lateral views of older embryo at the onset of organogenesis. **v, w** Corresponding left-lateral views of embryo at the onset of organogenesis. **x, y** Ventral views of embryo undergoing elongation. Unlabeled voids occupied by the two terminal cells (*tc*) are also indicated in **x**. Corresponding left-lateral (**z, aa**) and right-lateral/oblique (**bb, cc**) views of older embryos during organogenesis. **dd** Shows a ventral view of an embryo during organogenesis. *pb* polar body, *ns* neurosensory cell. Other labels are the same as those used in Figs. 3 and 4. *Scale bar* equals 50 μm

**Fig. 7:**
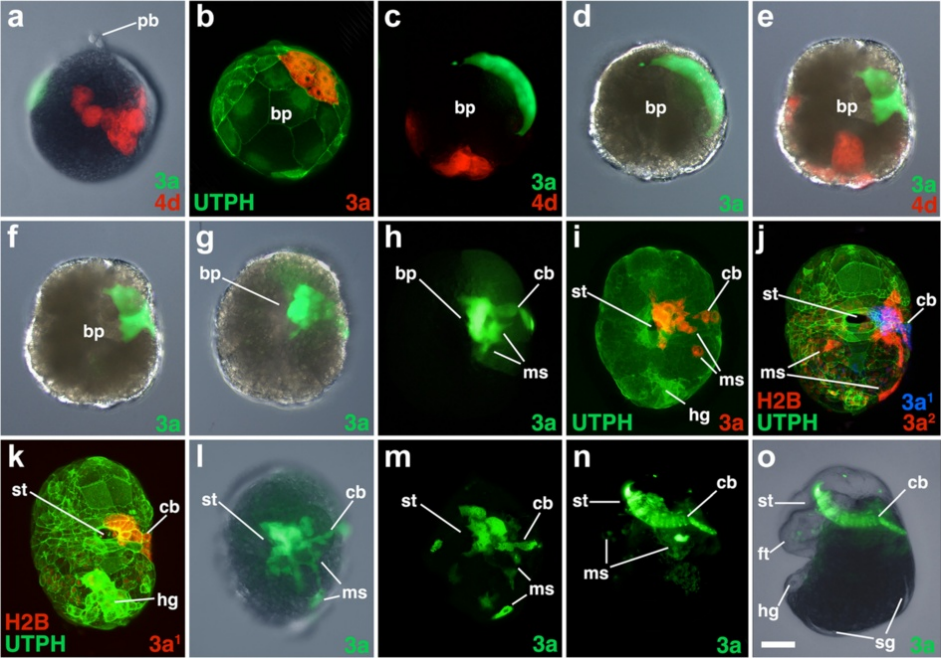
Fates of micromere 3a, and its subclones, during gastrulation and organogenesis. Images of live embryos, with dextran and diI-labeled 4d, 3a, or 3a subclones, as indicated. In some cases, the zygote was previously injected with mRNAs coding for fluorescent fusion proteins for histone H2B-RFP (H2B) and/or the actin-binding domain of utrophin-GFP (UTPH) to visualize nuclei or cell outlines, respectively, where indicated. Animal pole is up in **a**. Anterior is up in **b–o. a** Dorso-lateral view of an early epiboly-stage embryo. **b** Ventral (vegetal) view of early epiboly-stage embryo. **c-d**, **e-f** Show corresponding ventral views of early and mid epiboly-staged embryos, respectively, with different combinations of fluorescence and/or DIC layers shown. **g, h** Corresponding ventral views of later stage embryos undergoing epiboly. **i** Ventral view of an older, elongating embryo. **j, k** Ventral views of two embryos just prior to the onset of organogenesis. Note that for the original stack of confocal images shown as a projection in **j**, the 3a^1^ and 3a^2^ clones are spatially separated in the *Z* axis, making it possible to pseudocolor them separately, as labeled. Corresponding ventral views of embryo just prior to the onset of organogenesis are shown in **l, m, n, o** Left-lateral view of an embryo during organogenesis. *cb* ciliary band, *ms* mesenchyme. Other labels are the same as those used in Figs. 3 and 6. *Scale bar* equals 50μm

**Fig. 8:**
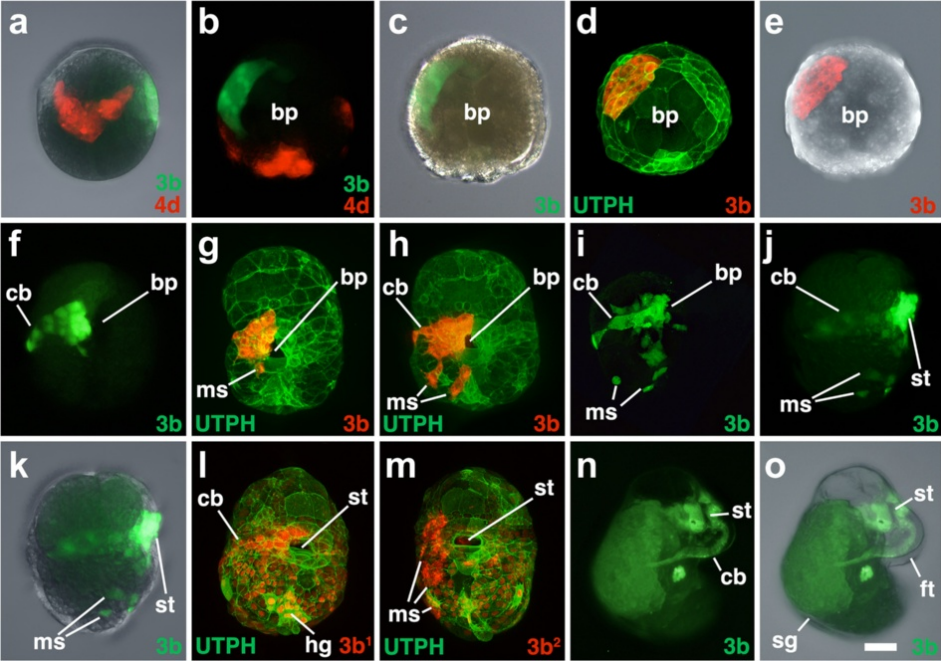
Fates of micromere 3b, and its subclones, during gastrulation and organogenesis. Images of live embryos, with dextran and diI-labeled 4d, 3b, or 3b subclones, as indicated. In some cases, the zygote was previously injected with mRNAs coding for fluorescent fusion proteins for histone H2B-RFP (H2B) and/or the actin-binding domain of utrophin-GFP (UTPH) to visualize nuclei or cell outlines, respectively, where indicated. Animal pole is up in **a**. Anterior is up in **b–o. a** Lateral-dorsal view of an early epiboly-stage embryo. **b-c**, **d-e** show corresponding ventral (vegetal) views of early epiboly-staged embryos with different combinations of fluorescence and/or DIC layers shown. **f** Ventral view of embryo during later epiboly. **g, h** Show ventral views of two stages of mesenchyme migration. **i** Ventral view showing numerous mesenchyme cells. **j, k** Corresponding right-lateral views of embryos during organogenesis. **l, m** Ventral surface views. **n, o** Corresponding right-lateral views of older embryos during organogenesis. Labels are the same as those used in Figs. 3, 6, and 7. *Scale bar* equals 50 μm

**Fig. 9:**
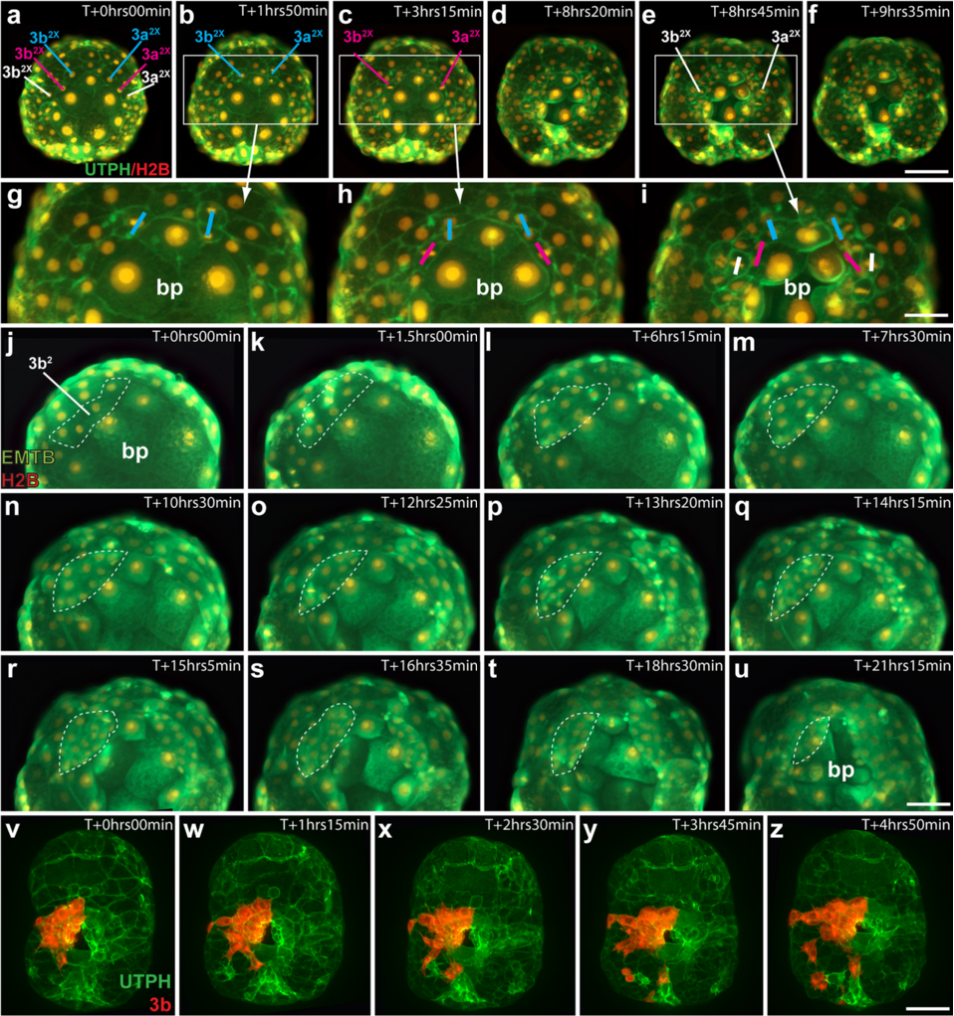
Behavior of ectomesoderm (3a^2^, 3b^2^). **a–i** Time-lapse movie of an embryo injected with utrophin-GFP (UTPH) to mark cell outlines, and histone H2B-RFP (H2B) to mark nuclei, where indicated. Ventral view. *bp* blastopore. Several unidentified (x) daughter cells of 3a^2^, 3b^2^ are marked, *in colors*, to show the orientation of cell division and position of ectomesodermal precursor cells during the narrowing of the anterior blastopore lip. *Scale bar* in **f** equals 50 μm; *scale bar* in **i** equals 25 μm. **j–u** Frames from a timelapse movie of an embryo injected with ensconsin-GFP (EMTB) to mark microtubules, and histone-RFP (H2B) to mark nuclei. Ventral view. *Dashed white lines* outline the 3b^2^ clone. *Scale bar* in **u** equals 30 μm. **v–z** Spinning disk confocal frames from a time-lapse of an embryo expressing utrophin-GFP (UTPH) globally, and in which the 3b micromere was labeled with diI (red). Ventral view. *Scale bar* in **z** equals 50 μm. See also Additional files 6, 7, and 8

**Fig. 10:**
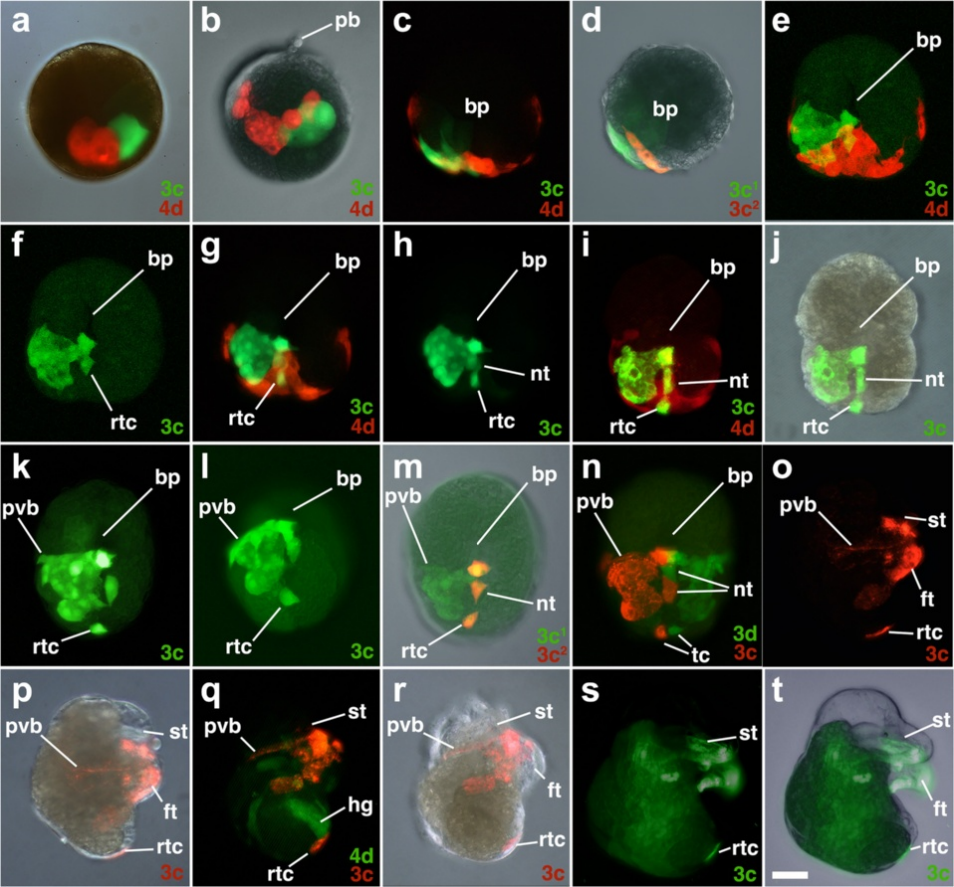
Fates of micromere 3c, and its subclones, during gastrulation and organogenesis. Images of live embryos, with dextran and diI-labeled 4d, 3c, or 3c subclones, as indicated. Animal pole is up in **a** and **b**. Anterior is up in **c–t. a, b** Dorso-lateral views of early epiboly-stage embryos. **c, d** Ventral views of early epiboly-stage embryos. Corresponding ventral views of late epiboly-stage embryos are shown in **e-f**, **g-h**, **i-j** at successive stages of development with different combinations of fluorescence and/or DIC layers shown. **k, l** Corresponding ventral and posterior views of an elongating embryo. **m, n** Ventral views of embryos during elongation. Corresponding right-lateral views of embryos during organogenesis **o-p**, **s-t**. Note *green* background fluorescence is higher in **s, t. (Q-R)** Corresponding oblique, ventro-lateral views of an embryo during organogenesis. *nt* neurotroch, *rtc* right terminal cell, *pvb* posterior velar band. All other labels are the same as those used in Figs. 3 and 6. *Scale bar* equals 50 μm

**Fig. 11:**
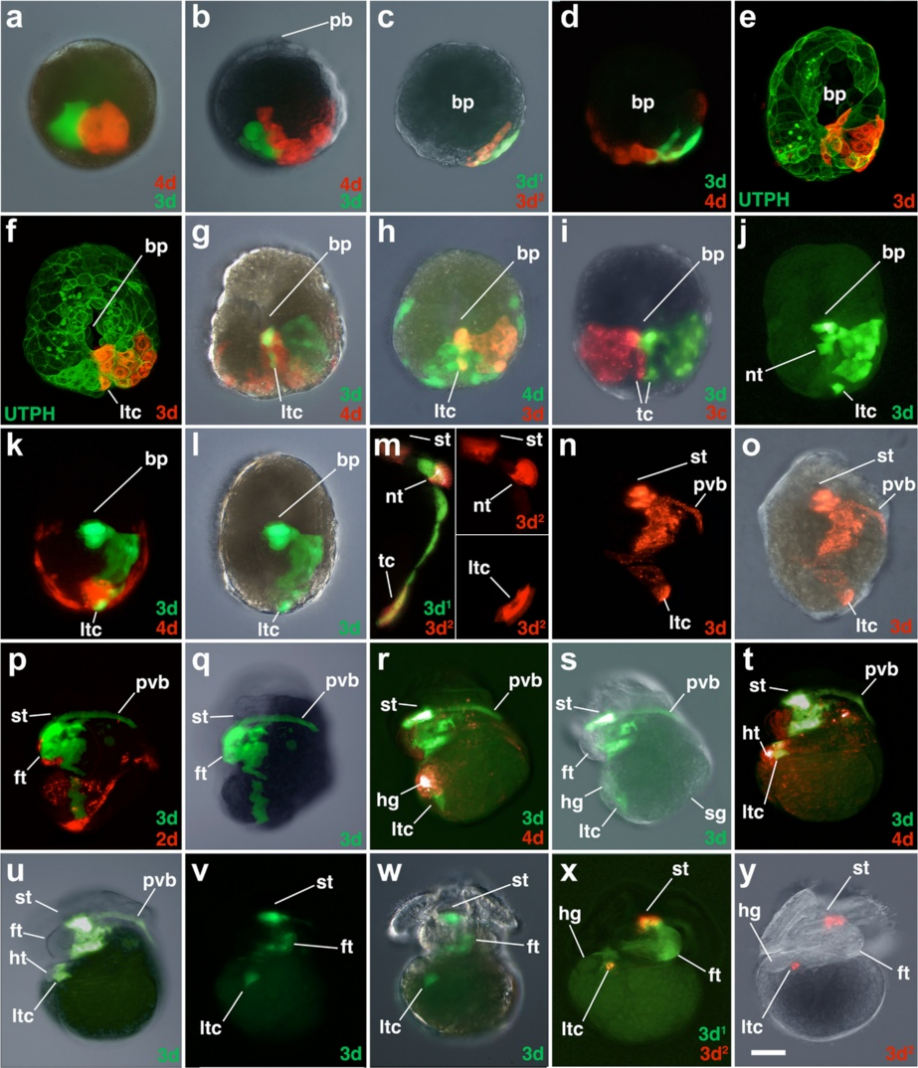
Fates of micromere 3d, and its subclones, during gastrulation and organogenesis. Images of live embryos, with dextran and diI-labeled 4d, 3d, or 3d subclones, as indicated. In some cases, the zygote was previously injected with mRNAs coding for fluorescent fusion proteins for the actin-binding domain of utrophin-GFP (UTPH) and histone H2B-RFP to visualize nuclei or cell outlines, respectively, where indicated. Animal pole is up in **a** and **b**. Anterior is up in **c–y. a, b** Dorso-lateral views of early epiboly-stage embryos. **c, d** Ventral views of early epiboly stage embryos. **e–j** Ventral views of elongating embryos later during epiboly. **k, l** Corresponding ventral views of an embryo during elongation with different combinations of fluorescence and/or DIC layers shown. **m** Right-lateral higher magnification views of an elongating embryo with inserts showing some of the ventral ciliated cells (shallow confocal stacks centered at the ventral midline). **n, o** Corresponding ventral views of embryos during organogenesis. Corresponding left lateral views of embryos during organogenesis **p-q**, **r-s**, **t-u**. Corresponding ventral views of early veliger stage embryos **v-w**, **x-y**. *ltc* left terminal cell. All other labels are the same as those used in Figs. 3, 6, and 10. *Scale bar* equals 50 μm for **a–l** and **n–y**. *Scale bar* equals 25 μm for **m** and 20 μm for its inserts

**Fig. 12:**
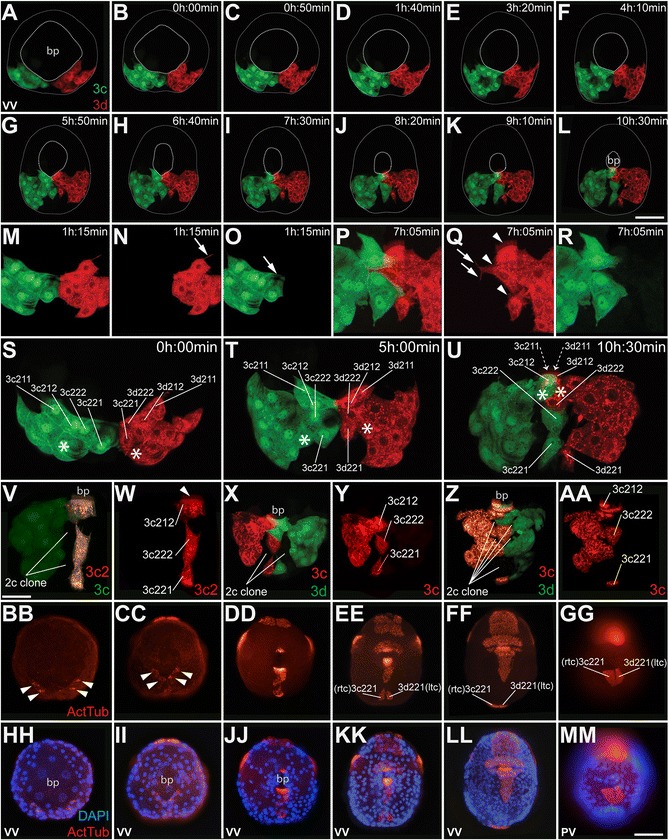
Behavior of posterior blastopore lip cells undergoing convergence and extension (3c^2^, 3d^2^). **a–aa** Projected confocal Z slices of embryos injected with dextran or diI, into 3c, 3d or their subclones, as indicated. **a** Ventral view of an embryo at epiboly stage. **b–u** Frames from a time-lapse of the same embryo as shown in **a. b–l** Frames from a time-lapse movie of the same embryo undergoing convergence and extension to zipper the posterior blastopore (*bp*) closed. **m–r** Frames from the same movie showing membrane protrusions (arrows) and cilia (arrow heads) on the cells undergoing convergent extension (zippering). **s, t** Frames from the same movie showing that the cells undergoing convergence and extension are from the 3c^2^ and 3d^2^ cells. The *asterisk* (*) marks cells from the 3c^1^ and 3d^1^ clones that migrate anteriorly towards the ventral side of the stomodeum. In **u**, the 3c^211^ and 3d^211^ cells have entered the deeper parts of the mouth and are out of view of the stack, indicated by *dashed lines*. **v–aa** Live confocal images of post-zippering-stage embryos showing the movement of the 3c^221^ and 3d^221^ cells posteriorly, between cells of the 2d clone. **bb–mm** Images of fixed embryos stained for acetylated tubulin (to mark cilia) and DAPI (to mark DNA). **bb, cc** Shows ventral views (**vv**) of embryos at the early stages of convergent extension and *arrowheads* point to cilia on cells at the posterior edge of the blastopore. **dd–ff** Ventral views of embryos during elongation. The ciliated left and right terminal cells (*ltc*, *rtc*) are labeled. **gg** Posterior view (*pv*) of the same embryo shown in **ff**. *Scale bar* in **l** and **mm** are equal to 50 μm; *scale bar* in **v** equals 20 μm. See also Additional files 9 and 10

**Fig. 13:**
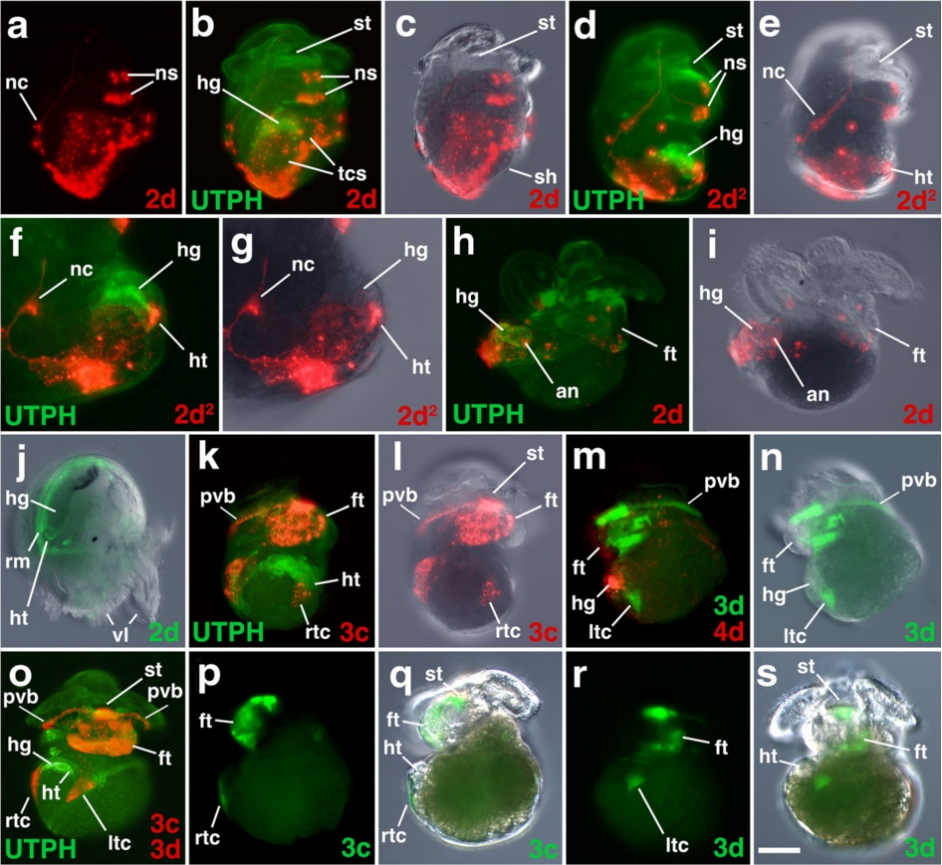
Origin of the anus (2d^2^). Images of live embryos, with dextran and diI-labeled 4d, 3d, 3c, 2d, or 2d subclones, as indicated. In some cases, the zygote was previously injected with mRNAs coding for fluorescent fusion protein for the actin-binding domain of utrophin-GFP (UTPH) to visualize cell outlines, where indicated. Anterior is up in all cases. **a–c** Corresponding ventral views of embryos during organogenesis with different combinations of fluorescence and/or DIC layers shown. **d, e** Corresponding right-lateral (oblique-ventral) views of embryo during organogenesis. **f, g** Corresponding higher magnification right-lateral views of the posterior end of an embryo during organogenesis. **h, i** Corresponding ventral views of veliger stage embryo after the anus as opened. **j** Ventral view of a veliger stage embryo just before the anus opens. Note that the hindgut is somewhat swollen. **k, l** Corresponding ventral views of pre-veliger stage embryos. **m, n** Corresponding (oblique-ventral) left-lateral views of the pre-veliger stage embryos. **o** Ventral view of early veliger. **p, q** Corresponding (oblique-ventral) left-lateral views of an early veliger. **r, s** Corresponding ventral views of an early veliger. *an* anus, *nc* neural cell, *ns* neurosensory cell, *rm* right mantle, *tcs* terminal cells (called *tc* in Fig. 6). All other labels are the same as those used in Figs. 3, 4, 10, and 11. *Scale bar* equals 50 μm for **a–e**, **h–s**. *Scale bar* equals 25 μm in **f, g**

**Fig. 14:**
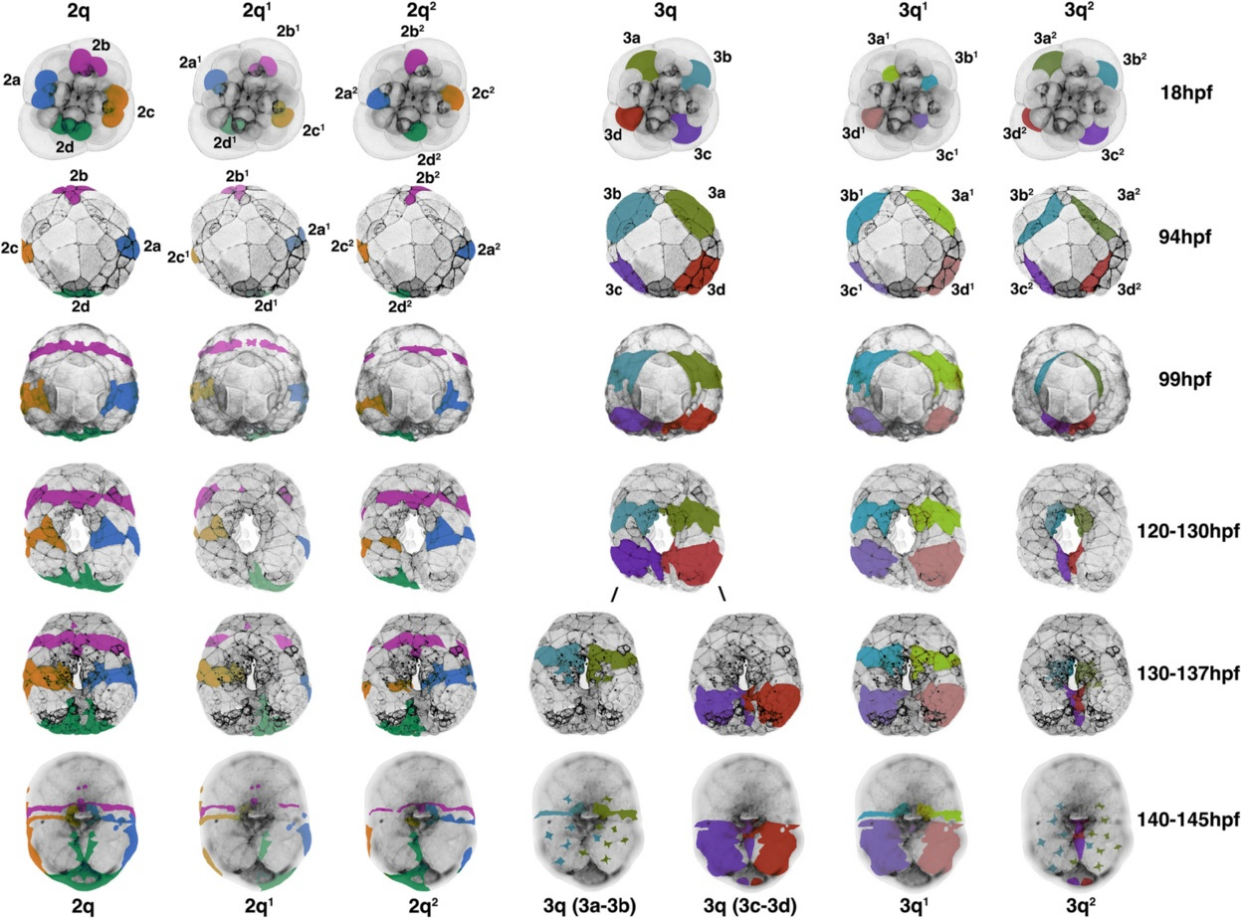
Summary of second and third quartet clones. *Columns* show clones, colored as labeled, and according to those shown in Figs. 1 and 15. *Rows* show time points as indicated at the far right. *Top row* shows animal views; all other rows show ventral views. The smaller, irregularly shaped, stippled cells of the 3a^2^ and 3b^2^ clones represent ecto-mesenchyme located below the ectoderm

**Fig. 15:**
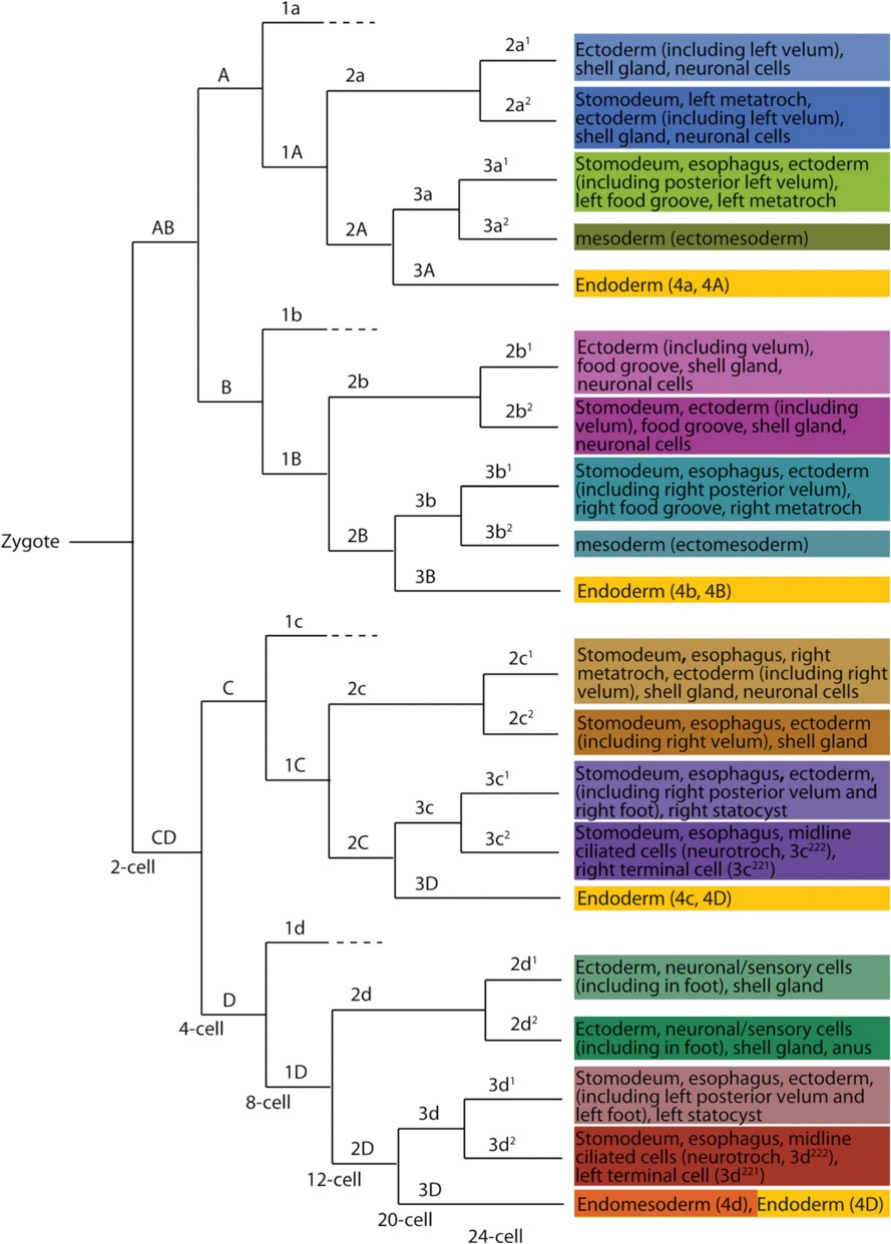
Lineage diagram of second and third quartet micromeres and third quartet macromeres. *Colors* correspond to those given in Figs. 1 and 14

### Fluorescently tagged protein expression

To help distinguish individual cells and their nuclei in live embryos, we microinjected synthetic mRNAs to express various combinations of fluorescently tagged proteins (Fig. 1). None of the constructs used contain *Crepidula*-specific sequence, yet they are expressed robustly, and fluorescence was detected within a few hours of injection. DNA in the nuclei was followed using histone H2B-RFP (H2B-RFP) and histone H2B-GFP (H2B-GFP) fusions, while plasma membrane was followed with an RFP-membrane fusion (MEM-GFP). These clones were the gift of the McClay lab, Duke University [37]. We followed live F-actin using a GFP fusion of the actin-binding domain of utrophin (UTPH-GFP) and live microtubules using GFP or RFP fusions of the MT binding domain of ensconsin (EMTB-3xGFP). These clones were the gift of the Bement Lab (University of Wisconsin) [38, 39] and are now available commercially (https://www.addgene.org/26737/; https://www.addgene.org/26741/). Synthetic mRNAs were generated from linearized plasmids using the mMessage mMachine kit (Life Technologies, Grand Island, NY) and purified with RNeasy MinElute Cleanup kit (Qiagen, Valencia, CA). Stock concentrations of these individual mRNAs ranged from 1 to 2 μg/μl in RNAse-free sterile dH_2_O. RNAs were diluted to a working concentration of 300–500 μg/μl. To visualize the solutions as they were being injected, the RNAs were mixed with a sterile filtered RNAse-free solution of 0.5 % phenol red, (1 part phenol red solution, cat. #P0292, Sigma, St Louis, MO, to 2 or 3 parts RNA solution). In this manner, a faint red cloud can be visualized inside the zygote during injection. Synthetic mRNAs were microinjected into fertilized eggs prior to first cleavage and approximately 5–10 % of the cell’s volume was injected. When the fertilized eggs are first collected, it is not possible to know exactly when first cleavage will take place. If the injections are carried out too soon before first cleavage, some graded/mosaic level of expression may be noted at later stages. The latter cases were not used for further study. Visible levels of expression can first be detected under a fluorescence dissecting scope around the four- to eight-cell stages.

### Fixation and histology

Embryos and larvae were fixed in a 3.7 % solution of ultrapure formaldehyde (made using the manufacturer’s 10 % stock solution, Ted Pella, Inc., Redding, CA, USA) dissolved in FSW with added Instant Ocean Aquarium Sea Salt Mixture (United Pet Group, Blacksburg, VA, USA) to adjust for the reduced osmolarity (0.47 gm added per 50 ml of final working volume). Embryos were fixed for 1 h at room temperature and rinsed three times in sterile 1× PBS (1× PBS:1.86mM NaH_2_PO_4_, 8.41mM Na_2_HPO_4_, 175mN NaCl, pH 7.4), followed by three washes in 100 % methanol before storage at −80 °C. Acetylated tubulin antibody labeling (1:400, cat #T7451, Sigma, St. Louis) followed the method described in [40]. A solution of 0.5 μg/ml DAPI (Life Technologies, Grand Island, NY) dissolved in 1× PBS was applied to embryos following antibody labeling to visualize nuclei. Embryos were incubated in the dark for 10 min, followed by three washes in 1× PBS/0.1 % Tween, and stored in 80 % glycerin/20 % 1× PBS at 4 °C until imaging.

### Microscopy

Live embryos expressing fusion proteins, or labeled with traditional lineage tracers (diI or dextrans), were mounted in filtered seawater between Rain-X-coated (ITW Global Brands, Houston, TX) glass slides and coverslips supported by tiny clay feet (Van Aken Plastalina, Rancho Cucamonga, CA, USA). For long-term time-lapse imaging, the coverslips were sealed with molten VALAP to prevent desiccation [41]. For time-lapse, embryos were imaged every 5–10 min for anywhere from 4 to 48 h. Widefield images were captured with a Zeiss Axio Imager 2 (Carl Zeiss Inc., Munich, Germany). Scanning confocal imaging was carried out with an inverted Zeiss LSM 700, Zeiss LSM 780, and Zeiss Cell Observer spinning disc microscopes (Carl Zeiss Inc., Munich, Germany). Light sheet imaging was carried out with a Zeiss Light Sheet.Z1 (Carl Zeiss Inc., Munich, Germany). For image processing, Z-stacks were prepared to make maximum projections with Fiji and ImageJ software (National Institutes of Health, Bethesda, MD, USA) or the focus stacking software Helicon Focus (Helicon Soft Ltd., Kharkov, Ukraine).

Fixed embryos labeled with antibodies were placed on Rain-X-coated (ITW Global Brands, Houston, TX) glass slides in 80 % glycerol/20 % 1× PBS. Coverslips were first prepared with small supporting feet made from plastic “Tough Spots” adhesive labels (Diversified Biotech, Boston, MA, USA). These adhesive labels were trimmed into 1–2-mm squares, stacked three layers thick, and adhered to the four corners of glass coverslips to prevent the embryos from being crushed. Specimens were visualized on a Zeiss Axioplan microscope (Carl Zeiss Inc., Munich, Germany), and imaging was conducted with a Spot Flex camera (Spot Imaging Solutions, Sterling Heights, Michigan). Image stacks were combined and flattened using Helicon Focus (see above).

## Results

### Behavior of cells during cleavage and early epiboly

Early development in *Crepidula* has been described by Conklin [42] and Hejnol et al*.* [31] (see also [30]). The fertilized egg undergoes two rounds of orthogonal, equal cleavage occurring parallel to the animal-vegetal axis, giving rise to four blastomeres (macromeres) founding embryonic quadrants A, B, C, and D. These macromeres undergo several rounds of highly asymmetric cleavages that give rise to four tiers of smaller micromere quartets (“q”) at the animal pole: 1a–1d (1q), 2a–2d (2q), 3a–3d (3q), and 4a–4d (4q) (Fig. 1a–h; Table 1). As each micromere quartet is born, the sister quartets’ vegetal macromeres (“Q”) are renamed: 1A–1D (1Q); 2A–2D (2Q); 3A–3D (3Q), and 4A–4D (4Q). The embryo is radially symmetric up through the early 24-cell-stage (when the first three quartets have been born). Symmetry is broken at approximately 27 h past fertilization (hpf) at 20 °C, when the 3D macromere divides precociously, giving rise to the 4d micromere at the 25-cell stage (Fig. 1a). At approximately 33 hpf, 4d divides bilaterally to form left and right teloblasts (ML and MR) which produce endoderm and mesoderm (“endomesoderm,” Fig. 1b).

**Table 1:** *Crepidula fornicata* staging system

**Process**	**Description/Landmarks** ***(Stage)***	**Age (hr)**	**Examples**
**Cleavage**	**Formation of the four micromere quartets**	**0-48hr**	
	* Zygote to 25-cell stages*	0-27hr	Fig. 1a, e, i
	Formation of the first three micromere quartets and 4d		
	Compaction, *Round stage*	40-47hr	Figs. 1n, 10a, 11a
	* Fourth Quartet formation*	47-48hr	not shown
	Formation of 4a-4c		
**Epiboly**	**Coverage of macromeres by the animal cap micromeres** *(% Epiboly)*	**48-140hr**	
	*50-55 % Epiboly, Round Stage*	60hr	Figs. 1c, g, k-w, 7a, 10b, 11b
	*65 % Epiboly*	91hr	Figs. 1d, h, 2a, 3a, 6b-e, 7b-d, 8b-e, 9j, 10c-d, 11c-d
	*Flattened Round stage*		
	Compressed along the AV axis		
	Convergent-Extension occurring and 2d progeny have been excluded from the blastopore	97-140hr	Figs. 2b-c, g-h, 6f-o, 11e-i, 12a-aa
	Ectomesoderm enters the blastopore	110-130hr	Figs. 7g, 9p-u
	Ectomesoderm begins to disperse	130hr	Figs. 7h, 8g, 9v
**Elongation**	**Embryo undergoes elongation along the anterior-posterior axis**	**117-169hr**	
	*Square* or *Rectangular stage*	117hr	Figs. 2h, 3b-e, 7e-f
	* Short* or *early ovoid stage*	120 hr	Figs. 2i, 3f-h, 4b-l, 5a-f, 6k-o, 7g-h, 9v-z, 10e-h
	Blastopore still central		
	Invaginated shell gland is present	137hr	Figs. 3i, 4p-q, 5j-l, 12kk-mm
	*Long* or *late ovoid stage*	140hr	Figs. 3j, 5j-l, 6q-s, 7j-k, 10i-n
	Mouth displaced anteriorly		
**Organogenesis**	**Appearance of external morphological rudiments, including: velar lobes, foot, shell**	**170-227hr**	
	* Early Organogenesis*	170hr	Figs. 4r-w, 6t-w, 11n-o
	Flattened shell plate		
	*Late Organogenesis*	196hr	Figs. 3k-o, 4x-y, 5m-t, 6x-dd, 7n-o, 8n-o, 10o-t, 11p-s, 13a-g, k-n
	Embryo, shell have a pronounced convex dorsal curvature		
**Larval Development**	**Pre-hatching veliger larvae have prominant ciliated velar lobes, a foot, operculum, pigmented ocelli, curved external shell enclosing the visceral mass, protruding absorptive cells, and beating heart**	**228hr-4 weeks+**	
	*Young Veliger Larva*	228hr	Figs. 11t-y, 13j, o-s
	Anus opens	12 days	Figs. 13h-i

The bolded headings list the principle phases of development, while their substages are listed below in unbolded text

A fate map of these teloblasts has recently been established up through their fifth division [34]. Thus, the well-defined cleavage pattern of the 4d lineage can be used to quickly orient the embryo during early, spherical or “round” stages of gastrulation (Fig. 1; Table 1). We found that some fluorescent biosensors, particularly actin (Fig. 1q) and membrane sensors (Fig. 1t), appear brightest in the 4d lineage and thus can be used to assess the age and orientation of embryos, even in the absence of specific cells being directly labeled. The 4d micromere is born as a tear-shaped cell with its pointed end directed towards the center of the embryo, which is covered by the micromere cap (Fig. 1a–i). The rounded end is initially exposed at the surface of the embryo, as it is not yet covered fully by the micromere cap. All of its mesodermal derivatives, and some of its endodermal derivatives, will be born from the more pointed, internal end, and thus those cells are internalized at birth (Fig. 1k–q). In contrast, the larger rounded, exposed area of the 4d cell is where the future 1mL/R and 3mL/R endodermal lineages will be born (Fig. 1c). At first, the micromere cap does not fully cover these cells, but they will be covered during epiboly (99 hpf, Fig. 1c–d and o–q; Table 1). At approximately 47 hpf, the 3A–3C macromeres divide to form the 4a–4c micromeres (Table 1). Following this period of early cleavage divisions, the embryo undergoes compaction and assumes a tight spherical shape (Fig. 1k, p, r; Table 1). The micromere-derived animal cap gradually expands to cover more surface area (Fig. 1q–w), and by 60 hpf, the animal cap covers 50–55 % of the spherical surface having reached its widest circumference at the equator of the embryo (Fig. 1r; Table 1).

### Cellular behavior during gastrulation: epiboly of the micromere cap

While we do not fully understand the mechanisms that regulate expansion of the micromere cap, it likely involves continued cell divisions (Fig. 1s–w) and thinning of micromere cap progeny. As the animal cap expands, it covers more of the endomesodermal lineages (Figs. 1 and 2). At 91 hpf, the spherical embryo begins to change shape as gastrulation continues to take place by the process of epiboly. At this time, the embryo begins to flatten noticeably along the animal-vegetal axis (Fig. 1h, p; Table 1). The edge of the cap (derived from the second and third quartet micromeres, the colored lineages in Fig. 1a–h) can just begin to be observed when viewed from the flattened vegetal pole, which can be seen in Fig. 2f. At this stage, the animal cap covers approximately 65 % of the surface of the embryo. Time-lapse movies of embryos expressing fluorescent membrane (MEM-RFP) or actin cytoskeleton (UTPH-GFP) biosensors did not show obvious lamellipodial or filopodial extensions during animal cap expansion (see Fig. 1s–w; Fig. 2f, and Additional files 1 and 2). This initial expansion of the micromere cap correlates with cell divisions in the epithelium (Fig. 1s–w), which generates an increasingly larger number of smaller cells, and correlates with the flattening or thinning of this layer of cells (e.g., Fig. 1i, k, n, r, o, q). After reaching the equator, the micromere cap begins to constrict towards the vegetal pole (Fig. 2), advancing over the 4Q macromeres and 4q micromeres for the next 50 h as the blastopore narrows (Fig. 2a–b and  f–h; Table 1). During this process, the embryos become irregularly shaped as the internal macromeres and fourth quartet micromeres begin to undergo divisions (beginning at approximately 99 hpf; Fig. 2b, g, h). At this time, the four macromeres divide equally, and Conklin [42] referred to this as the formation of a “fifth quartet.” Divisions are oriented such that the internal fourth quartet macromeres and micromeres ultimately assume a rectangular packing arrangement. The embryo then elongates, which begins by 117 hpf, and the embryo assumes a flattened rectangular shape (Fig. 2b, h; Additional files 3 and 4; Table 1). Throughout these stages, a noticeable depression, which becomes the embryonic gut or archenteron, can be seen at the site of the blastopore. By 120 hpf, the embryo has assumed a more rounded, short ellipsoidal form, with the elongated axis corresponding to the future anterior-posterior axis (Fig. 2c, h–i).

The blastopore begins to be displaced towards the anterior end of the embryo by 141 hpf (Fig. 2d–e, i; Additional file 5). Most of the elongation appears to occur in the post-trochal region, and as a consequence, the blastopore is displaced in an anterior direction (Table 1). Though Conklin [42] reported that the blastopore temporarily closes deep within its recess, we have not observed this. Our observations indicate that an external opening persists throughout the process of gastrulation, and during this process, the macromeres can be seen within it. This opening ultimately becomes the mouth (Fig. 2d–e). By elongation stages, gastrulation has been completed and the process of organogenesis begins to unfold (Table 1). Overt signs of organogenesis can be seen by 170 hpf, when the rudiments of the velar lobes, foot, and shell gland can be clearly seen (Figs. 3, 4, 5, 6, 7, 8, 9, 10, 11, 12, and 13). After several more weeks, advanced veliger larvae hatch and enter the water column where they begin to feed and ultimately settle to undergo metamorphosis to form juvenile snails.

### The fates of cells around the blastopore

Although a fate map was previously generated for each cell up through the 25-cell stage [31], those fates were related only to the tissues present within the veliger larvae. That preliminary work established the basic germ layer derivatives of those embryonic cells, but did not describe the behavior of lineages during gastrulation. In particular, we were interested in where these clones were situated relative to the blastopore. Thus, we labeled individual second and third quartet micromeres, and some of their progeny, and observed their behavior during gastrulation. We also followed their contributions to specific tissues, including the ectomesoderm and ectodermal components of the digestive tract, such as the mouth, esophagus, and the anus (Figs. 3, 4, 5, 6, 7, 8, 9, 10, 11, 12, 13, 14, and 15).

Labeling individual clones was necessary to understand how cells behave near the blastopore. Lineage tracing results are described below for each of the 2q and 3q micromeres, as well as their immediate progeny 2q^1^/2q^2^ and 3q^1^/3q^2^, representing a total of 16 clones (Figs. 3, 4, 5, 6, 7, 8, 9, 10, 11, 12, 13, 14, and 15). As specified by Conklin [42], the daughters situated in a clockwise position around the animal-vegetal axis (as viewed from the animal pole), or those located closer to the animal pole, carry the superscript 1 (e.g., 2a^1^). Those situated in a counter-clockwise location, or situated closer to the vegetal pole, carry the superscript 2 (e.g., 2a^2^). Individual clones are shown in Figs. 3, 4, 5, 6, 7, 8, 9, 10, 11, 12, and 13 for different developmental stages described in Table 1. A schematic diagram summarizing the relationships of these clones for six different stages of development between 18 and 145 hpf is also shown in Fig. 14. A lineage diagram showing the ultimate fate of these clones is also shown in Fig. 15. Time-lapse movies were also prepared to capture the dynamic behavior of certain cells (Additional files 1, 2, 3, 4, 5, 6, 7, 8, 9, and 10). These analyses influenced how we defined the blastopore in *Crepidula.*

**Fig. 16:**
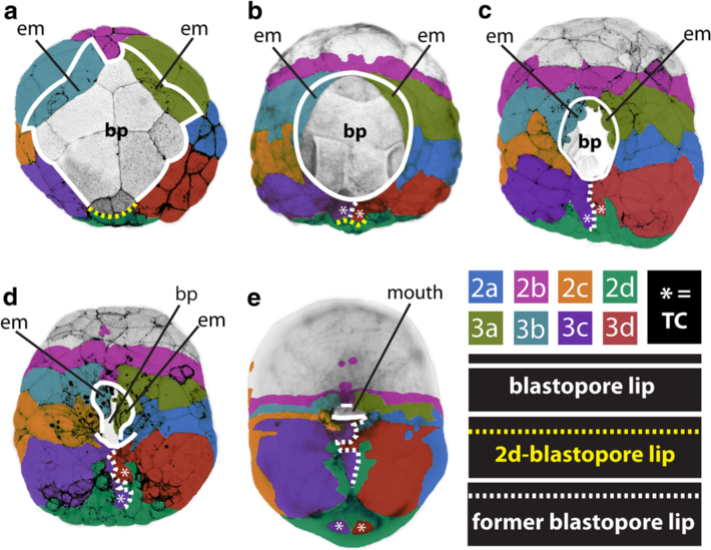
Morphogenesis of the blastopore lip. **a–e** Vegetal/ventral views during gastrulation with the future anterior at the top of the figure. Time points are the same as those shown in rows 2–6 in Figs. 1 and 14: **a** ~94 hpf, **b** ~99 hpf, **c** ~120–130 hpf, **d** ~130–137 hpf, **e** ~140–145hpf. The *coloring* shows the relative clonal contributions of cells within the embryo and to the blastopore lip. The blastopore lip is marked in each panel by a *solid white line*. The blastopore lip marks the boundary between the ectodermal micromere cap and the endoderm/endomesoderm/ectomesoderm. As gastrulation proceeds, some cells leave the blastopore lip, but remain on the surface, and these are marked by a *dashed white line*. The 2d cells are marked by a *dashed yellow line* in **a, b**, but are more difficult to follow in time points **c–e** and thus are not shown. The 3c^2^- and 3d^2^-derived terminal cells (TC) are marked with an *asterisk* to follow their migration after they leave the blastopore lip. Some cells of the blastopore lip move internally into the blastocoel/archenteron, and the *solid white line* can no longer be seen in some areas **d, e**. *bp* blastopore, *em* ectomesoderm (derived from 3a^2^ and 3b^2^)

Definitions of “blastopore” can vary between animals, referring alternatively to the literal hole into which endoderm/mesoderm move, or to the endodermal and mesodermal cells themselves, or to cells surrounding the hole [2, 10, 13, 25]. To avoid confusion and enable direct comparisons with other species, we distinguish between the blastopore (the hole) and the cells surrounding that hole—which we call the blastopore *lip* (Fig. 16, Additional file 26: Figure S16). These definitions became necessary because of the nature of epiboly, since gastrulation begins when the ectodermal cap spreads over the macromeres, but it is not until later stages that the endodermal/mesodermal tissues themselves re-arrange to form an archenteron and a literal hole (Fig. 16; Table 1). Additionally, even after an obvious blastopore hole forms, the cells surrounding it change through cell rearrangement.

In *Crepidula*, during early stages of gastrulation, the blastopore consists of endodermal/endomesodermal precursors derived from the fourth quartet macromeres (4A-4D) and fourth quartet micromeres (4a-4cd) (Fig. 16a–b); at later stages of gastrulation the blastopore is an actual hole/cavity leading inside of the embryo, and is surrounded by cells of the developing archenteron (Fig. 16c–e). The blastopore *lip* is made up of progeny derived from each of the second and third quartet micromeres that give rise to ectoderm exclusively (Figs. 14 and 16, dashed white line). This boundary between ectoderm and endoderm was defined through our detailed lineage analysis during gastrulation stages. For example, initially, we assumed that the edge of the micromere cap (colored cells in Fig. 1d) was exactly equivalent to the blastopore lip, but lineage tracing of the 3a and 3b cells showed that the 3a^2^ and 3b^2^ sublineages make mesoderm (Figs. 14 and 15), and so we had to adjust our delineation of the blastopore lip to run between the 3a^2^/3a^1^ and 3b^2^/3b^1^ clones (compare Figs. 1d and 16a).

### The second quartet (2a and 2c)

All four of the second quartet micromeres give rise to progeny that lie along the lip of the blastopore (Fig. 14). After their first division, specific daughters of these cells mainly lie along the blastopore lip (2a^1^, 2b^2^, 2c^2^, and 2d^1^ and 2d^2^ cells; Figs. 3a, 5a, and 14). 2a and 2c give rise to bilaterally symmetrical clones of ectodermal cells that extend to the left and right sides of the embryo, respectively (Figs. 3b–j and 5c–j). For the most part, their daughters, 2a^2^ and 2c^1^, make mirror symmetrical contributions to the development of the mouth (Figs. 3j, 5j, 14, and 15). Both of these cells also extend a long thin line of cells from the developing blastopore/mouth in a bilaterally symmetrical fashion, which generate the left and right secondary ciliary band (i.e., the metatroch; Figs. 3 and 5). These cells also form much larger separated posterior, dorsolateral clones of cells located in the post-trochal region that also contribute to the shell gland/mantle (from both 2a^1^, 2a^2^ and 2c^1^, 2c^2^; Figs. 3h, k and 5i, q). On the other hand, 2a^1^ and 2c^2^ make some slightly different contributions. Though both of these cells make contributions to the posterior, dorsolateral clones of cells located in the post-trochal region (Figs. 3h, m and 5i, s), 2a^1^ does not contribute to the mouth or esophagus (Fig. 3m), while 2c^2^ does (Fig. 5k). Fine speckles of labeled material are also seen mainly on the right anterior side of the head, which are derived from the progeny of 2c (Fig. 5c–e, g, h–l, o–p). These appear to be too small to be cells, and their identity is uncertain (see also [18]).

Hejnol et al. [31] reported that ectoderm covering the left external kidney (absorptive cell) was derived from 2a; however, Lyons et al. [34] showed that this structure is derived from 4d and is directly exposed to the external environment. Hejnol et al. [31] also reported that some ectomesoderm is also derived from 2c. Specifically, they reported that the ectodermal covering of the larval heart and muscles of the heart are derived from 2c. Here, we find no evidence of any ectomesoderm being derived from 2c (Fig. 15). In fact, recently, we showed that the heart muscles are actually derived as endomesoderm from 4d [34]. Hejnol et al*.* [31] reported that 2a and 2c give rise to the left and right coverings of the statocysts, respectively, and to the pedal and apical ganglia, as well as the osphradium (the latter being derived from 2c, which we have not verified here).

### The second quartet (2b)

Initially, 2b gives rise to a clone of ectodermal cells located along the very anterior edge of the blastopore (Figs. 4a and 14). This clone gives rise to two wings, and later gives rise to bands that extend bilaterally to the sides of the embryo (Fig. 4a–j). The right and left bands of cells are derived mainly from 2b^1^ and 2b^2^, respectively. A single ciliated cell derived from 2b^2^ lies at the ventral midline at the very anterior (top) opening of the mouth (Fig. 4o–s). An isolated group of 2–3 small rounded cells derived from 2b^1^ also lie in the head anterior to the mouth (Fig. 4o–p, u–w). These cells presumably give rise to neural cells or may undergo cell death, as they become hard to identify in older embryos. 2b gives rise to ectoderm located anterior to the mouth. The bilaterally symmetrical bands of cells derived from 2b^1^ and 2b^2^ give rise to the ciliated food grooves (see also [31]), as well as some nerves in the head (Figs. 4q–y and 15). As these bilateral bands extend further to the dorsal side, (Fig. 4k, l), they eventually fuse along the dorsal midline (Fig. 4m, n). Here, two large cells each derived from 2b^1^ and 2b^2^ detach from their sister cells and move posteriorly along the dorsal surface to give rise to part of the shell gland and mantle. As this occurs, those former cells appear to become depressed below the surface within the ectoderm compared to the surrounding cells. The bands of cells derived from 2b^1^ and 2b^2^ also expand to contribute to dorsal-lateral ectoderm adjacent to the head bulge (head vesicle, which is derived from the first quartet micromeres, not followed here). The dorsal-right lateral clone derived mainly from 2b^1^ expands to a greater extent, compared to that on the dorsal-left side (mainly from 2b^2^, Fig. 4t vs. u).

### The second quartet (2d)

The ectodermal progeny of 2d originally lie along the very posterior edge of the blastopore (Figs. 6a–b and 14), but this is the only clone of second quartet cells that is eventually excluded from the blastopore lip and does not contribute to either the esophagus or the mouth (Figs. 6b–p and 15). These cells are pushed away from the lip when clones derived from 3c and 3d, specifically 3c^2^ and 3d^2^, fuse along the ventral midline at approximately 97 hpf (Figs. 6j, 10, 11, 12, and 14). That behavior is described in greater detail below. 2d^1^ and 2d^2^ give rise to nearly bilaterally symmetrical clones of posterior ectoderm (Fig. 6l–m, q–s). Both of these cells give rise to much of the post-trochal ectoderm and both contribute also to the formation of the shell gland and the mantle (Figs. 6t–dd and 14). There are two unlabeled voids inside the posterior clone of labeled 2d cells that represent the two “terminal cells” derived from 3c^2^ and 3d^2^, respectively (Figs. 6q and 13b; see further discussion below). As cells derived from 3c^2^ and 3d^2^ extend posteriorly into these clones (together with cells derived from 3c^1^ and 3d^1^), they cleave them to form two “V” shaped arms, one each derived from 2d^1^ and 2d^2^, respectively (Fig. 6q–s). These two arms each contain three cells that ultimately form bilateral, neuro-sensory structures located in the foot (Fig. 6v–dd; see description by [31]). 2d also gives rise to prominent neurons that extend from the post-trochal region to those neuro-sensory cells that become located in the tip of the foot, as well as to the apical organ in the head. A prominent pair of bilateral ganglia are located on the left and right sides of the post-trochal region from which these axons extend (Fig. 6bb, cc). The tip of the developing hindgut intestine is located directly under posterior ectoderm derived from 2d (Figs. 6 and 13a–j). Often, there is a fluorescently brighter patch of labeled ectoderm directly over the terminal end of the hindgut that is derived from 2d^2^ (Fig. 13b, d, f–g). As asymmetric rotation of the visceral mass takes place, this patch of ectoderm follows the posterior end of the digestive tract and moves up the right side of the developing veliger larva (Fig. 13a–c, h–j). The proctodeum or anus opens within this 2d^2^ derived ectoderm later in development at approximately 12 days of development (Figs. 13h–j and 15).

### The third quartet (3a and 3b)

All four of the third quartet micromeres generate cells located along the lip of the blastopore (Figs. 3, 4, 5, 6, 7, 8, 9, 10, 11, 12, 13, and 14). Together, these contribute to most of the circumference of the blastopore. 3a and 3b give rise to bilaterally symmetrical clones that reside on the left and right sides of the embryo, respectively, which are situated closer to the future anterior pole (Figs. 7b–f and 8b–e). Their vegetal most progeny, derived from 3a^2^ and 3b^2^ are the ones that initially lie along the anterior-lateral rim of the blastopore (Fig. 14). These cells ultimately dive deep during gastrulation and form the ectomesoderm (Figs. 7h–o, 8f, h–k, m, n, and are described in greater detail below, and Fig. 9). As this happens, the animal progeny derived from 3a^1^ and 3b^1^, in turn take their place along the rim of the blastopore lip/blastopore boundary and contribute to the development of the esophagus and the mouth (Figs. 7j, k and 8l). Cells arrayed in short straight bands extend to the left and right sides of the 3a^1^ and 3b^1^ clones away from the blastopore/mouth (Figs. 7h–p and 8g–j, l). These arms extend only as far as the lateral edges of the embryo. These clones contribute to the food groove and metatroch and post-trochal (post-oral) velar ectoderm in the vicinity of the mouth and the foot (Figs. 7o, p and 8n, o).

### Formation and behavior of ectomesoderm (3a^2^ and 3b^2^)

The lineage analyses described above revealed that ectomesoderm arises from the 3a^2^ and 3b^2^ micromeres. These cells initially divide twice to form a line of four cells located along the anterior-lateral edges of the blastopore, at the boundary with the blastopore lip (Figs. 9a–i, 14, 16a). Ultimately, these cells undergo further proliferation to give rise to a larger number of mesenchymal cells (Figs. 7, 8, and 9j–u). As gastrulation takes place, these cells roll into the blastopore to occupy deeper positions (Fig. 9s–u; Additional files 6 and 7). As this begins to take place, the anterior blastopore narrows (Figs. 2 and 8f), and these cells appear to loosen contacts with neighboring cells and become more rounded (Fig. 9t, u). Eventually, they are covered by cells derived from their sister clones (3a^1^ and 3b^1^), which are part of the ectodermal lip of the blastopore (Figs. 9t, u and 14). The 3a^2^ and 3b^2^ progeny are internalized by a process of ingression and ultimately undergo epithelial-mesenchymal transition (EMT). They begin to migrate to remote locations within the embryo and larva beginning around 142 hpf (Fig. 9v–z; Additional file 8). Some of the progeny derived from the left 3a^2^ and right 3b^2^ micromeres cross the ventral midline during their migration (Figs. 7j, n and 8i). These cells form various fates, including velar muscles, muscles of the foot, as well as some fibers in the main retractor muscles, the latter being derived primarily from 4d (see [31]).

### The third quartet (3c and 3d)

3c and 3d give rise to bilaterally symmetrical (right and left) clones located along the posterior lip of the blastopore (Figs. 10, 11, 12, and 14). These cells contribute to the mouth, esophagus, foot, and post-trochal region (Fig. 15). The vegetal progeny, derived from 3c^2^ and 3d^2^, initially lie along the rim of the blastopore lip (Figs. 10d, 11d, and 12a, s). These cells each give rise to four progeny that form a single line of cells along the lip that do not divide further (Figs. 12 and 14). The medial ends of these two lines of cells are initially separated from one another by cells derived from 2d^1^ and 2d^2^ (Figs. 6, 14 and 16). Subsequently, 3c^2^ and 3d^2^ progeny move toward the ventral midline by a process of convergence and extension, and undergo intercalation, which is described in greater detail in the next section (Fig. 12). These cells all become multi-ciliated and form a group of cells that extends along the ventral midline, from the posterior esophagus and mouth, to the posterior end of the embryo (Fig. 12dd, ee).

The much larger group of cells derived from 3c^1^ and 3d^1^ contribute mainly to the left and right halves of the foot and to the statocysts (Figs. 10m–t, 11m, p–x, and 15). In addition, these cells give rise to thin bands that lie within the post-trochal ectoderm on the posterior surface of the right and left velar lobes (Figs. 10p–r, 11n–u, and 13k–o). Hejnol et al. [31] indicated that 3c and 3d give rise to the left and right external larval kidneys, but Lyons et al*.* [34] showed that both the left and right external larval kidneys (absorptive cells) are derived from 4d.

### The posterior blastopore lip: closure by convergence and extension

At flattened round stages, the posterior/lateral lip of the blastopore is made up of two lines of four cells each derived from the vegetal-most daughters of the 3c and 3d (i.e., 3c^2^ and 3d^2^; Fig. 12a, s). Initially, derivatives of 2d^1^ and 2d^2^ cells lie between these 3c^2^ and 3d^2^ cells at the lip (Figs. 6j, 12v, x, z, and 14). At approximately 97 hpf, 2d^1^ and 2d^2^ are pushed away from the posterior lip when the 3c^2^ and 3d^2^ progeny subsequently interdigitate by zippering along the ventral midline (Figs. 12a–u and 14; Additional file 9). This zippering is accomplished by a novel form of convergence and extension. A consequence of this zippering process is that the posterior blastopore lip closes, narrowing the blastopore.

Beginning at the time, these cells undergo zippering; they all become multi-ciliated (Fig. 12bb–mm; Additional file 10). Each of these four 3c^2^ and 3d^2^ progeny extend a long thin filopodial process along the edge of the blastopore lip that forms contacts at the posterior ventral midline (Fig. 12m–o). Those cells subsequently converge along the ventral midline to form two columns of cells that undergo intercalation and extension. The four originally medial cells (3c^221^, 3c^222^, 3d^221^, 3d^222^) are displaced posteriorly and removed from the blastopore lip (Figs. 10f–j and 11e–j). The four originally more lateral cells (3c^211^, 3c^212^, 3d^211^, 3d^212^) contribute to the mouth and esophagus (Figs. 10o–t, 11m–y, and 15), where the two most lateral cells end up diving deep into the archenteron to contribute to the esophagus (3c^211^, 3d^211^; Figs. 10i–o, 11m, and 12s–ll). Those next to them contribute to the posterior mouth (3c^212^, 3d^212^). The other cells form a line that extends along the ventral midline. Two of these lie just posterior to the opening of the mouth (3c^222^, 3d^222^). The two cells (3c^221,^ 3d^221^) that were initially the most medial cells prior to zippering give rise to the bilateral right and left “anal” cells, respectively, which were described by Conklin [42]. Despite their name, these two cells do not contribute to the proctodeum and to avoid further confusion we have renamed them “terminal cells” (Fig. 15; [43]; see discussion).

As the 3c^2^/3d^2^-derived cells zipper, they displace 2d progeny further away from the blastopore lip and towards the posterior pole of the embryo. They extend into the clone of cells derived from 2d, so that after zippering has finished, two elongated V shaped left and right columns of cells extend from the main 2d clone (the right arm from 2d^2^ and the left arm from 2d^1^; Figs. 6q–s, 12z–aa, and 14). The terminal cells initially lie within the clone of 2d ectoderm that sits over the terminal end of the hindgut (Figs. 10p–t and 11r–y). Subsequently, the terminal cells are displaced further from the end of the hindgut (Fig. 13k–s).

## Discussion

### Early epiboly: expansion of the micromere cap

Gastrulation occurs by diverse mechanisms among spiralians [10]. Epiboly is common to many species with yolk-rich eggs, including *Crepidula* [10, 42]. Though there are gross morphological descriptions of this process, very few specifically focus on the behavior of individual cell lineages [16, 18]. Here, we traced the lineages of the second and third quartet micromeres to specific germ layers, and to openings of the digestive tract (Figs. 1, 14, and 15).

In *Crepidula*, epiboly occurs as cells of the micromere cap proliferate and flatten to cover endodermal and mesodermal precursors (Figs. 1 and 2). These events are similar to what was described for *Helobdella* [16] and for the flatworms *Imogine mcgrathi* and *Maritigrella crozieri* [44, 45]. However, a double layer of ectodermal cells does not form in *Crepidula*, as described in *Maritigrella* [44]. In *Crepidula*, cell divisions occur continuously during epiboly, throughout the epithelium, whereas in *Helobdella*, mitoses in the cap are largely restricted to early stages of epiboly, and cell division is rarely observed at the leading edge of the cap [16]. To cover the large, yolky macromeres, the micromere cap’s marginal circumference must first increase to reach the equator and then decrease as the cap narrows around the vegetal hemisphere of the embryo. This latter step is accomplished in *Helobdella*, in part, by virtue of that fact that cells at the leading edge become wedge shaped, with their narrow edges at the lip. This wedging behavior was not pronounced in *Crepidula*; however, we noticed extensive cell shape changes at the leading edge (Fig. 2). These changes are discussed further below, for each of the second and third quartet micromeres, which make up the leading edge of the epithelial cap.

### Origin and behavior of the ectomesoderm (progeny of 3a^2^ and 3b^2^)

Spiralians typically have two sources of mesoderm. The first is *endo*mesoderm, derived from the 4d micromere, and gives rise to mesodermal derivatives such as adult muscle, heart, and excretory structures [34, 46]. Spiralian endomesoderm appears to be highly conserved with endomesoderm in other metazoans, both in terms of cell fates [34, 46] and gene expression [47, 19, 23]. The second is *ecto*mesoderm, which arises from various second and third quartet cells, depending on the species examined [10, 48]. Ectomesoderm gives rise to scattered mesenchyme, which can contribute to larval tissues such as the velar muscles in *Crepidula* [10, 31, 48, 49]. Ectomesoderm is likely a spiralian innovation, and the behavior and profile of genes expressed in these cells are not well understood [10, 50, 51]. An earlier study in *Crepidula* followed the contributions of embryonic cells later into larval development and reported that ectomesoderm arises from 3a, 3b, and 2c [31]. The origin of ectomesoderm from 3a and 3b is considered to be a plesiomorphic character among spiralians [10, 31]. In the present study, we observed no evidence of ectomesoderm arising from 2c (though this may arise at later stages of development). We found that ectomesoderm arises more specifically from the 3a^2^ and 3b^2^ cells (Figs. 7 and 8).

The mechanisms of ectomesoderm internalization have not been investigated in detail, but internalization is generally assumed to occur by an epithelial to mesenchymal transition (EMT) [10]. We show that clones of cells derived from 3a^2^ and 3b^2^ occupy the anterior-lateral sides of the blastopore by ~94 hpf when the lip has just passed the equator of the embryo (Table 1; Figs. 9 and 14). Initially, these cells are rather large and flat, forming lines of stretched cells along the anterior-lateral rim of the blastopore. Subsequently, these cells round up and proliferate (Fig. 9). Their progeny remain well rounded (which may indicate that they have weaker adhesive properties) while they collectively sink into the archenteron (Figs. 7, 8, and 9). As this occurs, these cells are covered by trailing cells derived from 3a^1^ and 3b^1^. Despite using various lineage tracers and fluorescently tagged biosensors for the actin cytoskeleton (utrophin-GFP), we did not observe any obvious signs of dynamic cell membrane protrusions (e.g*.*, filopodia or lamellipodia), or evidence of apical constriction in these cells during the initial phase of their entering the archenteron. Thus, the early behavior of these cells is distinct when compared to classic forms of EMT, such as in sea urchin primary mesenchyme ingression, where ingressing cells exit the cell cycle, extend filopodia, and slip out of the epithelium [52]. Only after they become covered by the surface ectoderm and move into the archenteron do these ectomesodermal cells extend filopodia and disperse, crawling to distant locations within the embryo (Fig. 9; Additional file 8).

### Origin and formation of the mouth (second and third quartet)

As ectomesoderm leaves the leading edge of the micromere cap, its circumference becomes reduced. This removal of cells may help drive constriction of the blastopore lip along its anterior-lateral edges, as the blastopore takes on a narrowed, slit-like appearance (Fig. 2). Likewise, other cells, derived from 2a, 2c and 3a–3d, also extend into the archenteron to form tissues of the foregut/esophagus, and this could also contribute to narrowing of the blastopore. Despite Conklin’s [42] statement, the blastopore does not completely close in *Crepidula*, and gives rise to the mouth in a true protostome fashion (Fig. 14), just as was recently reported for *Ilyanassa* [18]. Previous descriptions by Conklin [42] and Hejnol et al. [31] reported that the progeny of mainly the second quartet form the mouth in *Crepidula*, while third quartet progeny make the ectodermal esophagus (or foregut). By tracing the exact locations of these cells throughout gastrulation, we found that contributions to the mouth and esophagus are more complicated. The mouth is derived from 2a^2^, 2b^2^, 2c^1^, 2c^2^, 3a^1^, 3b^1^, 3c^1^, 3c^2^, 3d^1^, and 3d^2^, and all those except 2a^2^ and 2b^2^ also contribute to deeper tissues of the esophagus (Figs. 3, 4, 5, 6, 7, 8, 9, 10, 11, 12, and 15). These contributions are similar to those noted for *Ilyanassa* [18], including the small contribution of the 2b lineage (2b^2^) to the anterior-most part of the mouth. Interestingly, the relative contribution of second versus third quartet lineages to the mouth and esophagus appears to vary across species (see discussions in [53] and [18]). It could be that differential proliferation and cell re-arrangements during blastopore narrowing, along with different contributions of these cells to ectomesoderm, accounts for species-specific variation.

### Convergence and extension of the posterior blastopore lip: “zippering” of 3c^2^ and 3d^2^ progeny

By the time the blastopore lip has passed the equator, the posterior half of the blastopore lip is occupied by four cells derived from 3d^2^ on the left and four cells from 3c^2^ on the right (Figs. 1, 12, 14, 16). Between them, at the very posterior-most edge of the lip, lie two cells derived from 2d^1^ and 2d^2^. Beginning at ~94 hpf, these two cells are excluded from the blastopore lip by convergence and extension that involves intercalation of some of the 3c^2^ and 3d^2^ descendants. During this process, these cells extend thin processes that make contact with cells from the opposite sides. This convergence begins with the most medial, posterior cells in each clone (3c^221^, 3d^221^), followed by more lateral, anterior cells, which is reminiscent of a closing zipper. As these cells converge, they form a pair of columns along the anterior-posterior axis that undergo intercalation to form a more extended column of ciliated cells (Fig. 12dd, ee).

This study presents the first description of blastopore lip morphogenesis using clonal analysis and live imaging. Closure of the posterior blastopore has been described in other species (e.g., *Platynereis* [23], *Hydroides*, [17], *Patella*, [43]), but the specific cell behaviors involved were not investigated. The behavior exhibited by *Crepidula* likely takes place in other spiralians. Although Chan and Lambert [18] do not specifically remark on this phenomenon, their images of the 3c and 3d clones show that *Ilyanassa* likely develops in a similar fashion (their Fig. 4j, n). Among annelids, Woltereck’s [54] drawings of *Polygordius*, reveal a similar posterior-ventral “zig-zag” seam comprised of two columns of left and right cells, which might reflect a similar zippering behavior [7, 12, 54]. The process observed in *Crepidula* could also account for the arrangement of cells observed in the study of Lartillot et al. [43], in the gastropod *Patella* (see their Fig. 5). Those authors speculate that cells posterior to the blastopore elongate along the ventral midline by directed stem cell like divisions, but that was based solely on gene expression data and not on intracellular lineage tracing; it is possible that the elongation is instead the result of convergence and extension along with cell intercalation. Convergent extension was recently reported in the annelid *Platynereis* [55]; however, that process is different, as it occurs within two separate populations of neuroectodermal precursors that lie to the sides of the ventral midline after blastopore closure has taken place. Lineage tracing in each of these species would address how similar their gastrulation processes are to that found in *Crepidula*.

### Ciliary bands: the food groove and the metatroch (second and third quartet)

Hejnol et al. [31] reported that the metatroch, a ciliated, reverse-current (downstream) feeding band, is derived from 2a and 2c, while the ciliated food groove, lying between the metatroch and the prototroch (derived from the first quartet micromeres), is derived from 2b (Fig. 4). Here, we extend those results by showing that the food groove is derived from both 2b^1^ and 2b^2^, in addition to 3a^1^ and 3b^1^, and the metatroch is derived from 2a^2^ and 2c^2^, as well as 3a^1^ and 3b^1^. The 3c^1^ and 3d^1^ clones also extend long thin bands of cells, but those are located on the posterior surface of the velum, behind the metatroch. The differences between our studies may be related to the fact that Hejnol et al*.* [31] examined fixed embryos, and we analyzed live material, which is much better for making fine assessments of cell lineage fates. Gharbiah et al. [56] reported in *Ilyanassa* that the metatroch arises from 3a–3d and 2a–2c, and the food groove is from 2a–2c, as well as 3a–3b. These findings continue to support the argument that the homologization of these ciliated bands across large evolutionary distances is problematic [57].

### The neurotroch (3c^222^, 3c^222^)

Following the zippering of the posterior blastopore lip, the daughter cells from 3d^2^ and 3c^2^ each contribute to four sets of ciliated cells present in the posterior ventral ectoderm (Fig. 12). These include two deeper cells in the esophagus (3c^211^, 3d^211^), two in the mouth (3c^212^, 3d^212^), two cells we believe could be analogous to a neurotroch (3c^222^, 3d^222^), and more posteriorly, two “terminal cells” that could be analogous to a telotroch (3c^221^, 3d^221^). These cells all become ciliated during posterior blastopore closure (Fig. 12bb–mm).

In some larvae of other spiralians, the neurotroch (also sometimes called the gastrotroch, but not to be confused with the ciliated bands on the segments of polychaetes; see [58]) represents a double row of multi-ciliated cells that runs along the ventral midline, posterior to the mouth and extends towards the anus [59]. The neurotroch has been argued to be a plesiomorphic feature of spiralian larvae [60–62]. Cilia of the neurotroch beat towards the anus, but the exact function of the neurotroch is uncertain. This structure, which is present in some annelids and entoprocts, is apparently not found in platyhelminthes, nemerteans, rotifers, or molluscs [60, 62]. The neurotroch typically comes from the 2d lineage [29]. For example, in *Capitella*, the neurotroch is made up primarily of 2d cells, with 3c and 3d also contributing a small number of anterior cells [53]. In *Ilyanassa*, there are a few ciliated cells in the foot [56] derived from 3c and 3d [18]. We argue that the 3c^222^ and 3d^222^ cells of *Crepidula* could be analogous, or even homologous, to the neurotroch in other spiralians.

### The telotroch (3c^221^ and 3d^221^)

The telotroch (sometimes referred to as paratroch) represents a posterior ciliated band that encircles the anus and is believed to have a function in locomotion [63]. Telotrochs have been found in many annelids (including echiurans and sipunculids) and some molluscs, including the gastropod *Patella* [53, 64–66]. Its presence has also been reported for aplacophorans (e.g., *Neominiomorpha* and *Chaetodermorpha*), and given their basal position in the Mollusca, this structure is regarded as a plesiomorphic condition for that phylum [60, 67]. A telotroch has also been reported for the pilidium larva of one nemertean [68, 69], though this is likely a derived structure. Nielsen [60] argues that the telotroch is a feature of the ancestral trochophore larva, though Strathmann [63] suggests this structure could have evolved on multiple occasions, and Rouse [67] argues that only the prototroch is ancestral for the Trochozoa. The telotroch is said to be derived from 2d in the annelids *Arenicola* [70] and *Amphitrite* ([29, 71]). The telotroch in the polychaete annelid *Capitella* is also derived from 2d [53, 65]. In the gastropod *Patella*, the telotroch is mainly derived from 2d, but includes two distinctive anterior ciliated cells derived from 3c and 3d [64]. We argue that the two posterior ciliated cells in *Crepidula* (3c^221^ and 3d^221^) are homologous to those two telotroch cells. These represent the posterior-most cells in the *Crepidula* embryo following posterior blastopore lip closure (Fig. 12s–u). They are surrounded by ectoderm derived from 2d, and they are the only ciliated cells in this region (Figs. 6q, x and 12ff, gg). These cells represent what Conklin [42] and others referred to as “anal cells,” as they assumed that they contributed to the proctodeum. We show clearly that these cells do not make the anus, which arises instead from the 2d lineage (2d^2^, see below). Thus, we deemed it necessary to re-name these cells, and propose calling them “terminal cells”; the term used for what we assume to be the homologous cells in *Patella* [43]. Lartillot et al. [43] stated that terminal cells arise from the 3c^11^ and 3d^11^, and that these cells give rise to the anus, but there is no published lineage data to support those claims.

### Origin and formation of the anus (2d^2^)

There are various reports regarding the origins of the ectodermal anus (proctodeum) in spiralians [10, 53]. In the classical literature, for instance, Treadwell [72] argued that cells derived from 3c give rise to part of the proctodeum in *Podarke*, but Nielsen [29] suggests that this is not well demonstrated. The anus was said to be derived from 2d in the annelids *Arenicola* [70] and *Amphitrite* (Nielsen [29]). More recently, intracellular lineage tracing in *Capitella* [53] showed that the ectodermal anus is derived from 4d, which appears to be a unique situation. As mentioned above, earlier investigators referred to ciliated “anal cells”, which were thought to give rise to the anus (e.g., [42, 73]). We show in *Crepidula* that these terminal cells (derived from 3c^221^ and 3d^221^) are located near the end of the hindgut early during development, but they become displaced from this location at later stages (Fig. 13k–s). Instead, our current study demonstrates that the anus is derived from the 2d^2^ clone, which forms ectoderm lying directly over the termination of the hindgut (Fig. 13a–j). In *Capitella*, the 2d clone also surrounds the 4d-derived anus, and the 3c and 3d micromeres contribute cells to this area as well, but are subsurface, as 3c and 3d progeny are ectomesodermal in this species [53].

In a previous study [34], we observed that the anus did not form when specific progeny of 4d were ablated (e.g., 3mL/R), which contributes largely to the formation of the terminal endoderm of the hindgut. These data suggested that inductive interactions may be required to induce the formation of the proctodeum. It is possible, however, that we did not follow those larvae long enough to fully determine if the anus could form or not. Its development could have also been delayed in those experimental cases. In the course of this study, we found that the opening of the anus does not appear until 12 days after fertilization. On the other hand, the location of the anus may indeed require inductive interactions from the hindgut, as suggested by those earlier experiments. When performing the lineage analyses here, we noticed that the hindgut becomes distended or swollen before the anus opens (Fig. 13j). At that point, the intestine collapses down to a narrow tube and cylindrical waist products begin to be expelled. In some of the injected cases, there was no lineage tracer observed, which resulted from the accidental death of those injected 2d cells (data not shown). In those same cases, the anus does not appear to open and the hindgut remains swollen, even though ectoderm covers the terminal end of the intestine. These findings suggest that only the progeny of 2d may form the anus, and that these may be the only cells competent to respond to inductive interactions from the terminal hindgut endoderm. It is possible that differences in the spatial relationships and inductive interactions between the hindgut (endodermal) terminus and the overlying ectoderm could account for species-specific variations in those cells that have been reported to give rise to the anus.

### Differences between *Crepidula* blastopore morphogenesis and amphistomy

The relationship between the blastopore and the mouth and anus is under debate [2, 5, 7, 8, 10, 22, 23]. The fact that there is variation in the site and behavior of the blastopore takes on particular evolutionary significance because the blastopore is developmentally related both to the blind gut of bilaterian out groups, like cnidarians, and to the through-gut of bilaterians. Thus, it is reasoned that diverse bilaterian body-plans are related to, or even dependent on, changes to the site or behavior of the blastopore. How did these changes come about? According to one set of hypotheses, the bilaterian ancestor exhibited amphistomy, where a slit-like blastopore gave rise to both mouth and anus [3, 5]. According to this theory, protostomy and deuterostomy evolved from amphistomy as openings were retained at only the anterior or posterior end of the blastopore. An alternative scenario argues that deuterostomy is ancestral among bilaterians, including ecdysozoans and spiralians/lophotrochozoans, and thus protostomy arose independently on multiple occasions as a modification of deuterostomy [6]. Thinking even more broadly, it has even been called into question if there are technically any prostostomes among the bilaterians, because the blastopore forms at the posterior pole in the Spiralia, but the mouth forms in the anterior ventral ectoderm from cells that were born near the animal pole [2, 13]. The detailed cell lineage studies that are necessary to distinguish between these hypotheses are often lacking within the spiralian/lophotrochozoan branch.

Very few examples of amphistomy, or deuterostomy, are reported among species with spiral cleavage, and no lineage tracing study has definitively documented either phenomenon [7, 10]. Despite the lack of strong evidence, the gastrulation behaviors of several species have been called upon to support the existence of amphistomy, or the remnants of an amphistomous ancestor. For example, some point to Woltereck’s description of a slit-like blastopore in the annelid *Polygordius* as an extant example of amphistomy [5, 8, 54]. Yet, others disagree with this interpretation, pointing out that Woltereck himself stated, and illustrated, that the posterior portion of the blastopore closes, while the anus arises from 2d-derived cells in a more posterior location [7, 12], which is similar to what we observed in *Crepidula*. The behavior of extant species has been taken as evidence of a transition from amphistomy to protostomy. The development of *Patella* [43], *Hydroides* [17], and *Platynereis* [23] has been interpreted in this way because the blastopore is said to seal up along the posterior ventral midline. The zippering phenomenon that we observed in *Crepidula* might be homologous to the gastrulation processes in these other spiralian species. However, no lineage tracing has correlated the position of clones around the blastopore with the formation of the mouth or anus in these species. By emphasizing the behavior of the blastopore lip cells, and defining them as those cells that surround the endoderm/mesoderm in *Crepidula* (Fig. 16), we hope to establish a basis of comparison with other species.

Central to the definition of amphistomy are the notions that in the amphistomous ancestor: 1) the lateral edges of the blastopore fused, which 2) left persistent anterior and posterior holes, that 3) resulted in the simultaneous formation of the openings that became those of the mouth and anus, respectively. However, the behavior of the *Crepidula* blastopore deviates from these definitions in several respects. First, zippering of the posterior blastopore lip occurs in a posterior to anterior direction, rather than initiating at the lateral edges of the lip. Second, only one hole persists after gastrulation and this becomes the mouth. Third, it is not just the anterior portion of the blastopore lip that gives rise to the mouth, but clones from anterior, lateral, and posterior positions (Fig. 16). Fourth, the mouth also forms from anterior, lateral, and posterior cells that were never part of the blastopore lip (Figs. 14, 15, and 16). Fifth, the anus arises as a separate hole from the blastopore, days after gastrulation has finished.

Some might view the behavior of cells at the posterior blastopore lip in *Crepidula* as an example of the proposed transition from amphistomy to protostomy (see [3, 5]), At the beginning of gastrulation (~90 hpf, Fig. 1d; Fig. 14, second row; Fig. 16a), each of second and third quartet micromere lineages contribute progeny to the lip, including cells that give rise to the mouth (2a–2c, 3a–3d) and much later, to the anus (2d). In this context, one could claim that cells of the early blastopore lip include progenitors that form both the mouth and the anus. We note, however, that the progenitor cells that give rise to the anus are part of the blastopore lip for only a short duration, before being quickly excluded by the zippering of the 3c^2^ and 3d^2^ clones. Furthermore, during the time the 2d clone is part of the blastopore lip, the anus has not yet formed. Finally, we note that we have chosen to define the blastopore lip at a very early time in development (Fig. 16a), by virtue of the fact that the ectodermal micromere cap internalizes the macromeres from the very beginning of epiboly. If however, we took a more conservative approach and said that the blastopore lip does not form until later, when an obvious depression/archenteron forms (Fig. 16b, c), then the 2d clone would not be present at the blastopore lip. The definition of when the blastopore/blastopore lip forms is somewhat subjective and might vary species to species. These semantic debates reflect how difficult it can be to make direct comparisons when talking about distantly related animals whose gastrulation processes are diverse; by making an explicit definition of the blastopore lip, we hope to make future comparisons with other animals more straightforward.

While we cannot exclude the possibility that *Crepidula* blastopore morphogenesis has been modified from an ancestral amphistomy condition, we note that no extant species has been shown to exhibit amphistomy, and classic examples of amphistomy have been called into question by many authorities [7, 11, 12]. Therefore, we argue that it is unlikely that protostomy is a modification of amphistomy. If deuterostomy is ancestral to the bilaterians, as has been recently proposed (based on its widespread existence in extant deuterostomes and ecdysozoans [6, 11]), it will be of interest to understand the morphogenetic changes to cells around the blastopore that permitted such a transition. We propose that it will be more useful to focus on homologizing *cells* at the blastopore lip (which can be lineage-traced, or assayed for expression of regulatory genes), than to focus on the blastopore *hole* itself.

## Conclusions

### A morphogenetic perspective on the evolution of spiralian gastrulation

Very little is understood about the underlying morphogenetic processes that occur during spiralian development. Focusing on behaviors of specific lineages will be very informative for comparative studies. Examining the cells that make the blastopore in different species is just one example. We mention a few additional examples below.

We found that the 3q^2^ cells exhibit highly dynamic behaviors in *Crepidula*. Cells derived from 3a^2^ and 3b^2^ undergo EMT during formation of the ectomesoderm, and the 3c^2^ and 3d^2^ cells undergo convergence and extension to close the posterior blastopore lip. These cells display other interesting differences: the 3a^2^/3b^2^ cells divide multiple times (giving rise to dozens of cells each), never become ciliated, and only form filopodia after they have become mesenchymal. Conversely, the 3c^2^/3d^2^ cells divide only twice (giving rise to just four cells each), become multi-ciliated, and form filopodia while still remaining in the ectoderm. How these third quartet cells acquire their unique identities will be an interesting area of future research. In *Ilyanassa*, inheritance of mRNA can distinguish different tiers of micromeres along the animal vegetal axis [74] while differential stability of mRNAs along the anterior-posterior/dorsal ventral axis can then distinguish different quadrants [50]. For example, the *Tis11* transcript is initially inherited by all 3q micromeres, but is retained in only the 3a and 3b cells, which form ectomesoderm [18, 50]. In other species, like *Platynereis* and *Capitella*, the 3c and 3d cells contribute to ectomesoderm, and it would be interesting to compare cellular and molecular events within these different tiers and quartets of micromeres to understand the basis of this inter-species variation.

Another fruitful area of research would be comparing species that gastrulate by epiboly, emboly, and invagination [10]. For example, in *Crepidula*, the blastopore lip has to narrow considerably to cover the large endodermal macromeres; the zippering behavior we observed in the posterior blastopore lip might be necessary to cover those cells. During invagination, in contrast, the endodermal cells are smaller, and the ectodermal cells near them might behave differently. Furthermore, it would be interesting to study how similar the movement of cells towards the ventral midline in *Patella*, *Hydroides*, and *Platynereis* are to that in *Crepidula*. In *Capitella*, the blastopore has been described to close completely [19], which might be accomplished by a more extensive zippering behavior, or a completely distinct mechanism. Studying the details of cell lineages and their behaviors will address how such variation arose.

Finally, spiralians are one of the only groups of metazoans where homology of body-plans can be compared at the levels of gene expression and cell lineage. Comparison of gene expression patterns in the posterior and anterior blastopore lip has been used in arguments about amphistomy [23, 43, 75], but in the absence of detailed lineage tracing during gastrulation stages. It has been proposed that the differences in gene expression for endomesodermal markers might be a reflection of different life history strategies [17], which also correlate with different modes of gastrulation [10]. To test this interesting hypothesis, it will be necessary to have both gene expression and fate map data for multiple species [76]. Our goal here is to provide a framework for comparative studies of morphogenesis, in the hope that *Crepidula* will provide a useful point of reference for similar studies in other species.

## Additional files

Additional file 1:
**Movie 1.mov (Figure S1).** Time lapse light sheet confocal images of an embryo expressing the microtubule-cytoskeleton bio-sensor EMTB-GFP (*green*) and an RFP-membrane biosensor (*red*). Corresponds to Figure 1s. Additional file 2:
**Movie 2.mov (Figure 2f).** Time-lapse movie of embryos expressing UTPH-GFP during early epiboly. Corresponds to Figure 2f. Additional file 3
**Movie 3.mov (Figure 2g).** Time-lapse movie of embryos expressing UTPH-GFP during later epiboly. Corresponds to Figure 2g. Additional file 4:
**Movie 4.mov (Figure 2h).** Time-lapse movie of embryos expressing UTPH-GFP during elongation. Corresponds to Figure 2h. Additional file 5:
**Movie 5.mov (Figure 2i).** Time-lapse movie of embryos expressing UTPH-GFP during mouth formation. Corresponds to Figure 2i. Additional file 6:
**Movie 6.mov (Figure 9a).** Time-lapse movie of an embryo injected with utrophin-GFP (UTPH) to mark cell outlines (*green*), and histone H2B-RFP (H2B) to mark nuclei (*yellow*). Ventral view. Corresponds to Figure 9a–i. Additional file 7:
**Movie 7.mov (Figure 9j).** Time-lapse movie of an embryo injected with ensconsin-GFP (EMTB) to mark microtubules (*green*), and histone-RFP (H2B) to mark nuclei (*yellow*). Ventral view. Corresponds to Figure 9j–u. Additional file 8:
**Movie 8.mov (Figure 9v).** Spinning disk confocal time-lapse of an embryo expressing utrophin-GFP (UTPH) globally (*green*), and in which the 3b micromere was labeled with diI (*red*). Ventral view. Corresponds to Figure 9v–z. Additional file 9:
**Movie 9.mov (Figure 12b).** Time-lapse movie of an embryo undergoing convergence and extension to zipper the posterior blastopore closed. The 3c cell was injected with dextran (*green*) and the 3d cell was injected with diI (*red*). Corresponds to Figure 12b–l. Additional file 10:
**Movie 10.mov (Figure 12w).** Confocal time-lapse movie of 3c^2^ cells injected with diI, showing the beating of cilia. Corresponds to Figure 12w. Additional file 11:
**Figure S1.** Early epiboly and position of clones at the blastopore lip. **a–h** Cartoons of early embryo with second and third quartet micromeres colored, as indicated in key to the right. **a–c** Animal pole views; **d** is a ventral/vegetal view; **e–h** are lateral views. Black and white cartoons are modified from Conklin’s drawings [42]. **i–r** Images of embryos during late cleavage and early epiboly stages labeled with the actin cytoskeleton marker UTPH-GFP (*white*) and histone H2B-RFP (red); in some panels, the 4d clone is labeled with diI (red). *AV* animal pole view, *VV* ventral/vegetal pole view, *LV* lateral view. The 4d clone can often be identified without direct labeling because the UTPH-GFP is preferentially expressed or stabilized in this clone (e.g. as in **l, m, q, r**). Scale bar equals 50 μm. **s–w** Time lapse light sheet confocal images of an embryo expressing the microtubule-cytoskeleton bio-sensor EMTB-GFP and an RFP-membrane biosensor (MEM-RFP). Lateral view. Many cells are seen to divide over the course of the time-lapse (*arrow* mitotic spindle), but the micromere cap does not make a significant advance towards the vegetal pole during this period. See also Additional file 1, which corresponds to panels **s–w**. Additional file 12:
**Figure S2.** Narrowing of the blastopore during later epiboly, and formation of the mouth/stomodeum. **a–e** Confocal images of living embryos expressing UTPH-GFP to mark the actin cytoskeleton, and histone H2B-RFP to mark the nuclei. Ventral views are shown during mid epiboly (**a**), late epiboly (**b**), elongation (**c**), and mouth formation (**d–e**). **f–i** Time-lapse movies of embryos expressing UTPH-GFP during epiboly and mouth formation. *VV* ventral view. *Scale bar* equals 50 μm. See also Additional files 2, 3, 4, and 5, which correspond to panels **f, g, h,** and **i**. Additional file 13:
**Figure S3.** Fates of micromere 2a, and its subclones, during gastrulation and organogenesis. Images of live embryos with dextran- and diI-labeled 2a, or 2a subclones, as indicated. In some cases, the zygote was previously injected with mRNAs coding for fluorescent fusion proteins for histone H2B-RFP (H2B) and/or the actin-binding domain of utrophin-GFP (UTPH) to visualize nuclei or cell outlines, respectively, where indicated. Anterior is up in all cases. **a** Ventral view during early epiboly. Corresponding ventral-view images are shown in **b-c**, **d-e**, **f-g** during late epiboly with different combinations of fluorescence and/or DIC layers shown. **h** Shows a dorsal view of the same embryo shown in **f-g. i, j** Ventral views of older elongating embryos. **k, l** Corresponding right lateral views of an older embryo undergoing organogenesis. **m** Left lateral view of an older embryo undergoing organogenesis. **n, o** Corresponding left lateral view of an older embryo undergoing organogenesis. *bp* blastopore, *ft* foot, *hg* hindgut rudiment, *mt* metatroch, *pgc* primordial germ cell, *rtc* right terminal cell, *sg* shell gland, *st* stomodeum/mouth. *Scale bar* equals 50 μm. Additional file 14:
**Figure S4.** Fates of micromere 2b, and its subclones, during gastrulation and organogenesis. Images of live embryos, with dextran and diI-labeled 2b, or 2b subclones, as indicated. In some cases, the zygote was previously injected with mRNAs coding for fluorescent fusion proteins for histone H2B-RFP (H2B) and/or the actin-binding domain of utrophin-GFP (UTPH) to visualize nuclei or cell outlines, respectively, where indicated. Anterior is up in all cases. **a** Ventral view during an early stage of epiboly. **b** Ventral view during a later stage of epiboly. Corresponding ventral views are shown in **c-d**, **e-f**, **g-h**, **i-j** during epiboly with different combinations of fluorescence and/or DIC layers shown. Corresponding dorsal images are shown in **k-l**, **m-n** during epiboly. **o–q** Ventral views of older, elongated embryos. **r** Oblique left-lateral view of the ventral surface of an older embryo. Corresponding left-lateral images are depicted in **s-t** and **x-y** of older embryos undergoing organogenesis. **u** Right lateral view of an older embryo undergoing organogenesis. **v, w** Corresponding oblique left-lateral views of the ventral surface of an older embryo undergoing organogenesis. *fg* food groove, *nc* neural cells. Other labels are the same as those used in Fig. 3. *Scale bar* equals 50 μm Additional file 15:
**Figure S5.** Fates of micromere 2c, and its subclones, during gastrulation and organogenesis. Images of live embryos with dextran and diI labeled 2c, or 2c subclones, as indicated. In some cases, the zygote was previously injected with mRNAs coding for fluorescent fusion proteins for histone H2B-RFP (H2B) and/or the actin-binding domain of utrophin-GFP (UTPH) to visualize nuclei or cell outlines, respectively, where indicated. Anterior is up in all cases. **a, b** Corresponding ventral and dorsal views of an embryo near the end of gastrulation with different combinations of fluorescence and/or DIC layers shown. **c, d** Corresponding ventral views of an embryo near the end of gastrulation. **e, f** Corresponding ventral views of a slightly older embryo at the end of gastrulation. **g, h** Corresponding ventral views of an older elongating embryo. **i** Dorsal view of an elongating embryo. **j** Ventral surface view of an embryo at the onset of organogenesis. **k, l** Corresponding right lateral views of an embryo at the onset of organogenesis. **m, n** Corresponding right lateral views of an older embryo during organogenesis. **o, p** Corresponding oblique dorsal view of an embryo during organogenesis. Corresponding right-lateral views of embryos undergoing organogenesis are shown in **q-r**, **s-t**. *tc* terminal cells. Other labels are the same as those used in Fig. 3. *Scale bar* equals 50 μm. Additional file 16:
**Figure S6.** Fates of micromere 2d, and its subclones, during gastrulation and organogenesis. Images of live embryos, with dextran and diI-labeled 4d, 2d, or 2d subclones, as indicated. In some cases, the zygote was previously injected with mRNAs coding for fluorescent fusion proteins for histone H2B-RFP (H2B) and/or the actin-binding domain of utrophin-GFP (UTPH) to visualize nuclei or cell outlines, respectively, where indicated. Animal pole is up in **a**, anterior is up in **b–dd. a** Dorso-lateral view of an early epiboly-stage embryo. Corresponding ventral views of embryos during epiboly are shown in **b-c**, **d-e**, **f-g** with different combinations of fluorescence and/or DIC layers shown. **h, i** Corresponding dorsal views of same embryo shown in **f, g**. Corresponding ventral view images of successively older embryos undergoing epiboly are shown in **j-k**, **l-m. n, o** Shows corresponding dorsal views of an embryo at the same stage as that shown in **l, m. p** Ventral surface view of an elongated embryo. **q** Ventral view of embryo undergoing elongation. Note unlabeled voids where the two terminal cells (tc) from 3c^221^ and 3d^221^ reside. **r, s** Corresponding ventral views of embryo undergoing elongation. **t, u** Right-lateral views of older embryo at the onset of organogenesis. **v, w** Corresponding left-lateral views of embryo at the onset of organogenesis. **x, y** Ventral views of embryo undergoing elongation. Unlabeled voids occupied by the two terminal cells (*tc*) are also indicated in **x**. Corresponding left-lateral (**z, aa**) and right-lateral/oblique (**bb, cc**) views of older embryos during organogenesis. **dd** Shows a ventral view of an embryo during organogenesis. *pb* polar body, *ns* neurosensory cell. Other labels are the same as those used in Figs. 3 and 4. *Scale bar* equals 50 μm. Additional file 17:
**Figure S7.** Fates of micromere 3a, and its subclones, during gastrulation and organogenesis. Images of live embryos, with dextran and diI-labeled 4d, 3a, or 3a subclones, as indicated. In some cases, the zygote was previously injected with mRNAs coding for fluorescent fusion proteins for histone H2B-RFP (H2B) and/or the actin-binding domain of utrophin-GFP (UTPH) to visualize nuclei or cell outlines, respectively, where indicated. Animal pole is up in **a**. Anterior is up in **b–o. a** Dorso-lateral view of an early epiboly-stage embryo. **b** Ventral (vegetal) view of early epiboly-stage embryo. **c-d**, **e-f** Show corresponding ventral views of early and mid epiboly-staged embryos, respectively, with different combinations of fluorescence and/or DIC layers shown. **g, h** Corresponding ventral views of later stage embryos undergoing epiboly. **i** Ventral view of an older, elongating embryo. **j, k** Ventral views of two embryos just prior to the onset of organogenesis. Note that for the original stack of confocal images shown as a projection in **j**, the 3a^1^ and 3a^2^ clones are spatially separated in the *Z* axis, making it possible to pseudocolor them separately, as labeled. Corresponding ventral views of embryo just prior to the onset of organogenesis are shown in **l, m, n, o** Left-lateral view of an embryo during organogenesis. *cb* ciliary band, *ms* mesenchyme. Other labels are the same as those used in Figs. 3 and 6. *Scale bar*equals 50μm. Additional file 18:
**Figure S8.** Fates of micromere 3b, and its subclones, during gastrulation and organogenesis. Images of live embryos, with dextran and diI-labeled 4d, 3b, or 3b subclones, as indicated. In some cases, the zygote was previously injected with mRNAs coding for fluorescent fusion proteins for histone H2B-RFP (H2B) and/or the actin-binding domain of utrophin-GFP (UTPH) to visualize nuclei or cell outlines, respectively, where indicated. Animal pole is up in **a**. Anterior is up in **b–o. a** Lateral-dorsal view of an early epiboly-stage embryo. **b-c**, **d-e** show corresponding ventral (vegetal) views of early epiboly-staged embryos with different combinations of fluorescence and/or DIC layers shown. **f** Ventral view of embryo during later epiboly. **g, h** Show ventral views of two stages of mesenchyme migration. **i** Ventral view showing numerous mesenchyme cells. **j, k** Corresponding right-lateral views of embryos during organogenesis. **l, m** Ventral surface views. **n, o** Corresponding right-lateral views of older embryos during organogenesis. Labels are the same as those used in Figs. 3, 6, and 7. *Scale bar* equals 50 μm. Additional file 19:
**Figure S9.** Behavior of ectomesoderm (3a^2^, 3b^2^). **a–i** Time-lapse movie of an embryo injected with utrophin-GFP (UTPH) to mark cell outlines, and histone H2B-RFP (H2B) to mark nuclei, where indicated. Ventral view. *bp* blastopore. Several unidentified (x) daughter cells of 3a^2^, 3b^2^ are marked, *in colors*, to show the orientation of cell division and position of ectomesodermal precursor cells during the narrowing of the anterior blastopore lip. *Scale bar* in **f** equals 50 μm; *scale bar* in **i** equals 25 μm. **j–u** Frames from a timelapse movie of an embryo injected with ensconsin-GFP (EMTB) to mark microtubules, and histone-RFP (H2B) to mark nuclei. Ventral view. *Dashed white lines* outline the 3b^2^ clone. *Scale bar* in **u** equals 30 μm. **v–z** Spinning disk confocal frames from a time-lapse of an embryo expressing utrophin-GFP (UTPH) globally, and in which the 3b micromere was labeled with diI (red). Ventral view. *Scale bar* in **z** equals 50 μm. See also Additional files 6, 7, and 8. Additional file 20:
**Figure S10.** Fates of micromere 3c, and its subclones, during gastrulation and organogenesis. Images of live embryos, with dextran and diI-labeled 4d, 3c, or 3c subclones, as indicated. Animal pole is up in **a** and **b**. Anterior is up in **c–t. a, b** Dorso-lateral views of early epiboly-stage embryos. **c, d** Ventral views of early epiboly-stage embryos. Corresponding ventral views of late epiboly-stage embryos are shown in **e-f**, **g-h**, **i-j** at successive stages of development with different combinations of fluorescence and/or DIC layers shown. **k, l** Corresponding ventral and posterior views of an elongating embryo. **m, n** Ventral views of embryos during elongation. Corresponding right-lateral views of embryos during organogenesis **o-p**, **s-t**. Note *green* background fluorescence is higher in **s, t. (Q-R)** Corresponding oblique, ventro-lateral views of an embryo during organogenesis. *nt* neurotroch, *rtc* right terminal cell, *pvb* posterior velar band. All other labels are the same as those used in Figs. 3 and 6. *Scale bar* equals 50 μm. Additional file 21:
**Figure S11.** Fates of micromere 3d, and its subclones, during gastrulation and organogenesis. Images of live embryos, with dextran and diI-labeled 4d, 3d, or 3d subclones, as indicated. In some cases, the zygote was previously injected with mRNAs coding for fluorescent fusion proteins for the actin-binding domain of utrophin-GFP (UTPH) and histone H2B-RFP to visualize nuclei or cell outlines, respectively, where indicated. Animal pole is up in **a** and **b**. Anterior is up in **c–y. a, b** Dorso-lateral views of early epiboly-stage embryos. **c, d** Ventral views of early epiboly stage embryos. **e–j** Ventral views of elongating embryos later during epiboly. **k, l** Corresponding ventral views of an embryo during elongation with different combinations of fluorescence and/or DIC layers shown. **m** Right-lateral higher magnification views of an elongating embryo with inserts showing some of the ventral ciliated cells (shallow confocal stacks centered at the ventral midline). **n, o** Corresponding ventral views of embryos during organogenesis. Corresponding left lateral views of embryos during organogenesis **p-q**, **r-s**, **t-u**. Corresponding ventral views of early veliger stage embryos **v-w**, **x-y**. *ltc* left terminal cell. All other labels are the same as those used in Figs. 3, 6, and 10. *Scale bar* equals 50 μm for **a–l** and **n–y**. *Scale bar* equals 25 μm for **m** and 20 μm for its inserts. Additional file 22:
**Figure S12.** Behavior of posterior blastopore lip cells undergoing convergence and extension (3c^2^, 3d^2^). **a–aa** Projected confocal Z slices of embryos injected with dextran or diI, into 3c, 3d or their subclones, as indicated. **a** Ventral view of an embryo at epiboly stage. **b–u** Frames from a time-lapse of the same embryo as shown in **a. b–l** Frames from a time-lapse movie of the same embryo undergoing convergence and extension to zipper the posterior blastopore (*bp*) closed. **m–r** Frames from the same movie showing membrane protrusions (arrows) and cilia (arrow heads) on the cells undergoing convergent extension (zippering). **s, t** Frames from the same movie showing that the cells undergoing convergence and extension are from the 3c^2^ and 3d^2^ cells. The *asterisk* (*) marks cells from the 3c^1^ and 3d^1^ clones that migrate anteriorly towards the ventral side of the stomodeum. In **u**, the 3c^211^ and 3d^211^ cells have entered the deeper parts of the mouth an are out of view of the stack, indicated by *dashed lines*. **v–aa** Live confocal images of post-zippering-stage embryos showing the movement of the 3c^221^ and 3d^221^ cells posteriorly, between cells of the 2d clone. **bb–mm** Images of fixed embryos stained for acetylated tubulin (to mark cilia) and DAPI (to mark DNA). **bb, cc** Shows ventral views (**vv**) of embryos at the early stages of convergent extension and *arrowheads* point to cilia on cells at the posterior edge of the blastopore. **dd–ff** Ventral views of embryos during elongation. The ciliated left and right terminal cells (*ltc*, *rtc*) are labeled. **gg** Posterior view (*pv*) of the same embryo shown in **ff**. *Scale bar* in **l** and **mm** are equal to 50 μm; *scale bar* in **v** equals 20 μm. See also Additional files 9 and 10. Additional file 23:
**Figure S13.** Origin of the anus (2d^2^). Images of live embryos, with dextran and diI-labeled 4d, 3d, 3c, 2d, or 2d subclones, as indicated. In some cases, the zygote was previously injected with mRNAs coding for fluorescent fusion protein for the actin-binding domain of utrophin-GFP (UTPH) to visualize cell outlines, where indicated. Anterior is up in all cases. **a–c** Corresponding ventral views of embryos during organogenesis with different combinations of fluorescence and/or DIC layers shown. **d, e** Corresponding right-lateral (oblique-ventral) views of embryo during organogenesis. **f, g** Corresponding higher magnification right-lateral views of the posterior end of an embryo during organogenesis. **h, i** Corresponding ventral views of veliger stage embryo after the anus as opened. **j** Ventral view of a veliger stage embryo just before the anus opens. Note that the hindgut is somewhat swollen. **k, l** Corresponding ventral views of pre-veliger stage embryos. **m, n** Corresponding (oblique-ventral) left-lateral views of the pre-veliger stage embryos. **o** Ventral view of early veliger. **p, q** Corresponding (oblique-ventral) left-lateral views of an early veliger. **r, s** Corresponding ventral views of an early veliger. *an* anus, *nc* neural cell, *ns* neurosensory cell, *rm* right mantle, *tcs* terminal cells (called *tc* in Fig. 6). All other labels are the same as those used in Figs. 3, 4, 10, and 11. *Scale bar* equals 50 μm for **a–e**, **h–s**. *Scale bar* equals 25 μm in **f, g**. Additional file 24:
**Figure S14.** Summary of second and third quartet clones. *Columns* show clones, colored as labeled, and according to those shown in Figs. 1 and 15. *Rows* show time points as indicated at the far right. *Top row* shows animal views; all other rows show ventral views. The smaller, irregularly shaped, stippled cells of the 3a^2^ and 3b^2^ clones represent ecto-mesenchyme located below the ectoderm Additional file 25:
**Figure S15.** Lineage diagram of second and third quartet micromeres and third quartet macromeres. *Colors* correspond to those given in Figs. 1 and 14. Additional file 26:
**Figure S16.** Morphogenesis of the blastopore lip. **a–e** Vegetal/ventral views during gastrulation with the future anterior at the top of the figure. Time points are the same as those shown in rows 2–6 in Figs. 1 and 14: **a** ~94 hpf, **b** ~99 hpf, **c** ~120–130 hpf, **d** ~130–137 hpf, **e** ~140–145hpf. The *coloring* shows the relative clonal contributions of cells within the embryo and to the blastopore lip. The blastopore lip is marked in each panel by a *solid white line*. The blastopore lip marks the boundary between the ectodermal micromere cap and the endoderm/endomesoderm/ectomesoderm. As gastrulation proceeds, some cells leave the blastopore lip, but remain on the surface, and these are marked by a *dashed white line*. The 2d cells are marked by a *dashed yellow line* in **a, b**, but are more difficult to follow in time points **c–e** and thus are not shown. The 3c^2^- and 3d^2^-derived terminal cells (TC) are marked with an *asterisk* to follow their migration after they leave the blastopore lip. Some cells of the blastopore lip move internally into the blastocoel/archenteron, and the *solid white line* can no longer be seen in some areas **d, e**. *bp* blastopore, *em* ectomesoderm (derived from 3a^2^ and 3b^2^).

## Abbreviations

Hpf: hours post-fertilization
Q: macromere quartet
q: micromere quartet
EMT: epithelial-to-mesenchymal transition
