# Supplementary figures and images for: Spiralian gastrulation: germ layer formation, morphogenesis, and fate of the blastopore in the slipper snail Crepidula fornicata
Source: EvoDevo. 2015 Jun 24;6:24. doi: 10.1186/s13227-015-0019-1 (PMC4673862; doi:10.1186/s13227-015-0019-1)

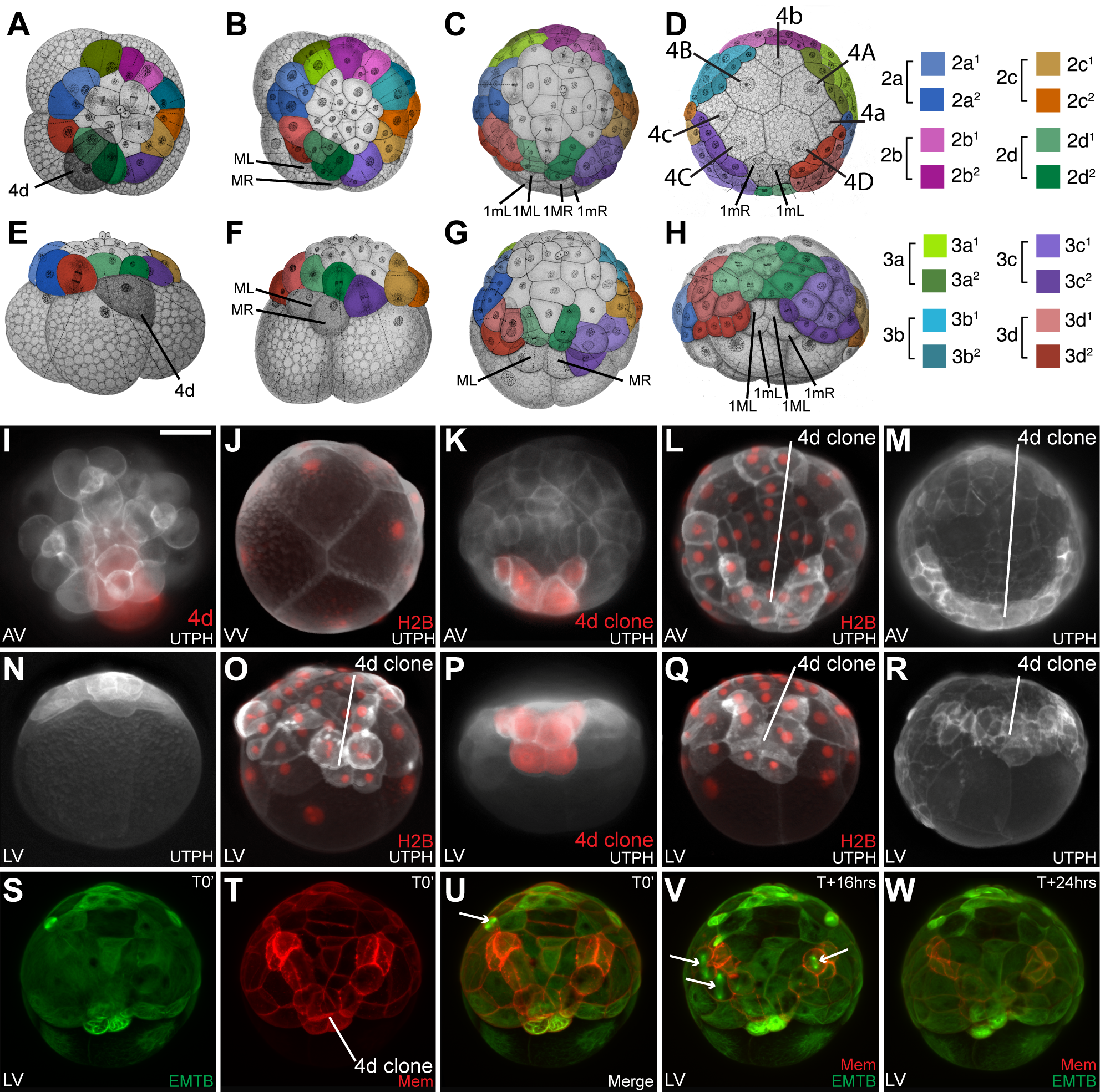

Supplement: Additional file 11: — Figure S1. Early epiboly and position of clones at the blastopore lip. a–h Cartoons of early embryo with second and third quartet micromeres colored, as indicated in key to the right. a–c Animal pole views; d is a ventral/vegetal view; e–h are lateral views. Black and white cartoons are modified from Conklin’s drawings [42]. i–r Images of embryos during late cleavage and early epiboly stages labeled with the actin cytoskeleton marker UTPH-GFP (white) and histone H2B-RFP (red); in some panels, the 4d clone is labeled with diI (red). AV animal pole view, VV ventral/vegetal pole view, LV lateral view. The 4d clone can often be identified without direct labeling because the UTPH-GFP is preferentially expressed or stabilized in this clone (e.g. as in l, m, q, r). Scale bar equals 50 μm. s–w Time lapse light sheet confocal images of an embryo expressing the microtubule-cytoskeleton bio-sensor EMTB-GFP and an RFP-membrane biosensor (MEM-RFP). Lateral view. Many cells are seen to divide over the course of the time-lapse (arrow mitotic spindle), but the micromere cap does not make a significant advance towards the vegetal pole during this period. See also Additional file 1, which corresponds to panels s–w. [file 13227_2015_19_MOESM11_ESM.tiff]

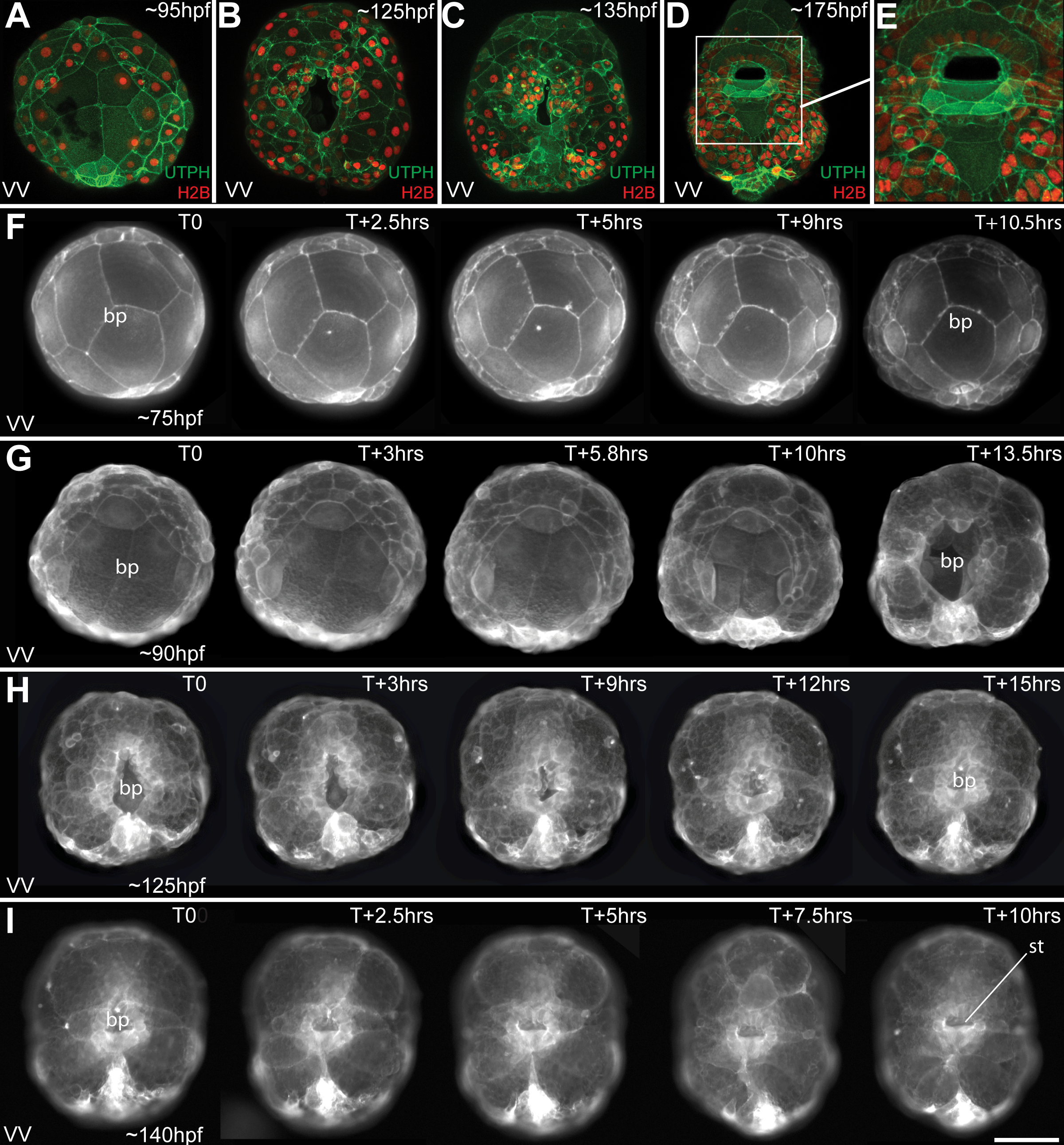

Supplement: Additional file 12: — Figure S2. Narrowing of the blastopore during later epiboly, and formation of the mouth/stomodeum. a–e Confocal images of living embryos expressing UTPH-GFP to mark the actin cytoskeleton, and histone H2B-RFP to mark the nuclei. Ventral views are shown during mid epiboly (a), late epiboly (b), elongation (c), and mouth formation (d–e). f–i Time-lapse movies of embryos expressing UTPH-GFP during epiboly and mouth formation. VV ventral view. Scale bar equals 50 μm. See also Additional files 2, 3, 4, and 5, which correspond to panels f, g, h, and i. [file 13227_2015_19_MOESM12_ESM.tiff]

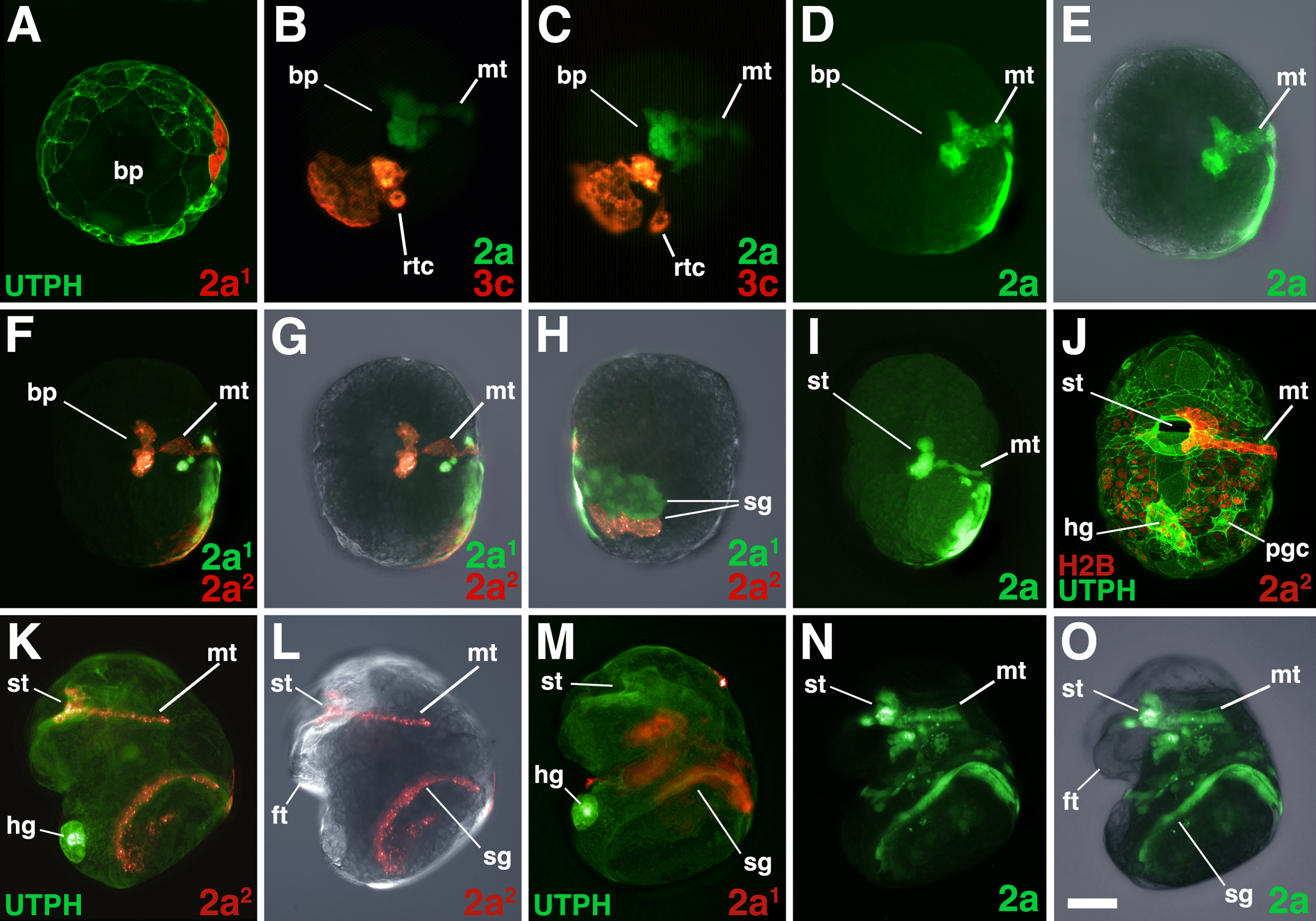

Supplement: Additional file 13: — Figure S3. Fates of micromere 2a, and its subclones, during gastrulation and organogenesis. Images of live embryos with dextran- and diI-labeled 2a, or 2a subclones, as indicated. In some cases, the zygote was previously injected with mRNAs coding for fluorescent fusion proteins for histone H2B-RFP (H2B) and/or the actin-binding domain of utrophin-GFP (UTPH) to visualize nuclei or cell outlines, respectively, where indicated. Anterior is up in all cases. a Ventral view during early epiboly. Corresponding ventral-view images are shown in b-c, d-e, f-g during late epiboly with different combinations of fluorescence and/or DIC layers shown. h Shows a dorsal view of the same embryo shown in f-g. i, j Ventral views of older elongating embryos. k, l Corresponding right lateral views of an older embryo undergoing organogenesis. m Left lateral view of an older embryo undergoing organogenesis. n, o Corresponding left lateral view of an older embryo undergoing organogenesis. bp blastopore, ft foot, hg hindgut rudiment, mt metatroch, pgc primordial germ cell, rtc right terminal cell, sg shell gland, st stomodeum/mouth. Scale bar equals 50 μm. [file 13227_2015_19_MOESM13_ESM.tiff]

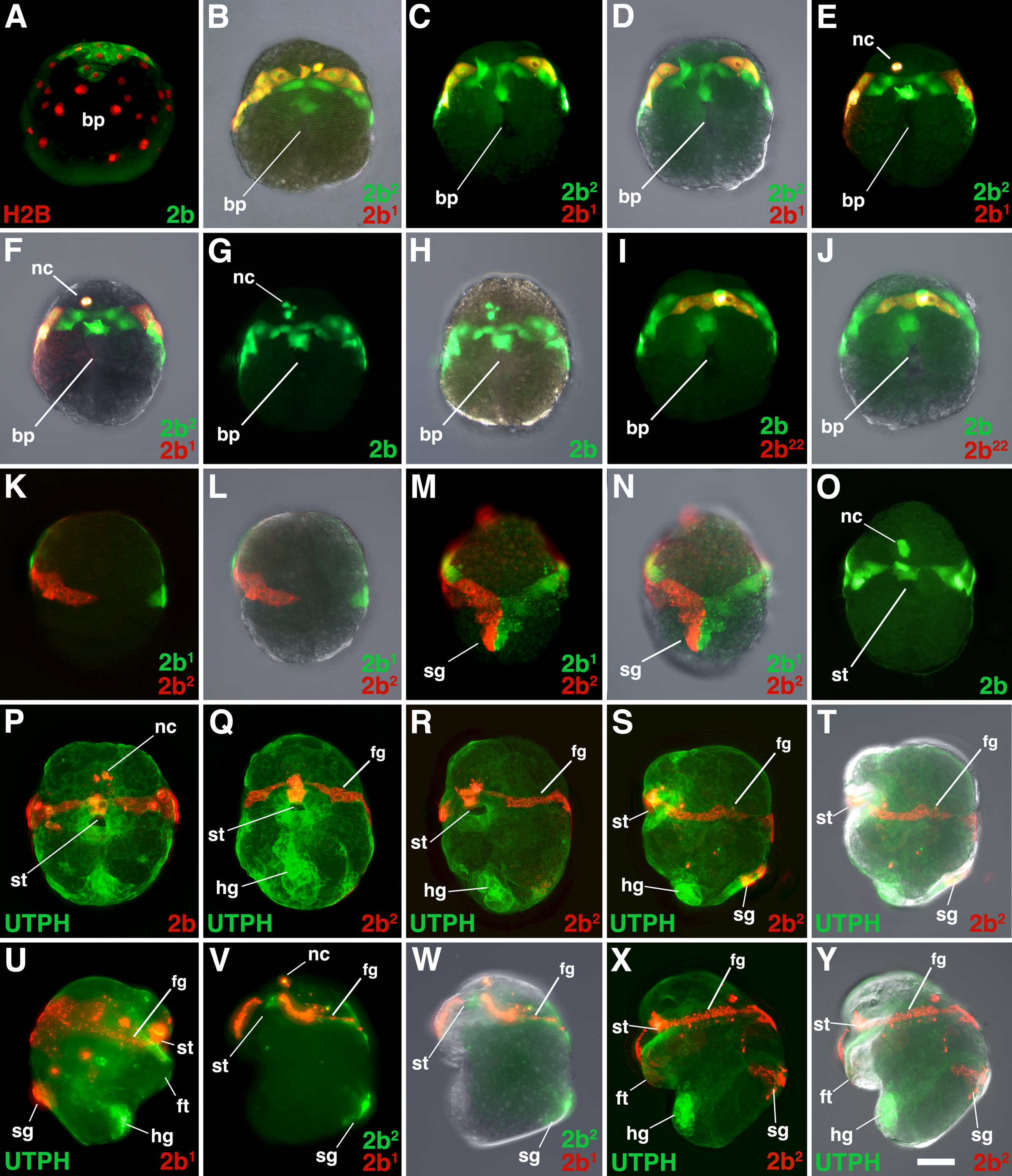

Supplement: Additional file 14: — Figure S4. Fates of micromere 2b, and its subclones, during gastrulation and organogenesis. Images of live embryos, with dextran and diI-labeled 2b, or 2b subclones, as indicated. In some cases, the zygote was previously injected with mRNAs coding for fluorescent fusion proteins for histone H2B-RFP (H2B) and/or the actin-binding domain of utrophin-GFP (UTPH) to visualize nuclei or cell outlines, respectively, where indicated. Anterior is up in all cases. a Ventral view during an early stage of epiboly. b Ventral view during a later stage of epiboly. Corresponding ventral views are shown in c-d, e-f, g-h, i-j during epiboly with different combinations of fluorescence and/or DIC layers shown. Corresponding dorsal images are shown in k-l, m-n during epiboly. o–q Ventral views of older, elongated embryos. r Oblique left-lateral view of the ventral surface of an older embryo. Corresponding left-lateral images are depicted in s-t and x-y of older embryos undergoing organogenesis. u Right lateral view of an older embryo undergoing organogenesis. v, w Corresponding oblique left-lateral views of the ventral surface of an older embryo undergoing organogenesis. fg food groove, nc neural cells. Other labels are the same as those used in Fig. 3. Scale bar equals 50 μm [file 13227_2015_19_MOESM14_ESM.tiff]

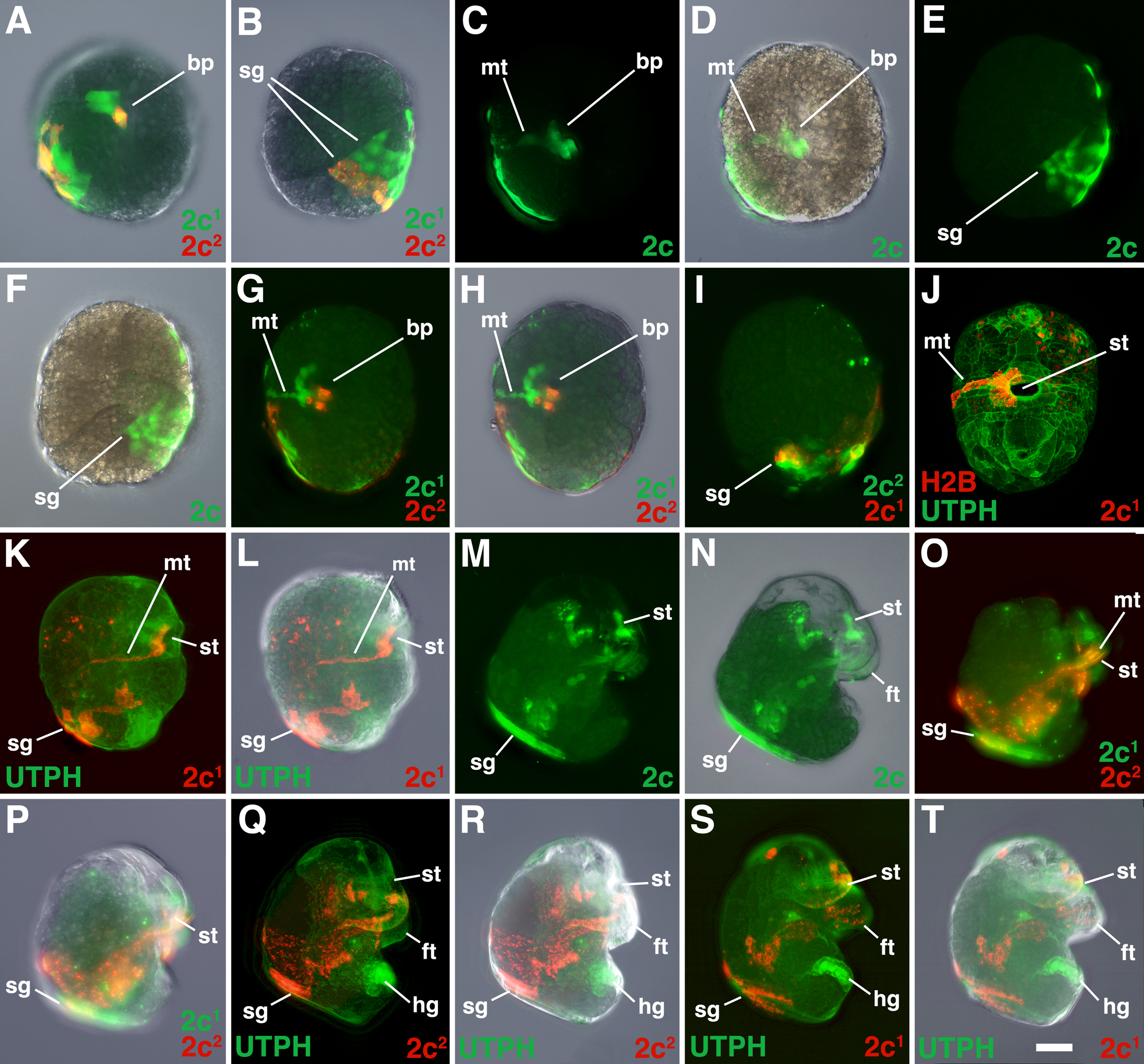

Supplement: Additional file 15: — Figure S5. Fates of micromere 2c, and its subclones, during gastrulation and organogenesis. Images of live embryos with dextran and diI labeled 2c, or 2c subclones, as indicated. In some cases, the zygote was previously injected with mRNAs coding for fluorescent fusion proteins for histone H2B-RFP (H2B) and/or the actin-binding domain of utrophin-GFP (UTPH) to visualize nuclei or cell outlines, respectively, where indicated. Anterior is up in all cases. a, b Corresponding ventral and dorsal views of an embryo near the end of gastrulation with different combinations of fluorescence and/or DIC layers shown. c, d Corresponding ventral views of an embryo near the end of gastrulation. e, f Corresponding ventral views of a slightly older embryo at the end of gastrulation. g, h Corresponding ventral views of an older elongating embryo. i Dorsal view of an elongating embryo. j Ventral surface view of an embryo at the onset of organogenesis. k, l Corresponding right lateral views of an embryo at the onset of organogenesis. m, n Corresponding right lateral views of an older embryo during organogenesis. o, p Corresponding oblique dorsal view of an embryo during organogenesis. Corresponding right-lateral views of embryos undergoing organogenesis are shown in q-r, s-t. tc terminal cells. Other labels are the same as those used in Fig. 3. Scale bar equals 50 μm. [file 13227_2015_19_MOESM15_ESM.tiff]

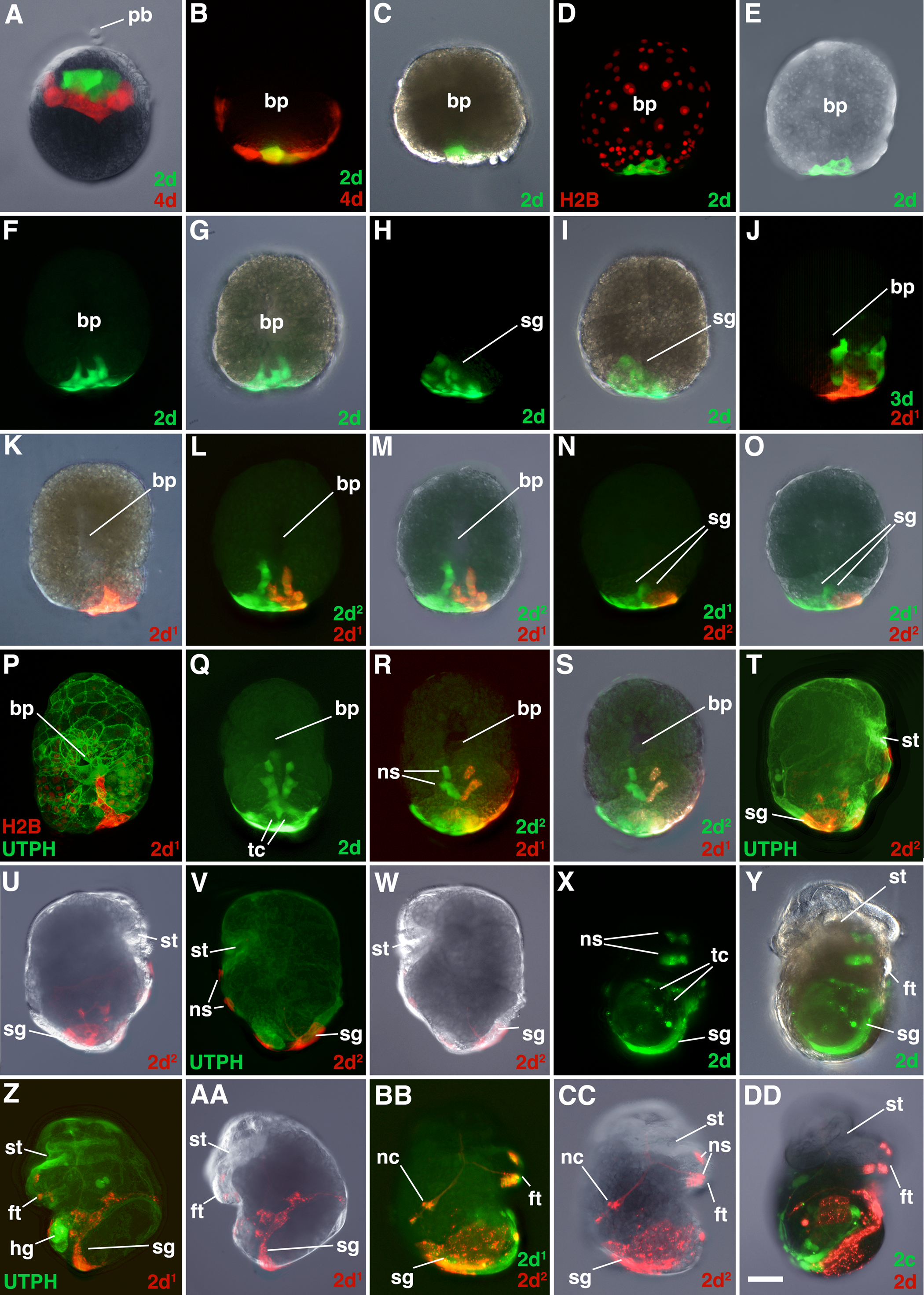

Supplement: Additional file 16: — Figure S6. Fates of micromere 2d, and its subclones, during gastrulation and organogenesis. Images of live embryos, with dextran and diI-labeled 4d, 2d, or 2d subclones, as indicated. In some cases, the zygote was previously injected with mRNAs coding for fluorescent fusion proteins for histone H2B-RFP (H2B) and/or the actin-binding domain of utrophin-GFP (UTPH) to visualize nuclei or cell outlines, respectively, where indicated. Animal pole is up in a, anterior is up in b–dd. a Dorso-lateral view of an early epiboly-stage embryo. Corresponding ventral views of embryos during epiboly are shown in b-c, d-e, f-g with different combinations of fluorescence and/or DIC layers shown. h, i Corresponding dorsal views of same embryo shown in f, g. Corresponding ventral view images of successively older embryos undergoing epiboly are shown in j-k, l-m. n, o Shows corresponding dorsal views of an embryo at the same stage as that shown in l, m. p Ventral surface view of an elongated embryo. q Ventral view of embryo undergoing elongation. Note unlabeled voids where the two terminal cells (tc) from 3c221 and 3d221 reside. r, s Corresponding ventral views of embryo undergoing elongation. t, u Right-lateral views of older embryo at the onset of organogenesis. v, w Corresponding left-lateral views of embryo at the onset of organogenesis. x, y Ventral views of embryo undergoing elongation. Unlabeled voids occupied by the two terminal cells (tc) are also indicated in x. Corresponding left-lateral (z, aa) and right-lateral/oblique (bb, cc) views of older embryos during organogenesis. dd Shows a ventral view of an embryo during organogenesis. pb polar body, ns neurosensory cell. Other labels are the same as those used in Figs. 3 and 4. Scale bar equals 50 μm. [file 13227_2015_19_MOESM16_ESM.tiff]

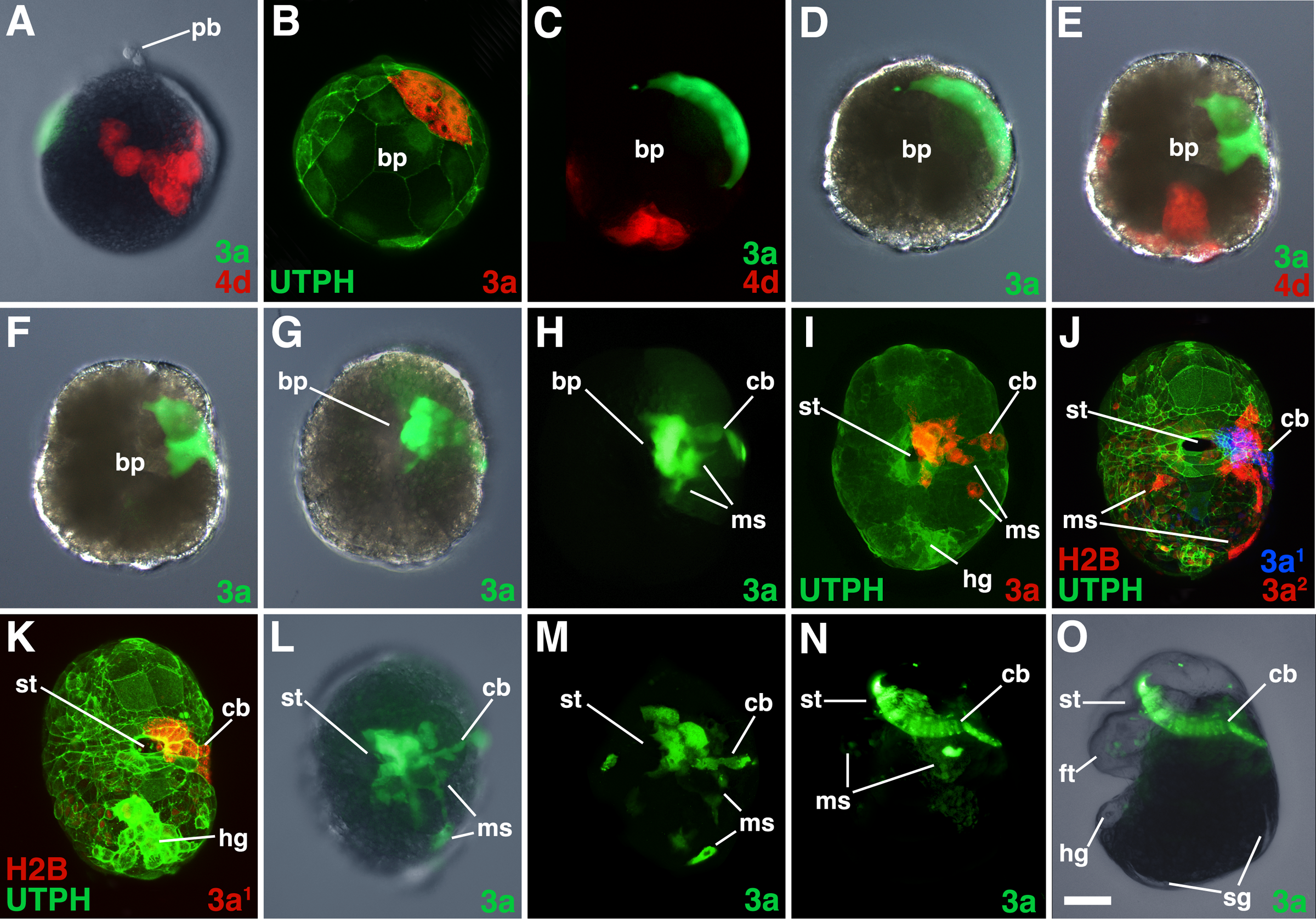

Supplement: Additional file 17: — Figure S7. Fates of micromere 3a, and its subclones, during gastrulation and organogenesis. Images of live embryos, with dextran and diI-labeled 4d, 3a, or 3a subclones, as indicated. In some cases, the zygote was previously injected with mRNAs coding for fluorescent fusion proteins for histone H2B-RFP (H2B) and/or the actin-binding domain of utrophin-GFP (UTPH) to visualize nuclei or cell outlines, respectively, where indicated. Animal pole is up in a. Anterior is up in b–o. a Dorso-lateral view of an early epiboly-stage embryo. b Ventral (vegetal) view of early epiboly-stage embryo. c-d, e-f Show corresponding ventral views of early and mid epiboly-staged embryos, respectively, with different combinations of fluorescence and/or DIC layers shown. g, h Corresponding ventral views of later stage embryos undergoing epiboly. i Ventral view of an older, elongating embryo. j, k Ventral views of two embryos just prior to the onset of organogenesis. Note that for the original stack of confocal images shown as a projection in j, the 3a1 and 3a2 clones are spatially separated in the Z axis, making it possible to pseudocolor them separately, as labeled. Corresponding ventral views of embryo just prior to the onset of organogenesis are shown in l, m, n, o Left-lateral view of an embryo during organogenesis. cb ciliary band, ms mesenchyme. Other labels are the same as those used in Figs. 3 and 6. Scale barequals 50μm. [file 13227_2015_19_MOESM17_ESM.tiff]

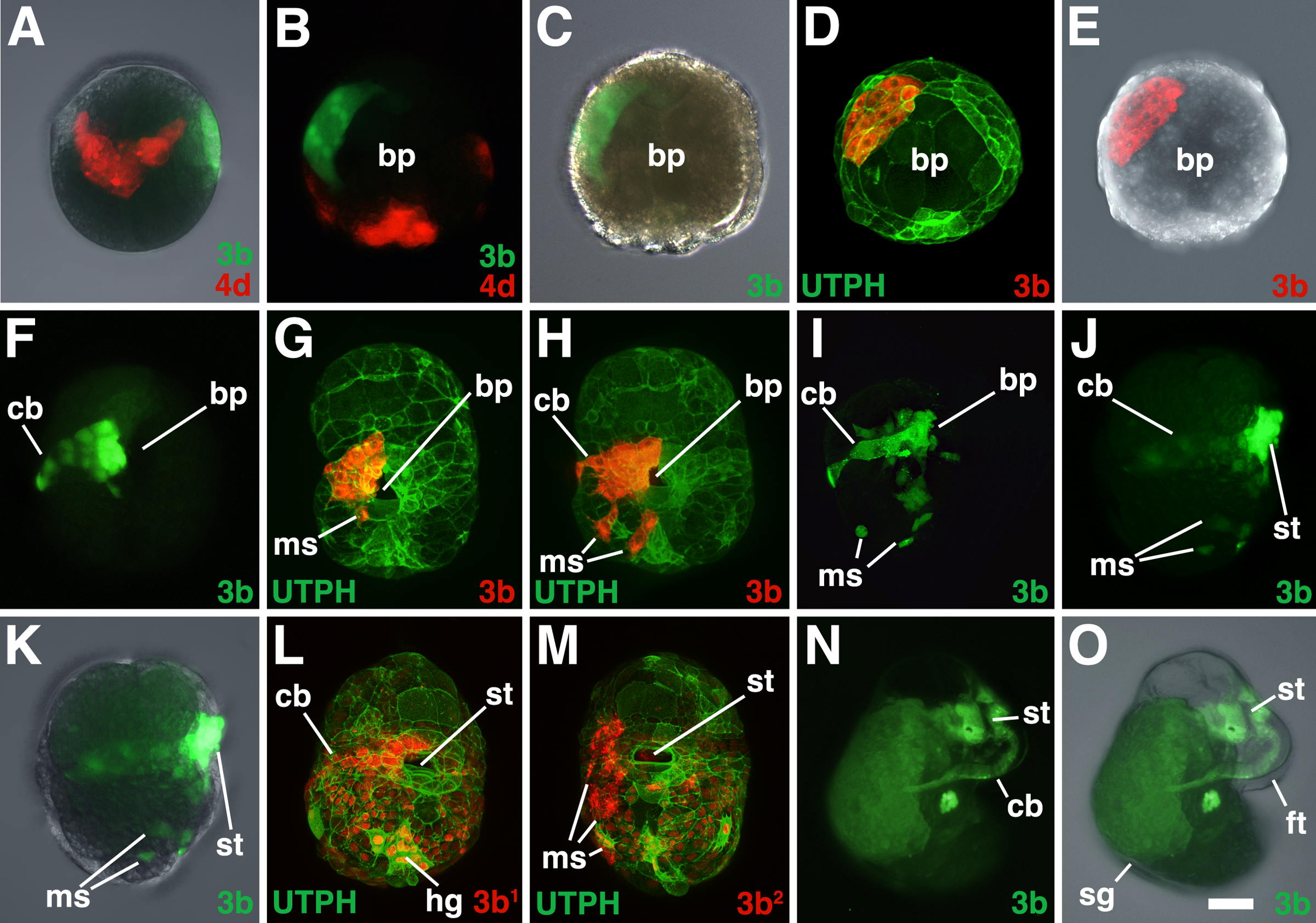

Supplement: Additional file 18: — Figure S8. Fates of micromere 3b, and its subclones, during gastrulation and organogenesis. Images of live embryos, with dextran and diI-labeled 4d, 3b, or 3b subclones, as indicated. In some cases, the zygote was previously injected with mRNAs coding for fluorescent fusion proteins for histone H2B-RFP (H2B) and/or the actin-binding domain of utrophin-GFP (UTPH) to visualize nuclei or cell outlines, respectively, where indicated. Animal pole is up in a. Anterior is up in b–o. a Lateral-dorsal view of an early epiboly-stage embryo. b-c, d-e show corresponding ventral (vegetal) views of early epiboly-staged embryos with different combinations of fluorescence and/or DIC layers shown. f Ventral view of embryo during later epiboly. g, h Show ventral views of two stages of mesenchyme migration. i Ventral view showing numerous mesenchyme cells. j, k Corresponding right-lateral views of embryos during organogenesis. l, m Ventral surface views. n, o Corresponding right-lateral views of older embryos during organogenesis. Labels are the same as those used in Figs. 3, 6, and 7. Scale bar equals 50 μm. [file 13227_2015_19_MOESM18_ESM.tiff]

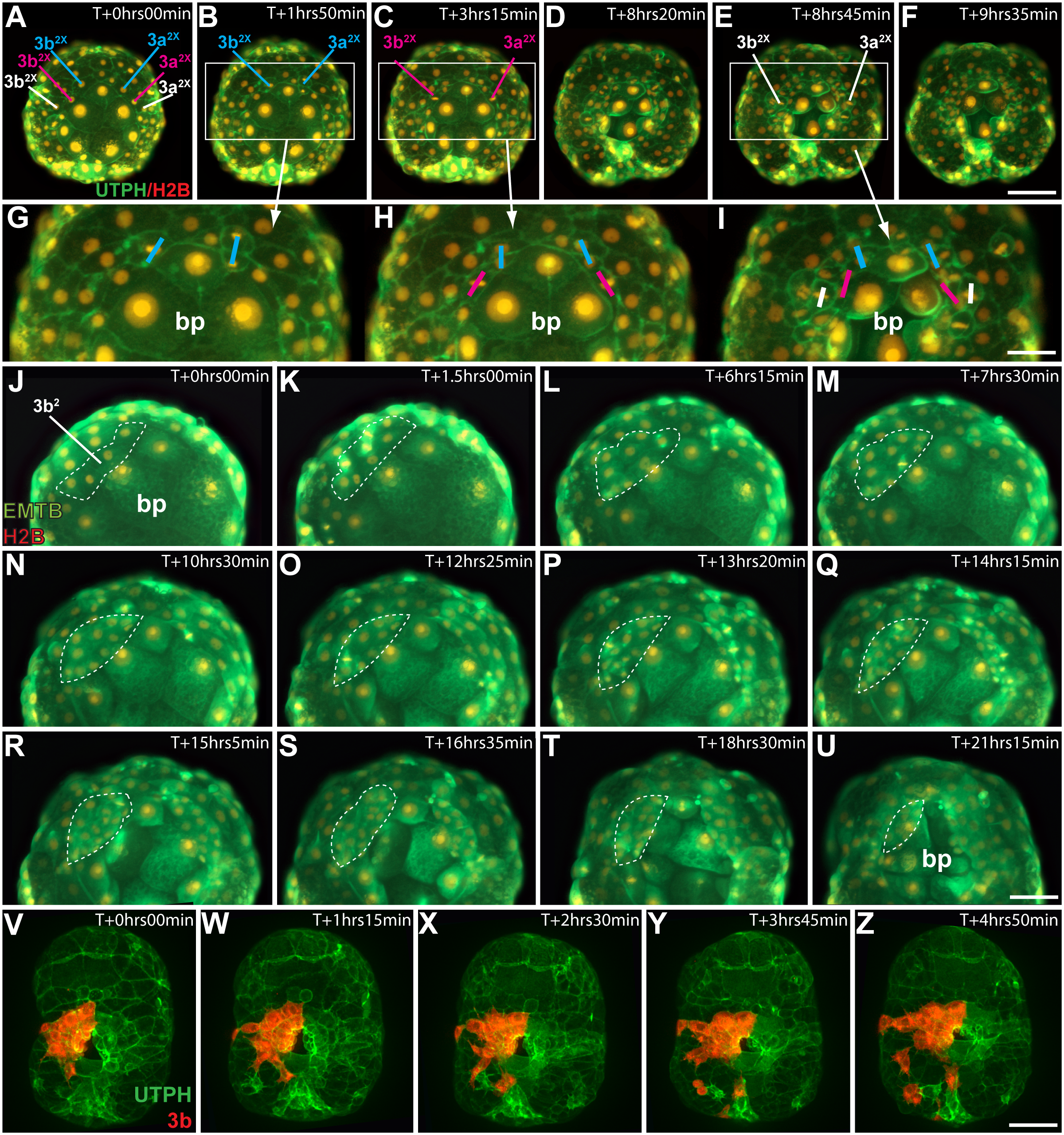

Supplement: Additional file 19: — Figure S9. Behavior of ectomesoderm (3a2, 3b2). a–i Time-lapse movie of an embryo injected with utrophin-GFP (UTPH) to mark cell outlines, and histone H2B-RFP (H2B) to mark nuclei, where indicated. Ventral view. bp blastopore. Several unidentified (x) daughter cells of 3a2, 3b2 are marked, in colors, to show the orientation of cell division and position of ectomesodermal precursor cells during the narrowing of the anterior blastopore lip. Scale bar in f equals 50 μm; scale bar in i equals 25 μm. j–u Frames from a timelapse movie of an embryo injected with ensconsin-GFP (EMTB) to mark microtubules, and histone-RFP (H2B) to mark nuclei. Ventral view. Dashed white lines outline the 3b2 clone. Scale bar in u equals 30 μm. v–z Spinning disk confocal frames from a time-lapse of an embryo expressing utrophin-GFP (UTPH) globally, and in which the 3b micromere was labeled with diI (red). Ventral view. Scale bar in z equals 50 μm. See also Additional files 6, 7, and 8. [file 13227_2015_19_MOESM19_ESM.tiff]

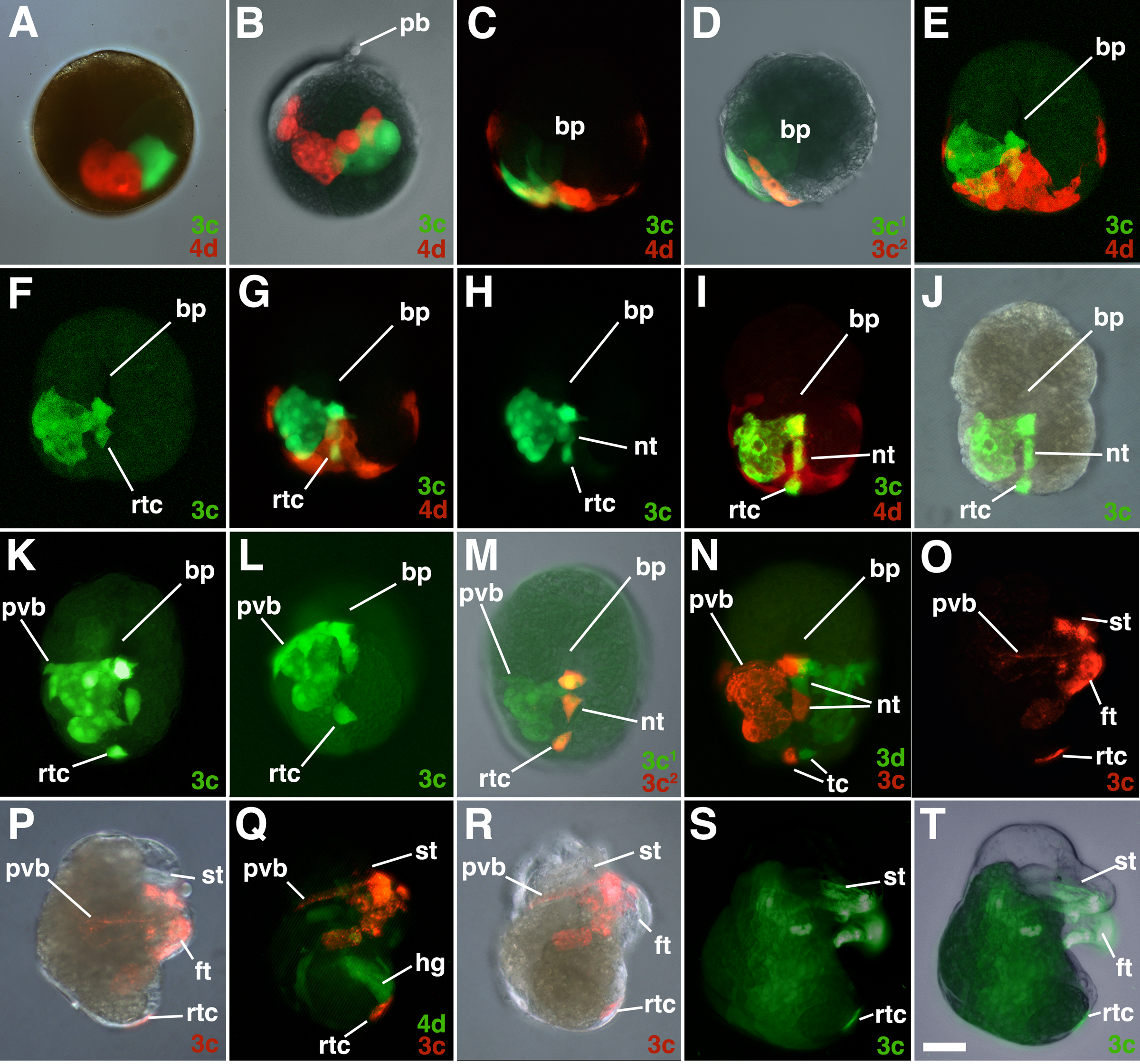

Supplement: Additional file 20: — Figure S10. Fates of micromere 3c, and its subclones, during gastrulation and organogenesis. Images of live embryos, with dextran and diI-labeled 4d, 3c, or 3c subclones, as indicated. Animal pole is up in a and b. Anterior is up in c–t. a, b Dorso-lateral views of early epiboly-stage embryos. c, d Ventral views of early epiboly-stage embryos. Corresponding ventral views of late epiboly-stage embryos are shown in e-f, g-h, i-j at successive stages of development with different combinations of fluorescence and/or DIC layers shown. k, l Corresponding ventral and posterior views of an elongating embryo. m, n Ventral views of embryos during elongation. Corresponding right-lateral views of embryos during organogenesis o-p, s-t. Note green background fluorescence is higher in s, t. (Q-R) Corresponding oblique, ventro-lateral views of an embryo during organogenesis. nt neurotroch, rtc right terminal cell, pvb posterior velar band. All other labels are the same as those used in Figs. 3 and 6. Scale bar equals 50 μm. [file 13227_2015_19_MOESM20_ESM.tiff]

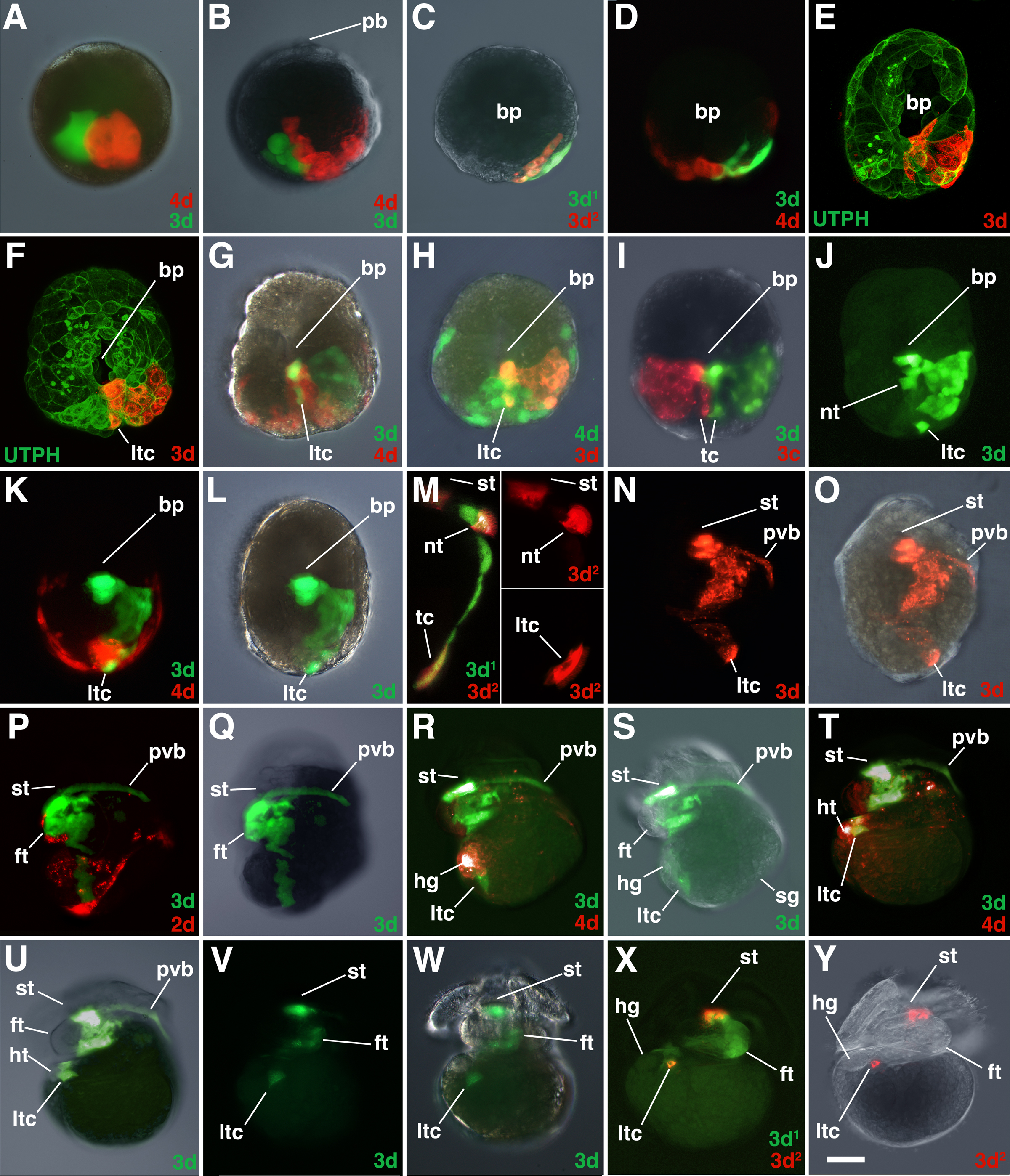

Supplement: Additional file 21: — Figure S11. Fates of micromere 3d, and its subclones, during gastrulation and organogenesis. Images of live embryos, with dextran and diI-labeled 4d, 3d, or 3d subclones, as indicated. In some cases, the zygote was previously injected with mRNAs coding for fluorescent fusion proteins for the actin-binding domain of utrophin-GFP (UTPH) and histone H2B-RFP to visualize nuclei or cell outlines, respectively, where indicated. Animal pole is up in a and b. Anterior is up in c–y. a, b Dorso-lateral views of early epiboly-stage embryos. c, d Ventral views of early epiboly stage embryos. e–j Ventral views of elongating embryos later during epiboly. k, l Corresponding ventral views of an embryo during elongation with different combinations of fluorescence and/or DIC layers shown. m Right-lateral higher magnification views of an elongating embryo with inserts showing some of the ventral ciliated cells (shallow confocal stacks centered at the ventral midline). n, o Corresponding ventral views of embryos during organogenesis. Corresponding left lateral views of embryos during organogenesis p-q, r-s, t-u. Corresponding ventral views of early veliger stage embryos v-w, x-y. ltc left terminal cell. All other labels are the same as those used in Figs. 3, 6, and 10. Scale bar equals 50 μm for a–l and n–y. Scale bar equals 25 μm for m and 20 μm for its inserts. [file 13227_2015_19_MOESM21_ESM.tiff]

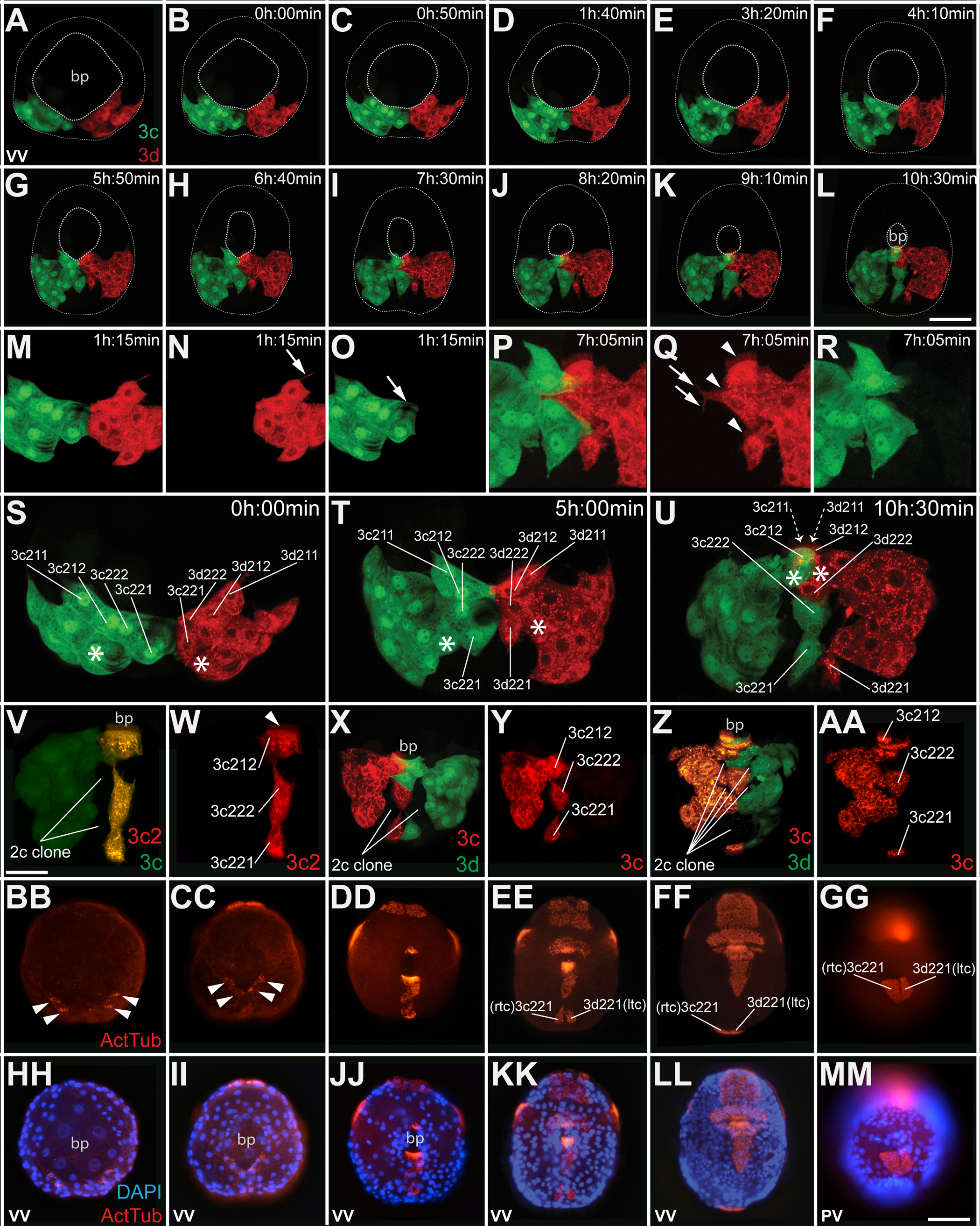

Supplement: Additional file 22: — Figure S12. Behavior of posterior blastopore lip cells undergoing convergence and extension (3c2, 3d2). a–aa Projected confocal Z slices of embryos injected with dextran or diI, into 3c, 3d or their subclones, as indicated. a Ventral view of an embryo at epiboly stage. b–u Frames from a time-lapse of the same embryo as shown in a. b–l Frames from a time-lapse movie of the same embryo undergoing convergence and extension to zipper the posterior blastopore (bp) closed. m–r Frames from the same movie showing membrane protrusions (arrows) and cilia (arrow heads) on the cells undergoing convergent extension (zippering). s, t Frames from the same movie showing that the cells undergoing convergence and extension are from the 3c2 and 3d2 cells. The asterisk (*) marks cells from the 3c1 and 3d1 clones that migrate anteriorly towards the ventral side of the stomodeum. In u, the 3c211 and 3d211 cells have entered the deeper parts of the mouth an are out of view of the stack, indicated by dashed lines. v–aa Live confocal images of post-zippering-stage embryos showing the movement of the 3c221 and 3d221 cells posteriorly, between cells of the 2d clone. bb–mm Images of fixed embryos stained for acetylated tubulin (to mark cilia) and DAPI (to mark DNA). bb, cc Shows ventral views (vv) of embryos at the early stages of convergent extension and arrowheads point to cilia on cells at the posterior edge of the blastopore. dd–ff Ventral views of embryos during elongation. The ciliated left and right terminal cells (ltc, rtc) are labeled. gg Posterior view (pv) of the same embryo shown in ff. Scale bar in l and mm are equal to 50 μm; scale bar in v equals 20 μm. See also Additional files 9 and 10. [file 13227_2015_19_MOESM22_ESM.tiff]

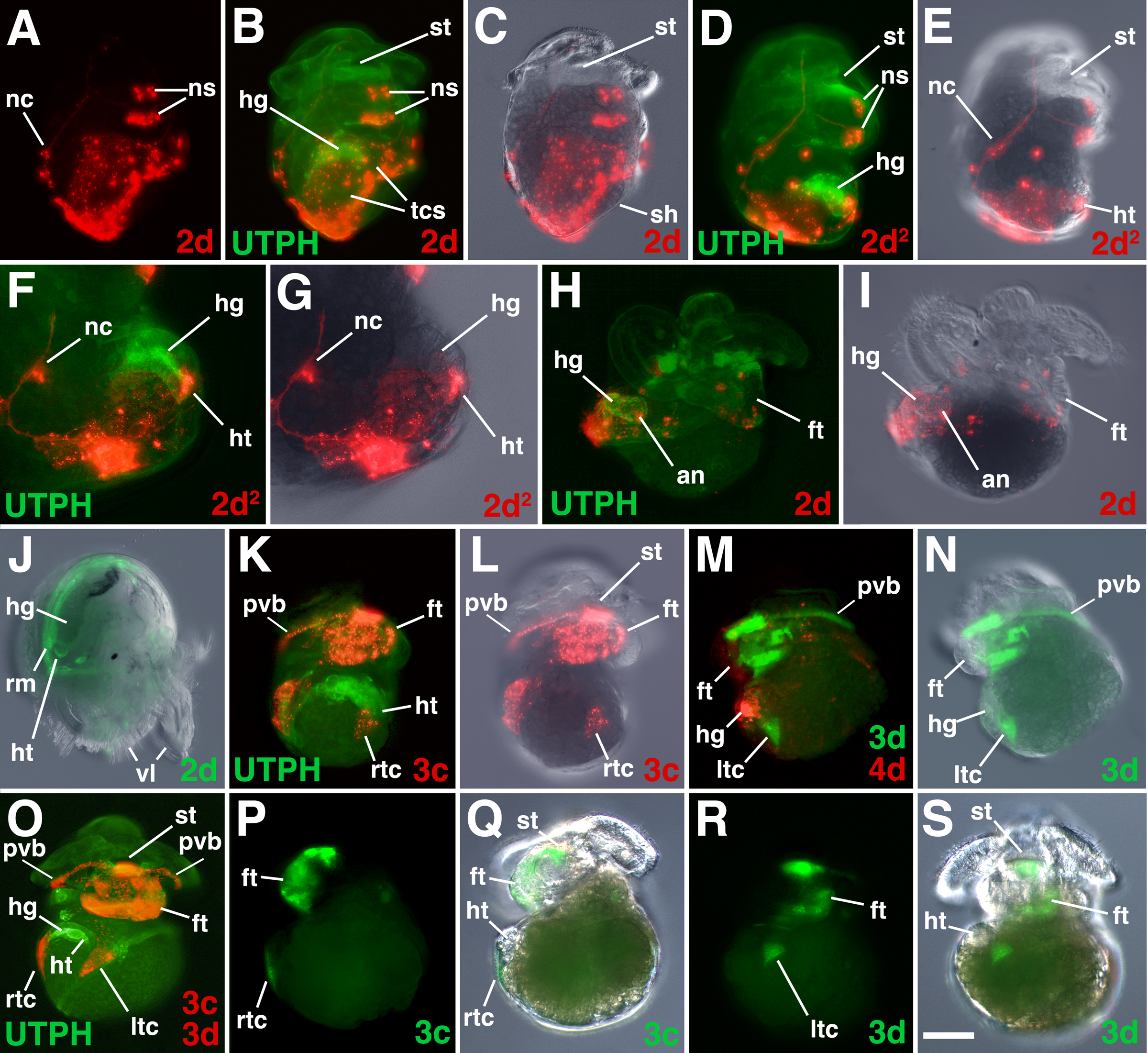

Supplement: Additional file 23: — Figure S13. Origin of the anus (2d2). Images of live embryos, with dextran and diI-labeled 4d, 3d, 3c, 2d, or 2d subclones, as indicated. In some cases, the zygote was previously injected with mRNAs coding for fluorescent fusion protein for the actin-binding domain of utrophin-GFP (UTPH) to visualize cell outlines, where indicated. Anterior is up in all cases. a–c Corresponding ventral views of embryos during organogenesis with different combinations of fluorescence and/or DIC layers shown. d, e Corresponding right-lateral (oblique-ventral) views of embryo during organogenesis. f, g Corresponding higher magnification right-lateral views of the posterior end of an embryo during organogenesis. h, i Corresponding ventral views of veliger stage embryo after the anus as opened. j Ventral view of a veliger stage embryo just before the anus opens. Note that the hindgut is somewhat swollen. k, l Corresponding ventral views of pre-veliger stage embryos. m, n Corresponding (oblique-ventral) left-lateral views of the pre-veliger stage embryos. o Ventral view of early veliger. p, q Corresponding (oblique-ventral) left-lateral views of an early veliger. r, s Corresponding ventral views of an early veliger. an anus, nc neural cell, ns neurosensory cell, rm right mantle, tcs terminal cells (called tc in Fig. 6). All other labels are the same as those used in Figs. 3, 4, 10, and 11. Scale bar equals 50 μm for a–e, h–s. Scale bar equals 25 μm in f, g. [file 13227_2015_19_MOESM23_ESM.tiff]

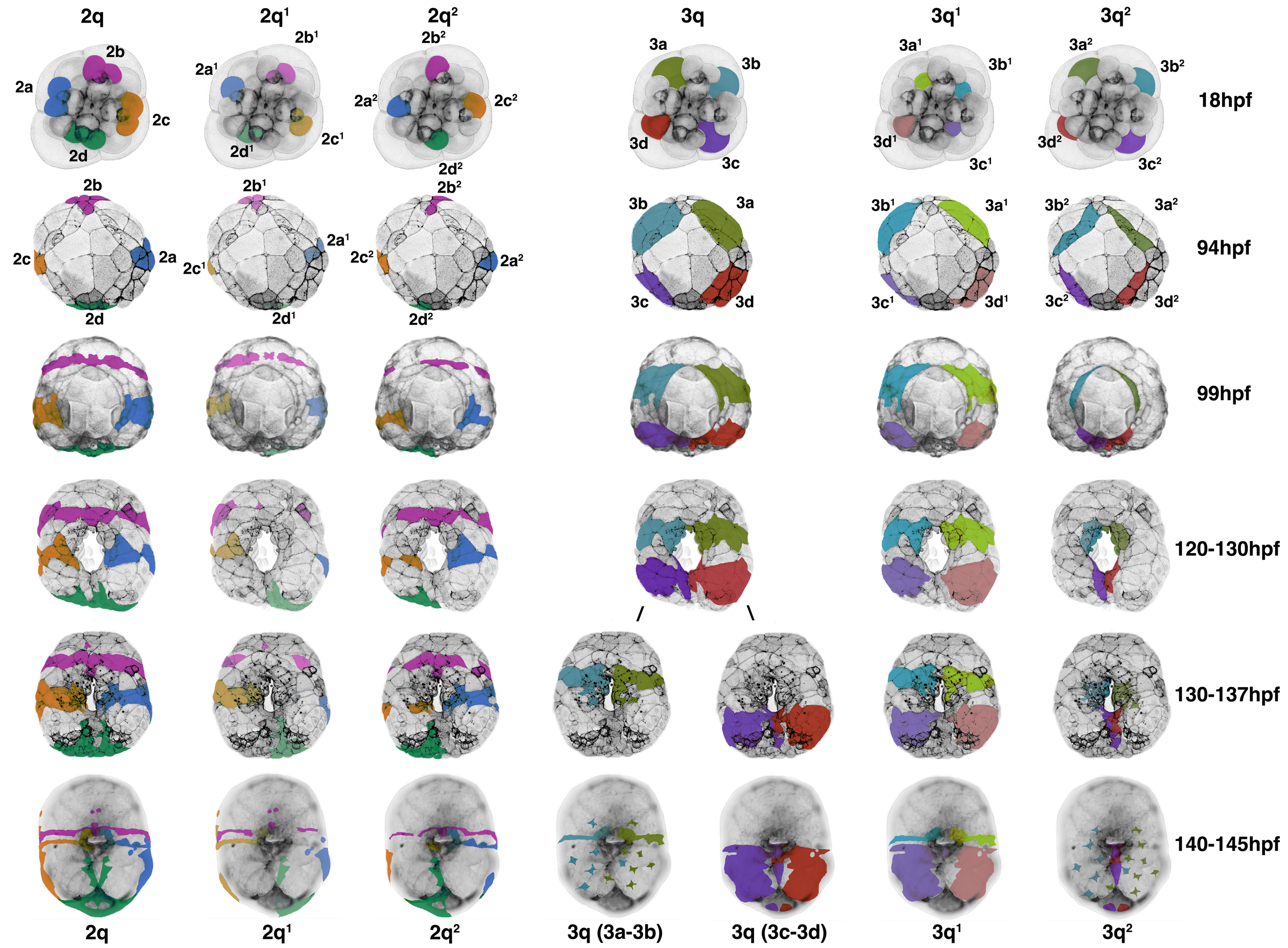

Supplement: Additional file 24: — Figure S14. Summary of second and third quartet clones. Columns show clones, colored as labeled, and according to those shown in Figs. 1 and 15. Rows show time points as indicated at the far right. Top row shows animal views; all other rows show ventral views. The smaller, irregularly shaped, stippled cells of the 3a2 and 3b2 clones represent ecto-mesenchyme located below the ectoderm [file 13227_2015_19_MOESM24_ESM.tiff]

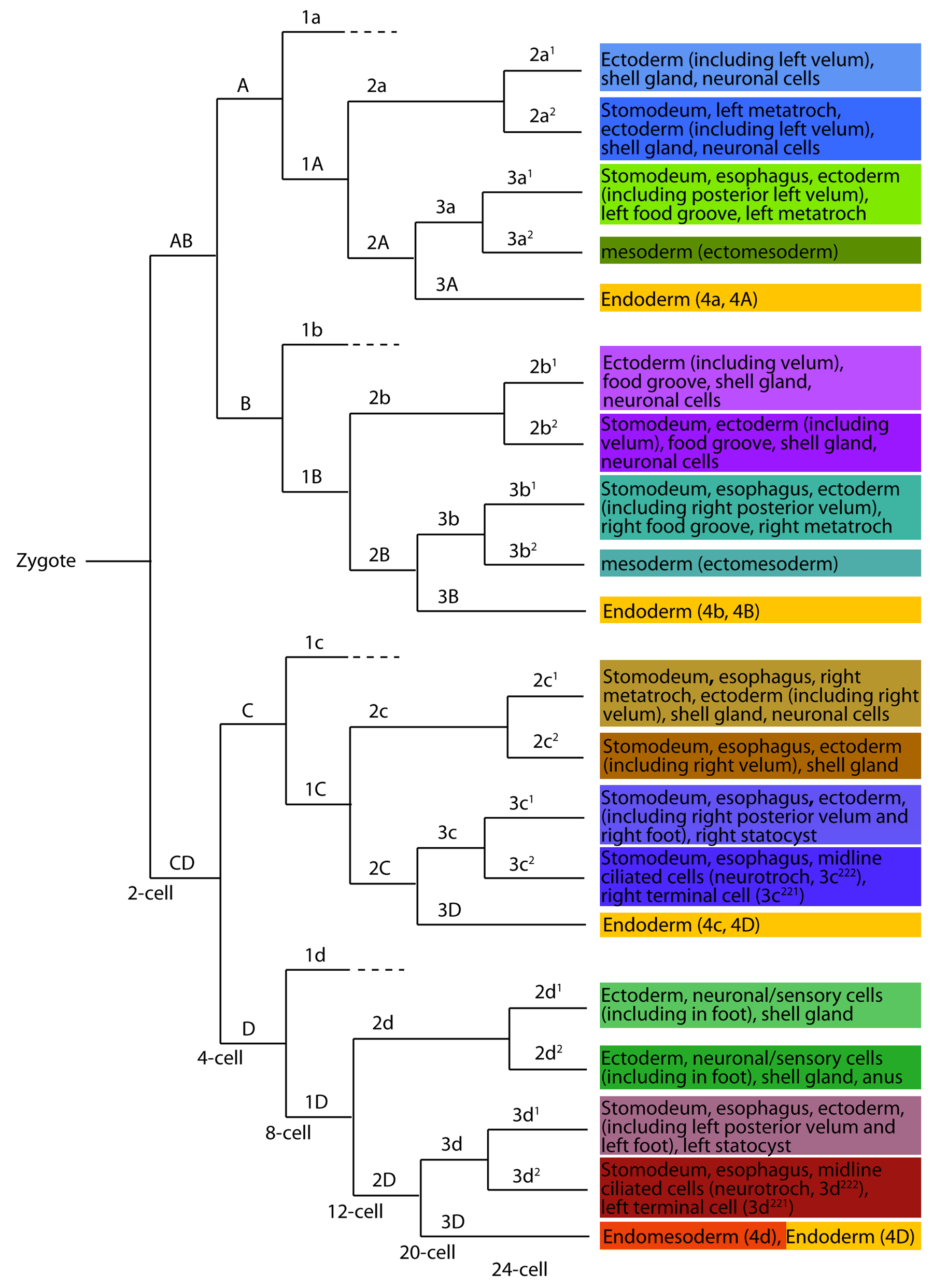

Supplement: Additional file 25: — Figure S15. Lineage diagram of second and third quartet micromeres and third quartet macromeres. Colors correspond to those given in Figs. 1 and 14. [file 13227_2015_19_MOESM25_ESM.tiff]

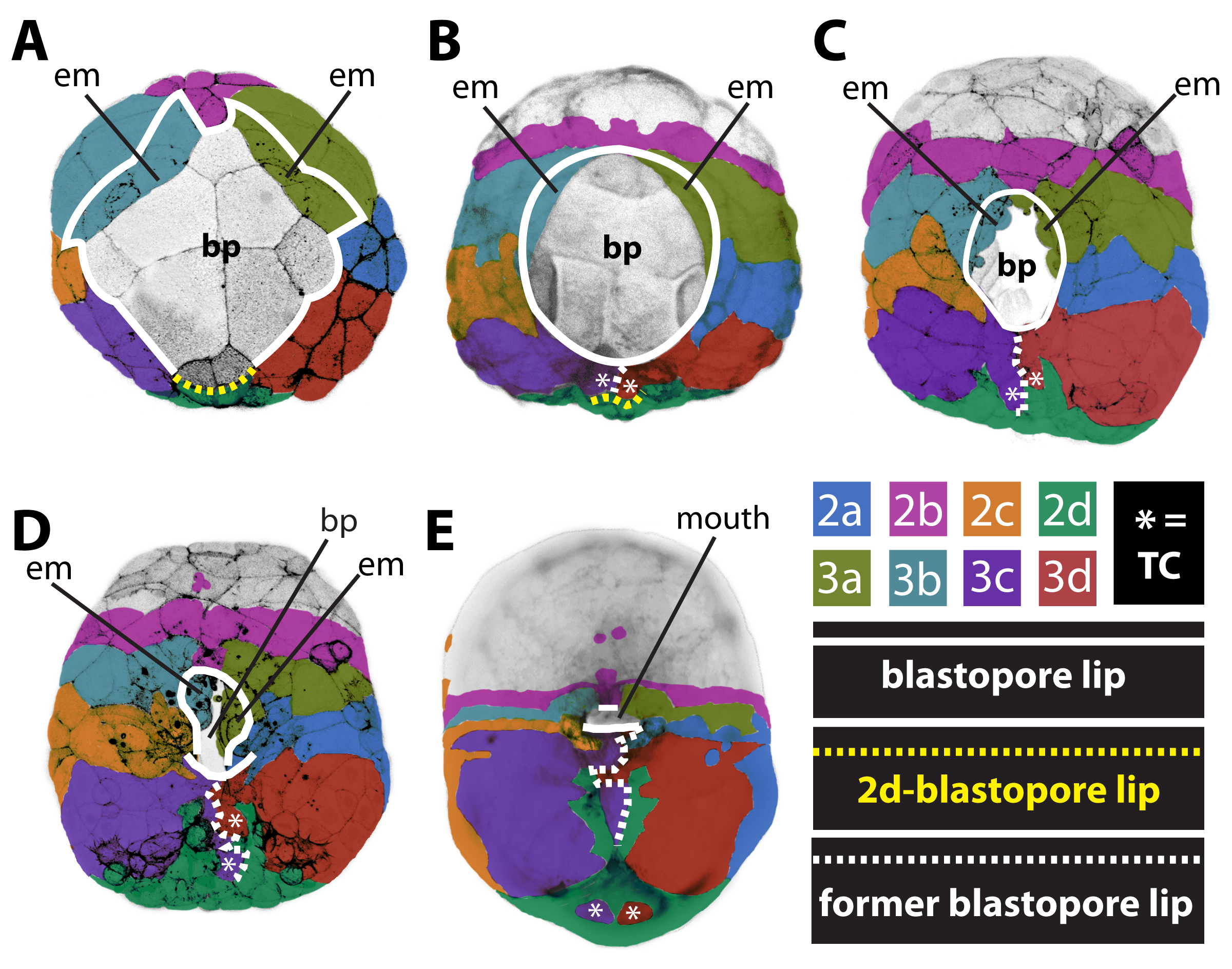

Supplement: Additional file 26: — Figure S16. Morphogenesis of the blastopore lip. a–e Vegetal/ventral views during gastrulation with the future anterior at the top of the figure. Time points are the same as those shown in rows 2–6 in Figs. 1 and 14: a ~94 hpf, b ~99 hpf, c ~120–130 hpf, d ~130–137 hpf, e ~140–145hpf. The coloring shows the relative clonal contributions of cells within the embryo and to the blastopore lip. The blastopore lip is marked in each panel by a solid white line. The blastopore lip marks the boundary between the ectodermal micromere cap and the endoderm/endomesoderm/ectomesoderm. As gastrulation proceeds, some cells leave the blastopore lip, but remain on the surface, and these are marked by a dashed white line. The 2d cells are marked by a dashed yellow line in a, b, but are more difficult to follow in time points c–e and thus are not shown. The 3c2- and 3d2-derived terminal cells (TC) are marked with an asterisk to follow their migration after they leave the blastopore lip. Some cells of the blastopore lip move internally into the blastocoel/archenteron, and the solid white line can no longer be seen in some areas d, e. bp blastopore, em ectomesoderm (derived from 3a2 and 3b2). [file 13227_2015_19_MOESM26_ESM.tiff]
